# Supplementary material for: Order–order assembly transition-driven polyamines detection based on iron−sulfur complexes
Source: Commun Chem. 2023 Jul 7;6:146. doi: 10.1038/s42004-023-00942-1 (PMC10328931; doi:10.1038/s42004-023-00942-1)
Supplement: Supplementary file 2 — Supplementary Information [file 42004_2023_942_MOESM2_ESM.pdf]

Supplementary Information

Order-Order Assembly Transition-Driven Polyamines Detection Based on Iron–Sulfur Complexes

Yahui Zhang,[1] Xiangyu Zhao,[2] Yue Qin,[1] Xiaopei Li,[1] Yongxin Chang,[1] Zhenqiang Shi,[1] Mengyuan Song,[1] Wenjing Sun,[1] Jie Xiao,[1] Zan Li,[1] and Guangyan Qing*[1,3]

[1]Key Laboratory of Separation Science for Analytical Chemistry, Dalian Institute of Chemical Physics, Chinese Academy of Sciences, 457 Zhongshan Road, Dalian 116023, P. R. China.

[2]Sixth Laboratory, Sinopec Dalian (Fushun) Research Institute of Petroleum and Petrochemicals, 96 Nankai Road, Dalian 116045, P. R. China.

[3]College of Chemistry and Chemical Engineering, Wuhan Textile University, 1 Sunshine Road, Wuhan 430200, P. R. China.

Table of Contents

I. General Information S3

II. Supplementary Methods S4

III. Crystal Information S6

IV. NMR Spectra S22

V. EPR Spectra S25

VI. UV-vis Spectra S28

VII. HRMS Spectra S41

VIII. Electrochemical Spectra S44

IX. IR Spectra S46

X. Morphological Data S49

XI. Cyclic Detection S53

I. General Information

Materials. Partial manipulations were carried out under an argon atmosphere by using standard Schlenk techniques and Mikrouna argon-filled glove box. THF and CH_2Cl_2 were dried by conventional procedures and distilled under nitrogen before use. Anhydrous $FeCl_2$ (Aldrich), pentamethylcyclopentadienyl (Energy Chemical), ^{n}BuLi (Heowns), $S(CH_2CH_2SH)_2$ (Aldrich), $ZnCl_2$ (Heowns), $MnCl_2$ (Energy Chemical), $PdCl_2$ (Energy Chemical), $NiCl_2$ (Aladdin), CuCl (Aladdin), $AlCl_3$ (Macklin), $TiCl_4$ (Macklin), VCl_3 (Innochem), varous organic amines (Aldrich), various solvents (Xilong Scientific), lanthanide elements (Innochem) and ^{n}Bu_4NPF_6 (Aladdin) were obtained from commercial suppliers and used without further purification. CD_2Cl_2, CD_3CN, DMSO-d_6 and D_2O were purchased from Alfa Aesar and used dried with a 4Å molecular sieve. Complex [Cp*Fe(η^3-tpdt)] was prepared according to the previous literature.[1]

Instruments. ESI-HRMS data were recorded on an HPLC/Q-Tof mass spectrometer. Infrared spectra were recorded on a NICOLET iS50 ATR spectrometer. ^{1}H and ^{13}C NMR spectra were recorded on a Brüker 400 M Ultra Shield spectrometer. Chemical shifts (δ) were given in parts per million relative to CD_2Cl_2 (5.32 ppm for ^{1}H), CD_3CN (1.94 ppm for ^{1}H), DMSO-d_6 (2.50 ppm for ^{1}H; 39.52 ppm for ^{13}C) and D_2O (4.79 ppm for ^{1}H). Low-field NMR spectra were recorded on a Suzhou Niumag VTMR20-010V-I spectrometer. EPR spectra were recorded at room temperature on a JEOL JES-FE3AX spectrometer. UV-vis spectra were recorded on a PerkinElmer Lambda 365 spectrometer. Electrochemical data were obtained on a dual display potentiostat (CHI760E, Shanghai Chenhua). Particle size distribution measurements were recorded on a Zetasizer Nano instrument. SEM measurements were carried out on a JEOL JSM−7800 F instrument and the elemental compositions were analyzed by an X−ray energy dispersive spectroscopy (EDS, X−Max50, Oxford).

Single crystal data were obtained on a Brüker SMART APEX CCD diffractometer with graphite-monochromated Mo Kα radiation (λ = 0.71073 Å). Empirical absorption corrections were performed using the SADABS program.[2]

Structures were solved by direct methods and refined by a full-matrix least-squares method based on all data using F^2 with SHELX2014.[3] All of the nonhydrogen atoms were refined anisotropically. All of the hydrogen atoms were generated and refined in ideal positions. Crystal data and collection details for **C1**, **C3**, and **C4** are given in Table S1; Crystal data and collection details for **N1**, **N2**, and **N3** are given in Table S2. Powder XRD patterns were collected by a Shimadzu XRD−7000S Powder X-ray diffractometer with 1.5418 Å Cu Kα radiation (scan speed: 5°/min; scan range: 5−80°).

The single-crystal data generated in this study have been deposited in The Cambridge Crystallographic Data Center under accession code CCDC-2171251 (for **C1**), CCDC-2171252 (for **C3**), CCDC-2171267 (for **N1**), CCDC-2178630 (for **N2**), and CCDC-2178629 (for **N3**). The cif files of **C1**, **C3**, **N1**, **N2**, and **N3** can be found in Supplementary Data 1−5, and their corresponding checkcif files are shown in Supplementary Data 6−10, respectively.

II. Supplementary Methods

UV-vis measurements. The UV-vis spectra of **C1−C6** (0.33 mM) were recorded before and after the addition of various reagents (0 to 0.4 mM or 15 mM) in CH_2Cl_2 (3 mL), with a wavelength ranging from 200 to 900 nm. The assay temperature was kept at 20 °C and the cuvette was closed with a polytetrafluoroethylene cap to avoid CH_2Cl_2 volatilization.

EPR measurements. The electron spin states of all the complexes were recorded under a liquid state at the concentration of 10 mM. EPR data of **C1−C3**, **C6** as well as the product of **C1** after the addition of cyclen (12 mM) was obtained in CH_2Cl_2 solution, while **C4** and **N3** were performed in CH_3CN and CH_3OH solution, respectively. These liquid samples were encapsulated in hollow glass tubes with a diameter of 1.5 mm, and then placed in quartz tubes for testing at 20 °C.

Electrochemical tests. Electrochemical data were carried out in a three-electrode cell under nitrogen at 20 °C. An airbag equipped with nitrogen was attached to the

electrolytic cell to ensure an inert gas environment during the testing. The working electrode was a glassy carbon disk (diameter 3 mm), the reference electrode was a nonaqueous Ag/Ag^{+} electrode, and the auxiliary electrode was a platinum wire, which was all purchased from Shanghai Vietnamese Magnetic Electronics. The supporting electrolyte was 0.1 M $^{n}Bu_4NPF_6$ in CH_3CN (10 mL). DPV data of **C1** (1 mM) were recorded before and after the addition of different concentrations (60, 150, 300, 600, 900, 1200, 1500, and 1800 μM) of diethylenetriamine, cyclen, 4,4'-bipyridine and ^{n}propylpamine. The potential range was – 0.8 ~ – 1.2 V and the amplitude was 0.05 V. In CV experiments, the scan rate was 50 $mV \cdot S^{-1}$ and the potential range was – 1.5 ~ 1.1 V. The current was reported in μA and potential was reported in V against Ag/AgCl couple.

Hydrated diameter distribution measurements. Particle size distribution measurements were recorded on a Zetasizer Nano instrument. The hydrated diameter distribution of **C1** (0.10 mM) in CH_2Cl_2 before and after the addition of cyclen (0.12 mM) was obtained smoothly. The detection temperature was 20 °C and the cuvette was closed with a polytetrafluoroethylene cap to avoid CH_2Cl_2 volatilization. The diameter distribution of M–polyamines (M = Zn, Fe, Mn) was calculated by the Nano Measurer software based on their SEM images.

SEM measurements. For liquid samples, after the mixing of iron-sulfur complexes and organic amines, these mixtures were aspirated several times with a pipette gun and let for 5 minutes. Then SEM samples were made by dropping several drops of CH_2Cl_2 solution (3.3 mM) on a clean silicon wafer, evaporating the solvent in the open environment. For solid samples, SEM samples were prepared to stick the powder directly onto the carbonaceous tape.

III. Crystal Information

Table S1. Crystal data and structural refinement for complexes **C1**, **C3**, and **C4**

Compound	**C1**	**C3**	**C4**
Formula	$C_{14}H_{23}Cl_2FeS_3Zn$	$C_{14}H_{23}Cl_2FeS_3Mn$	$C_{36}H_{58}PdFe_2P_2F_{12}S_6N_4$
Formula weight	479.62	469.19	1247.26
Crystal dimensions (mm^3)	0.21×0.16×0.11	0.20×0.15×0.11	0.40×0.29×0.18
Crystal system	Monoclinic	Monoclinic	Orthorhombic
Space group	P2(1)	P2(1)	Fdd2
a (Å)	8.683(3)	8.771(2)	16.8637(16)
b (Å)	14.267(6)	14.128(3)	52.655(6)
c (Å)	16.095(6)	15.687(4)	11.3865(16)
α (°)	90.00	90.00	90.00
β (°)	79.403(7)	101.102(9)	90.00
γ (°)	90.00	90.00	90.00
Volume (Å^3)	1959.8(13)	1907.6(8)	10111(2)
Z	4	4	8
T (K)	296(2)	120(2)	293(2)
D_{calcd} (g cm^{-3})	1.626	1.634	1.639
μ (mm^{-1})	2.547	2.021	1.305
F (000)	980	960	5072
No. of rflns. collected	10063	21523	16340
No. of indep. rflns. /R_{int}	6793 / 0.0727	6525 / 0.0958	5765 / 0.0385
No. of obsd. rflns. [$I_0 > 2\sigma(I_0)$]	6066	5722	4849
Data / restraints / parameters	6793 / 253 / 379	6525 / 1 / 379	5765/ 1 / 288
R_1 / wR_2 [$I_0 > 2\sigma(I_0)$]	0.0576 / 0.1613	0.0389/ 0.0868	0.0316 / 0.0665
R_1 / wR_2 (all data)	0.0642 / 0.1681	0.0498 / 0.0898	0.0422 / 0.0710
GOF (on F^2)	1.092	1.009	1.008
Largest diff. peak and hole (e Å^{-3})	0.985 / −0.700	0.639 / −0.436	0.331 / −0.333
CCDC No.	2171251	2171252	1414702

Table S2. Crystal data and structural refinement for complexes **N1**, **N2**, and **N3**

Compound	**N1**	**N2**	**N3**
Formula	$C_8H_{20}Cl_2N_4Zn$	$C_{10}H_8Cl_2FeN_2$	$C_8H_{20}Cl_2N_4Mn$
Formula weight	308.55	282.93	298.12
Crystal dimensions (mm^3)	0.15×0.15×0.15	0.20×0.20×0.15	0.20×0.20×0.15
Crystal system	Orthorhombic	Monoclinic	Triclinic
Space group	P−1	I 2/a	P−1
a (Å)	9.3811(8)	6.9504(8)	9.4344(3)
b (Å)	9.3810(8)	9.1243(10)	9.4345(3)
c (Å)	7.5537(7)	16.525(3)	7.7669(3)
α (°)	90.00	90.00	89.999
β (°)	90.00	90.067(6)	90.001
γ (°)	90.00	90.00	90.001
Volume ($Å^3$)	664.76(10)	1048.0(2)	691.32(4)
Z	2	4	2
T (K)	120(2)	295(2)	295(2)
D_{calcd} (g cm^{-3})	1.541	1.793	1.432
μ (mm^{-1})	2.226	1.910	1.319
F (000)	320	568	310
No. of rflns. collected	10219	1201	10564
No. of indep. rflns. /R_{int}	2958 / 0.0577	1201 / 0.0601	2422 / 0.0252
No. of obsd. rflns. [$I_0 > 2\sigma(I_0)$]	2558	1066	2107
Data / restraints / parameters	2958 / 0 / 217	1201 / 0 / 58	2422 / 0 / 209
R_1 / wR_2 [$I_0 > 2\sigma(I_0)$]	0.0257 / 0.0627	0.0519 / 0.1413	0.0191 / 0.0423
R_1 / wR_2 (all data)	0.0317 / 0.0655	0.0579 / 0.1448	0.0244 / 0.0447
GOF (on F^2)	1.037	1.117	1.055
Largest diff. peak and hole (e $Å^{-3}$)	0.317 / −0.345	0.750 / −0.970	0.142 / −0.151
CCDC No.	2171267	2178630	2178629

Figure S1. ORTEP diagram of **C1**

Thermal ellipsoids are shown at a 50% probability level. All hydrogen atoms on carbons are omitted for clarity.

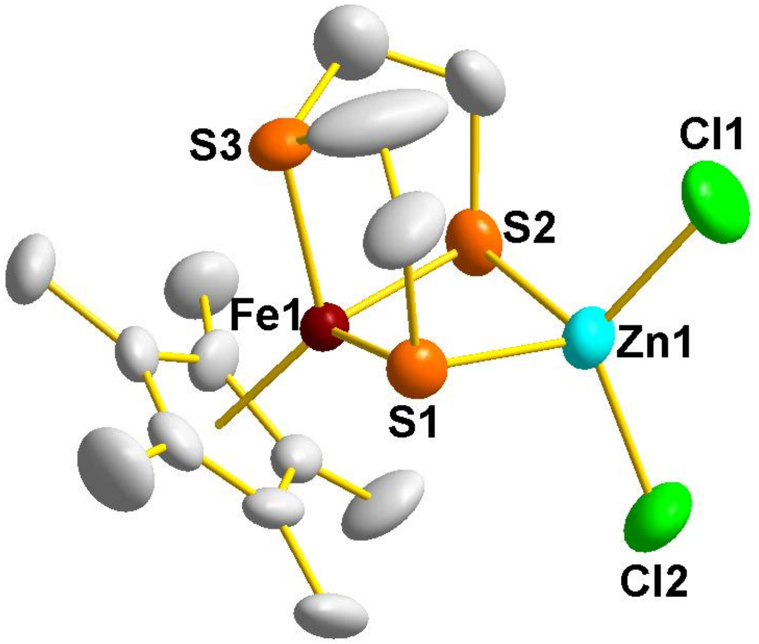

Table S3. Selected bond distances and angles for **C1**

Distances (Å)			
Fe1···Zn1	2.993(2)	Zn1−S2	2.411(3)
Fe1–S1	2.272(3)	Zn1–Cl1	2.222(4)
Fe1–S2	2.280(3)	Zn1–Cl2	2.228(4)
Fe1–S3	2.244(3)	Fe1–Cp*	1.747(1)
Zn1–S1	2.422(3)		
Angles (°)			
S1–Fe1–S2	102.61(12)	S1–Zn1–C11	112.75(16)
S1–Fe1–S3	88.46(13)	S1–Zn1–C12	107.39(16)
S2–Fe1–S3	88.16(13)	S2–Zn1–C11	107.78(16)
Fe1–S1–Zn1	79.16(10)	S2–Zn1–C12	112.44(18)
Fe1–S2–Zn1	79.23(11)	Cl1–Zn1–C12	119.19(19)

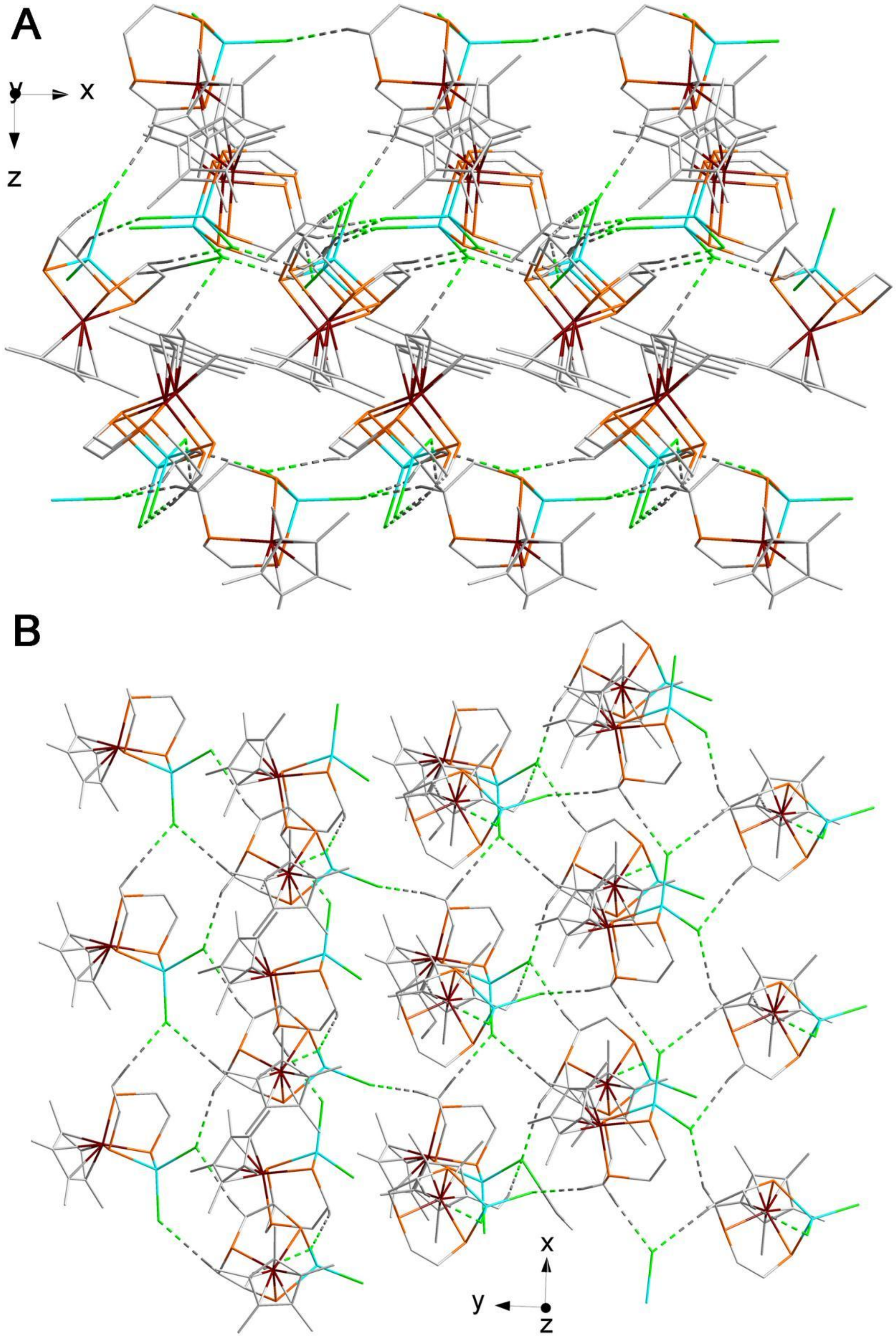

Figure S2. Crystal packing maps of **C1** (CCDC 2171251) along y- (A) and z-axes (B).

Figure S3. ORTEP diagram of **C2**

Thermal ellipsoids are shown at a 50% probability level. All hydrogen atoms on carbons are omitted for clarity.

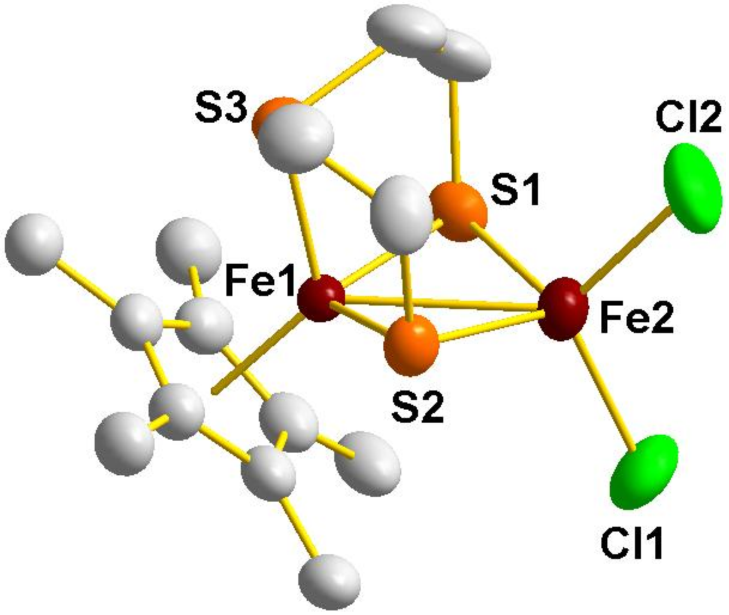

Table S4. Selected bond distances and angles for **C2**

Distances (Å)			
Fe1–Fe2	2.7722(14)	Fe2–S2	2.343(2)
Fe1–S1	2.245(2)	Fe2–Cl1	2.247(3)
Fe1–S2	2.242(2)	Fe2–Cl2	2.263(2)
Fe1–S3	2.227(2)	Fe1–Cp*	1.760(1)
Fe2–S1	2.339(2)		
Angles (°)			
S1–Fe1–S2	106.45(8)	S1–Fe2–C11	109.00(10)
S1–Fe1–S3	88.19(8)	S1–Fe2–C12	111.78(10)
S2–Fe1–S3	88.64(8)	S2–Fe2–C11	114.16(10)
Fe1–S1–Fe2	74.40(7)	S2–Fe2–C12	106.66(10)
Fe1–S2–Fe2	74.36(7)	C11–Fe2–C12	114.13(11)

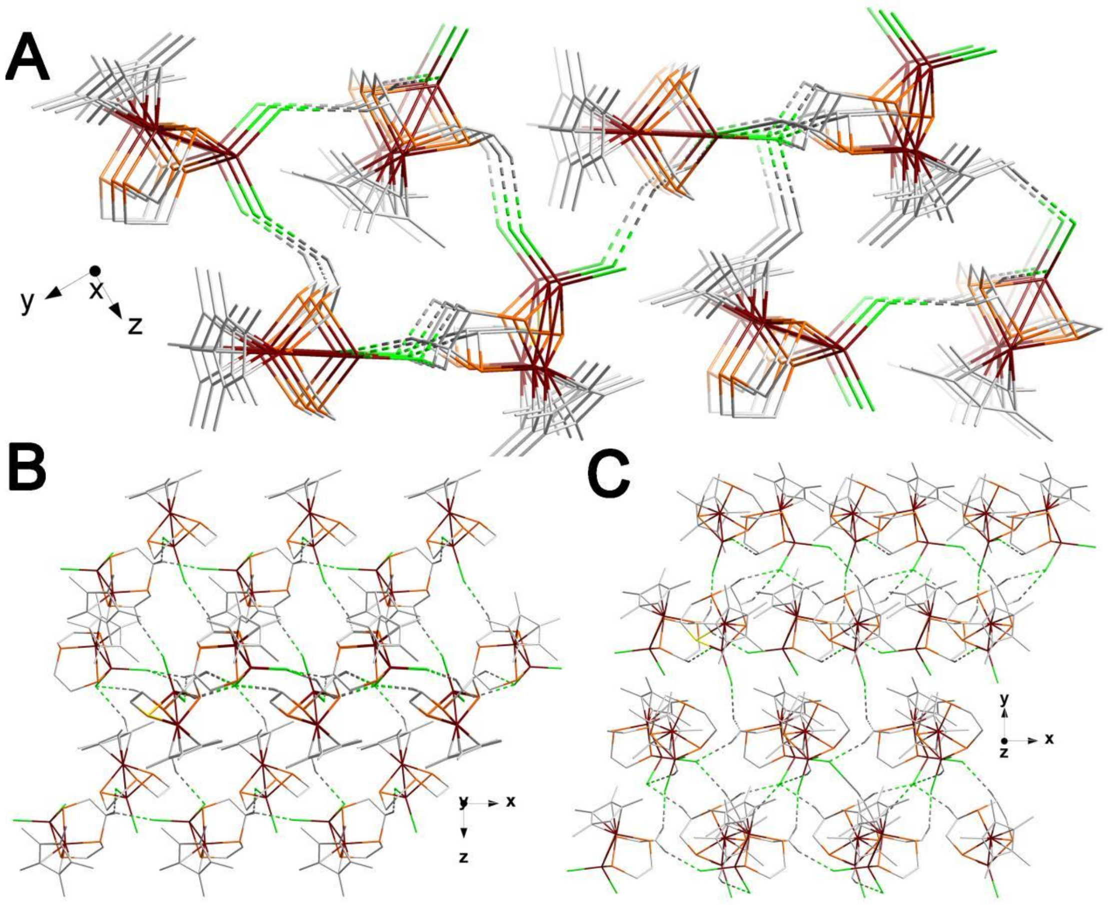

Figure S4. Crystal packing maps of **C2** along x- (A), y- (B), and z-axes (C).

Figure S5. ORTEP diagram of **C3**

Thermal ellipsoids are shown at a 50% probability level. All hydrogen atoms on carbons are omitted for clarity.

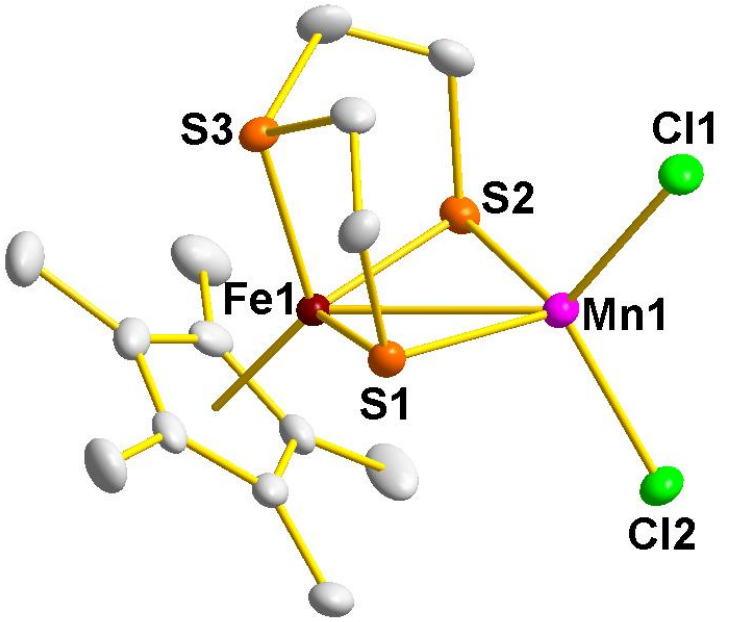

Table S5. Selected bond distances and angles for **C3**

Distances (Å)			
Fe1–Mn1	2.850(1)	Mn1–S2	2.415(2)
Fe1–S1	2.248(2)	Mn1–Cl1	2.310(2)
Fe1–S2	2.262(2)	Mn1–Cl2	2.309(2)
Fe1–S3	2.234(2)	Fe1–Cp*	1.749(1)
Mn1–S1	2.408(2)		
Angles (°)			
S1–Fe1–S2	107.88(7)	S1–Mn1–C11	110.90(8)
S1–Fe1–S3	87.79(7)	S1–Mn1–C12	107.62(8)
S2–Fe1–S3	88.98(7)	S2–Mn1–C11	110.40(8)
Fe1–S1–Mn1	75.40(6)	S2–Mn1–C12	117.44(7)
Fe1–S2–Mn1	75.00(6)	Cl1–Mn1–C12	111.43(7)

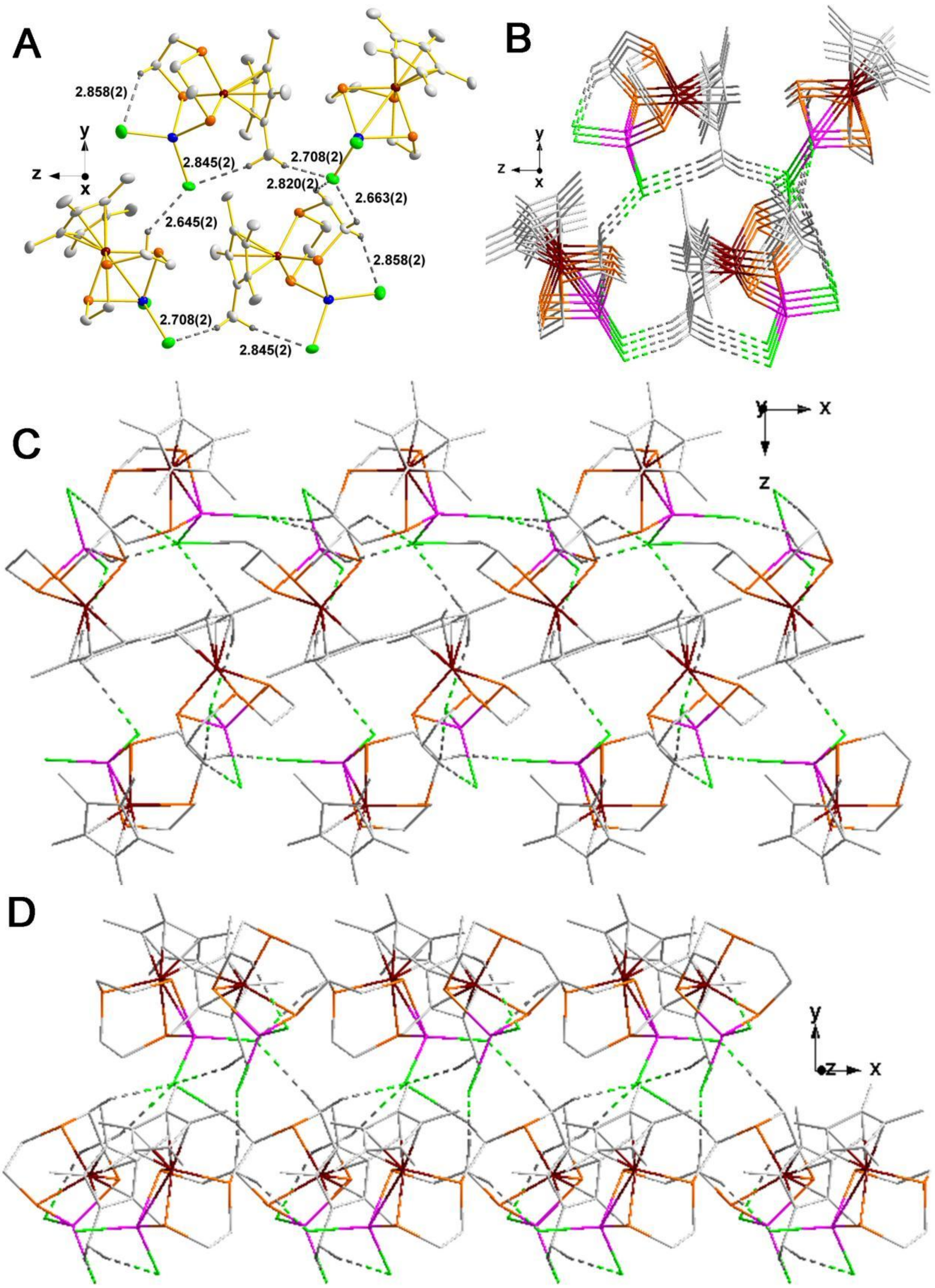

Figure S6. Crystal packing maps of **C3** (CCDC 2171252) along x- (A), y- (B), and z-axes (C).

Figure S7. ORTEP diagram of **C4**

Thermal ellipsoids are shown at a 50% probability level. All hydrogen atoms on carbons are omitted for clarity.

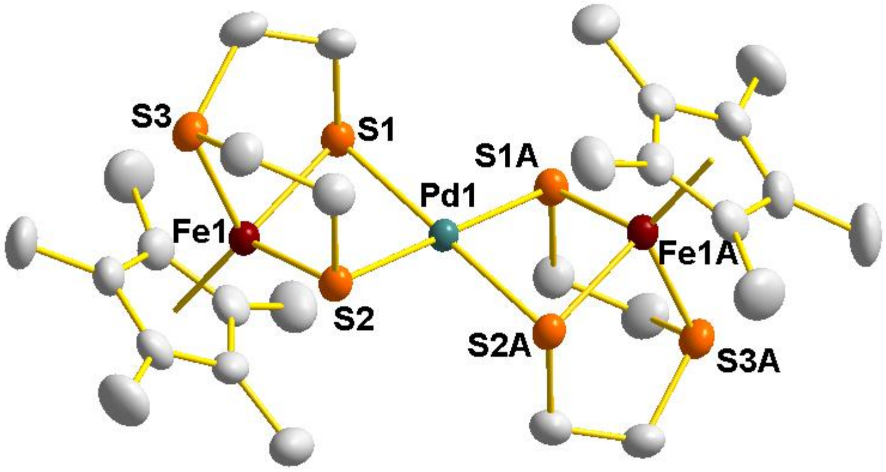

Table S6. Selected bond distances and angles for **C4**

Distances (Å)			
Fe1–Pd1	2.9945(10)	Pd1–S1	2.352(3)
Fe1–S1	2.234(3)	Pd1–S2	2.353(3)
Fe1–S2	2.230(3)	Fe1–Cp*1	1.7502(4)
Fe1–S3	2.251(2)		
Angles (°)			
S1–Fe1–S2	95.91(7)	S1–Pd1–S2	89.60(6)
S1–Fe1–S3	89.82(11)	S1–Pd1–S2A	179.97(14)
S2–Fe1–S3	89.63(11)	S1–Pd1–S1A	90.40(14)
Fe1–S1–Pd1	81.50(10)	S2–Pd1–S1A	179.97(12)
Fe1–S2–Pd1	81.54(10)	S2–Pd1–S2A	90.40(13)

Figure S8. ORTEP diagram of **C5**

Thermal ellipsoids are shown at a 50% probability level. All hydrogen atoms on carbons are omitted for clarity.

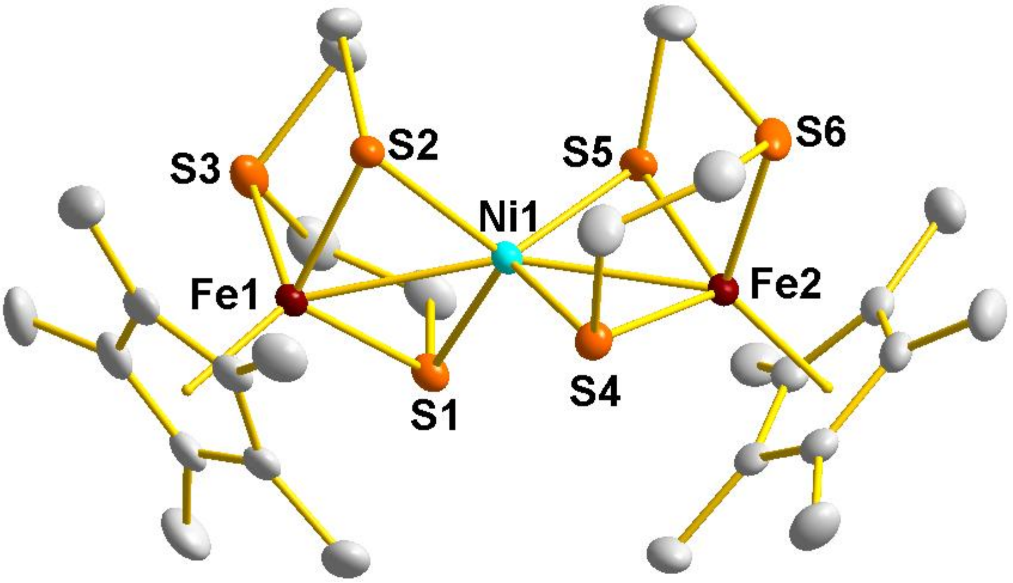

Table S7. Selected bond distances and angles for **C5**

Distances (Å)			
Fe1–Ni1	2.5599(17)	Ni1−S2	2.189(3)
Fe2–Ni1	2.5434(17)	Fe2–S4	2.213(3)
Fe1–S1	2.192(3)	Fe2–S5	2.215(3)
Fe1–S2	2.239(3)	Fe2–S6	2.228(3)
Fe1–S3	2.233(3)	Ni1–S4	2.239(2)
Ni1–S1	2.218(3)	Ni1–S5	2.183(3)
Angles (°)			
S1–Fe1–S2	106.87(10)	Fe2–S5–Ni1	70.67(8)
S1–Fe1–S3	89.56(10)	S1–Ni1–S2	107.67(10)
S2–Fe1–S3	88.11(10)	S1–Ni1–S4	117.76(10)
Fe1–S1–Ni1	70.97(8)	S1–Ni1–S5	102.52(10)
Fe1–S2–Ni1	70.62(8)	S4–Ni1–S5	107.55(10)
Fe2–S4–Ni1	69.67(8)	S4–Fe2–S5	107.33(10)

Figure S9. ORTEP diagram of **C6**

Thermal ellipsoids are shown at a 50% probability level. All hydrogen atoms on carbons are omitted for clarity.

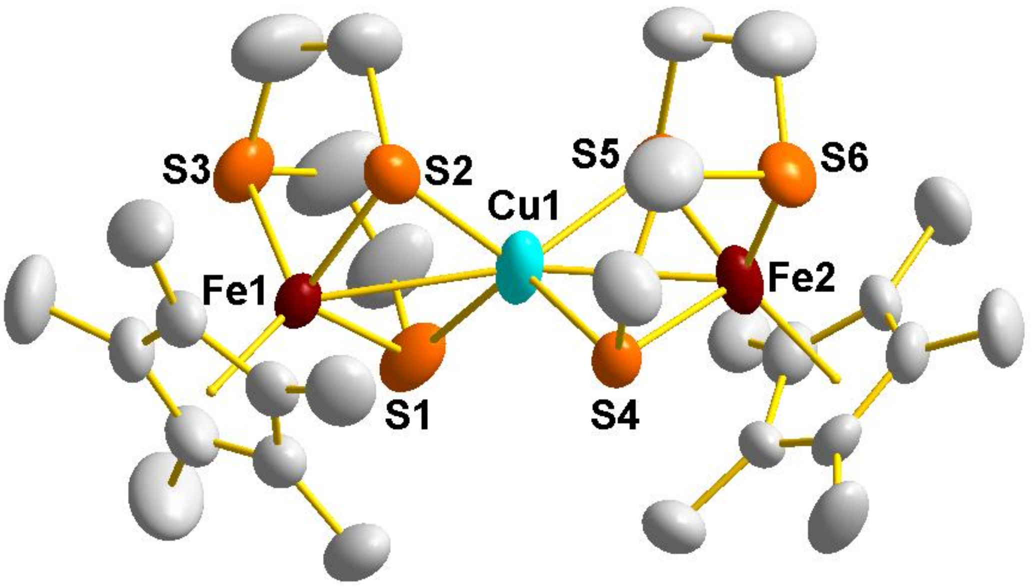

Table S8. Selected bond distances and angles for **C6**

Distances (Å)			
Fe1–Cu1	2.7658(10)	Cu1−S2	2.3048(17)
Fe2–Cu1	2.7465(10)	Fe2–S4	2.172(3)
Fe1–S1	2.2516(15)	Fe2–S5	2.2148(18)
Fe1–S2	2.2566(16)	Fe2–S6	2.110(3)
Fe1–S3	2.2268(16)	Cu1–S4	2.362(3)
Cu1–S1	2.302(2)	Cu1–S5	2.3048(17)
Angles (°)			
S1–Fe1–S2	104.62(7)	Fe2–S5–Cu1	74.82(5)
S1–Fe1–S3	88.51(6)	S1–Cu1–S2	101.62(6)
S2–Fe1–S3	90.66(16)	S1–Cu1–S4	122.32(11)
Fe1–S1–Cu1	74.79(6)	S1–Cu1–S5	112.06(8)
Fe1–S2–Cu1	74.72(6)	S4–Cu1–S5	102.00(9)
Fe2–S4–Cu1	75.16(10)	S4–Fe2–S5	108.43(9)

Figure S10. ORTEP diagram of **N1**

Thermal ellipsoids are shown at a 50% probability level. All hydrogen atoms on carbons are omitted for clarity.

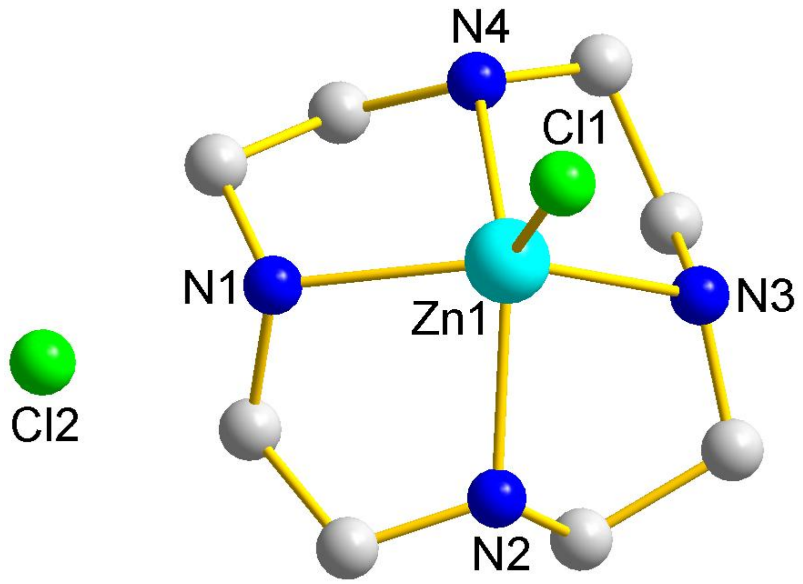

Table S9. Selected bond distances and angles for **N1**

Distances (Å)			
Zn1–N1	2.153(3)	Zn1–N4	2.153(3)
Zn1–N2	2.155(3)	Zn1–Cl1	2.2402(5)
Zn1–N3	2.153(3)		
Angles (°)			
N1–Zn1–N2	80.85(13)	N1–Zn1–Cl1	113.16(8)
N1–Zn1–N3	133.47(11)	N2–Zn1–Cl1	113.23(8)
N1–Zn1–N4	81.04(13)	N3–Zn1–Cl1	113.18(8)
N2–Zn1–N3	80.94(13)	N4–Zn1–Cl1	113.25(8)
N3–Zn1–N4	81.08(13)		

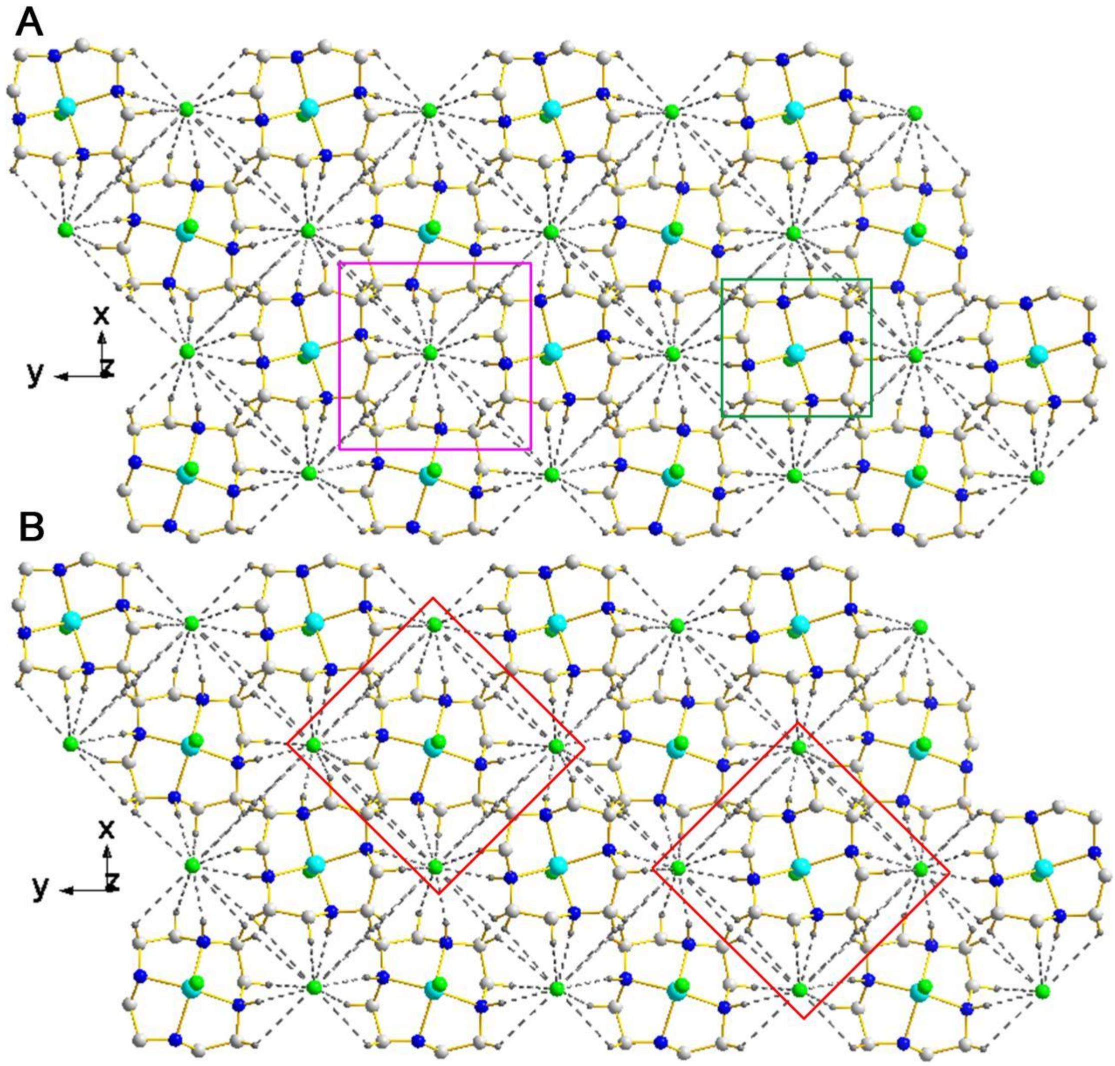

Figure S11. Crystal packing maps of **N1** (CCDC 2171267) along z-axis, containing 12-element ring **N1** complexes (A, green box), 16-element ring centered on the free chlorine atoms (A, pink box), and regular arrangement (B, red box) of square cells formed by hydrogen bonds.

Figure S12. ORTEP diagram of **N2**

Thermal ellipsoids are shown at a 50% probability level. All hydrogen atoms on carbons are omitted for clarity.

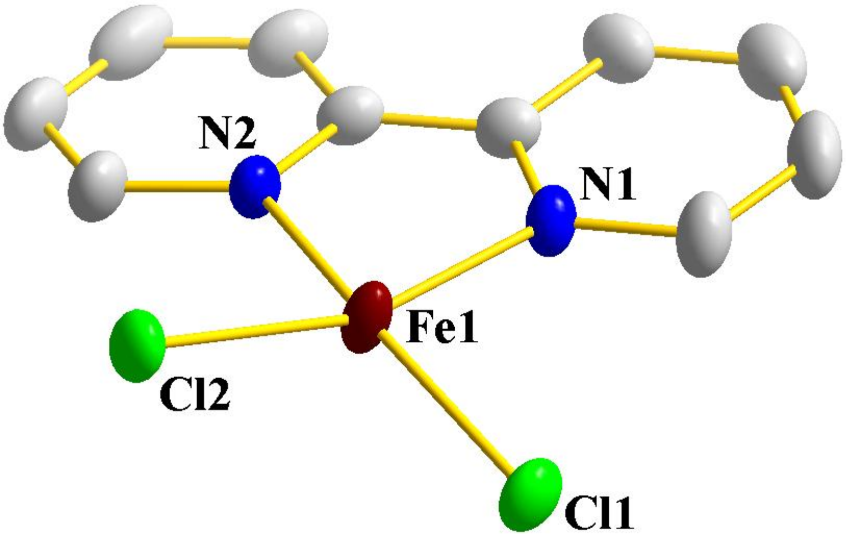

Table S10. Selected bond distances and angles for **N2**

Distances (Å)			
Fe1–N1	2.1674(16)	Fe1–Cl1	2.4178(9)
Fe1–N2	2.1674(16)	Fe1–Cl2	2.6706(13)
Angles (°)			
N1–Fe1–N2	74.95(11)	N2–Fe1–Cl1	162.83(7)
N1–Fe1–Cl1	94.15(6)	N2–Fe1–Cl2	84.29(8)
N1–Fe1–Cl2	162.83(7)	Cl1–Fe1–Cl2	83.46(4)

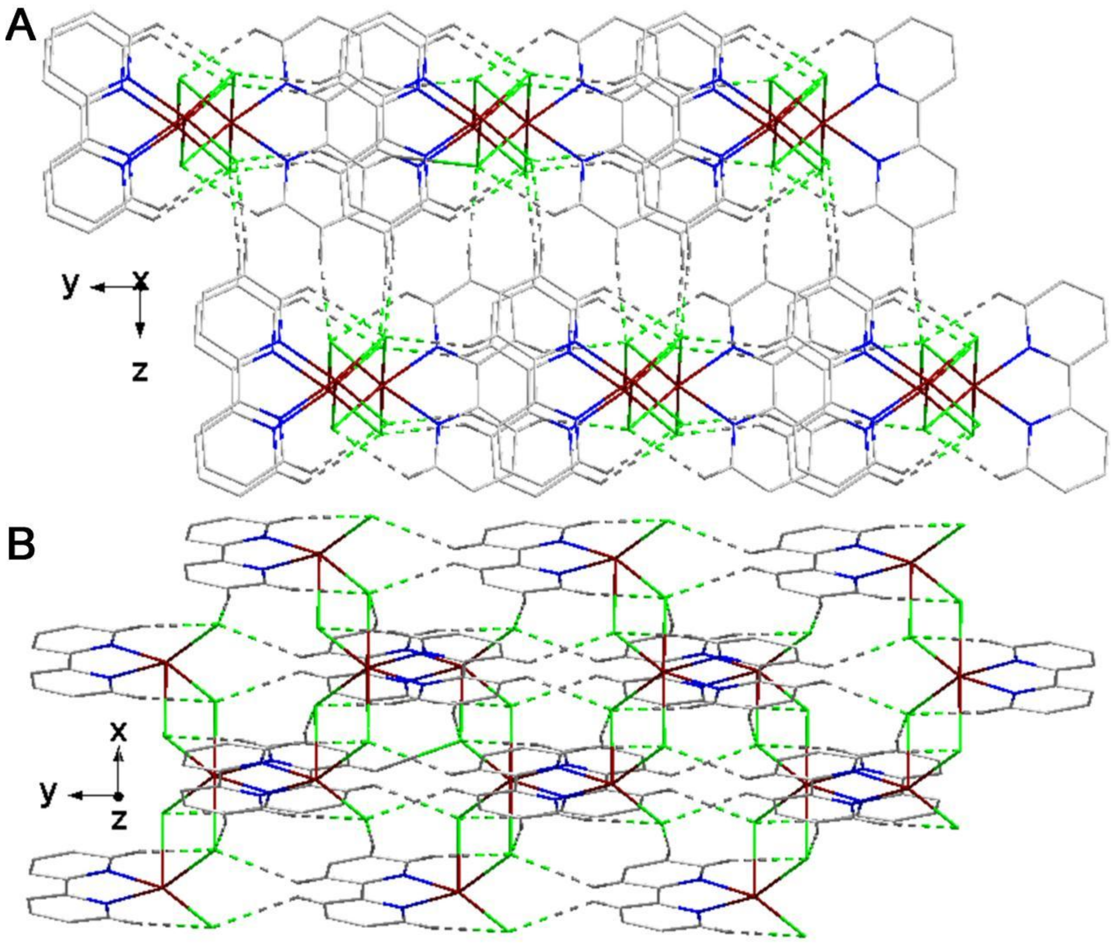

Figure S13. Crystal packing maps of **N2** (CCDC 2178630) along x- (A) and z-axes (B).

Figure S14. ORTEP diagram of **N3**

Thermal ellipsoids are shown at a 50% probability level. All hydrogen atoms on carbons are omitted for clarity.

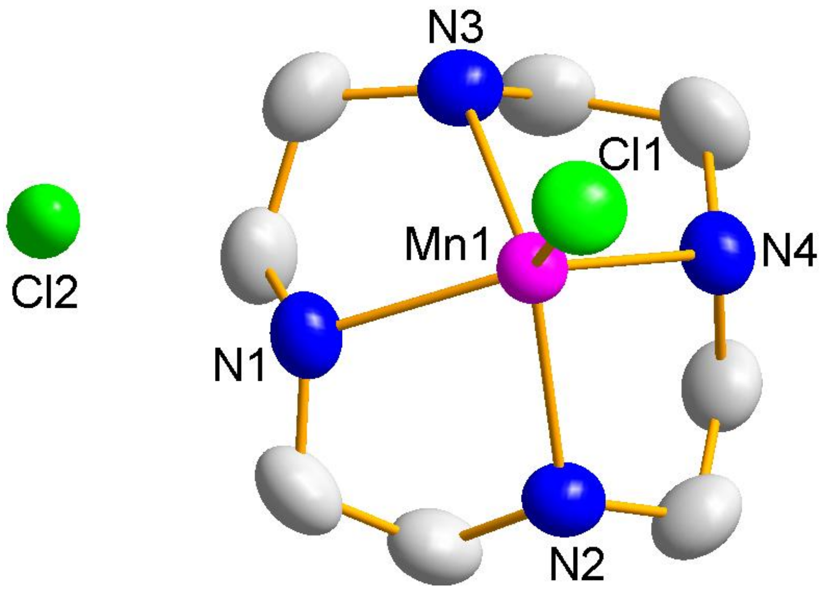

Table S11. Selected bond distances and angles for **N3**

Distances (Å)			
Mn1–N1	2.230(3)	Mn1–N4	2.234(3)
Mn1–N2	2.231(3)	Mn1–Cl1	2.3089(4)
Mn1–N3	2.236(3)		
Angles (°)			
N1–Mn1–N2	78.51(12)	N1–Mn1–Cl1	116.41(7)
N1–Mn1–N3	78.47(12)	N2–Mn1–Cl1	116.43(7)
N1–Mn1–N4	127.09(10)	N3–Mn1–Cl1	116.48(7)
N2–Mn1–N3	127.14(10)	N4–Mn1–Cl1	116.45(7)
N3–Mn1–N4	78.60(12)		

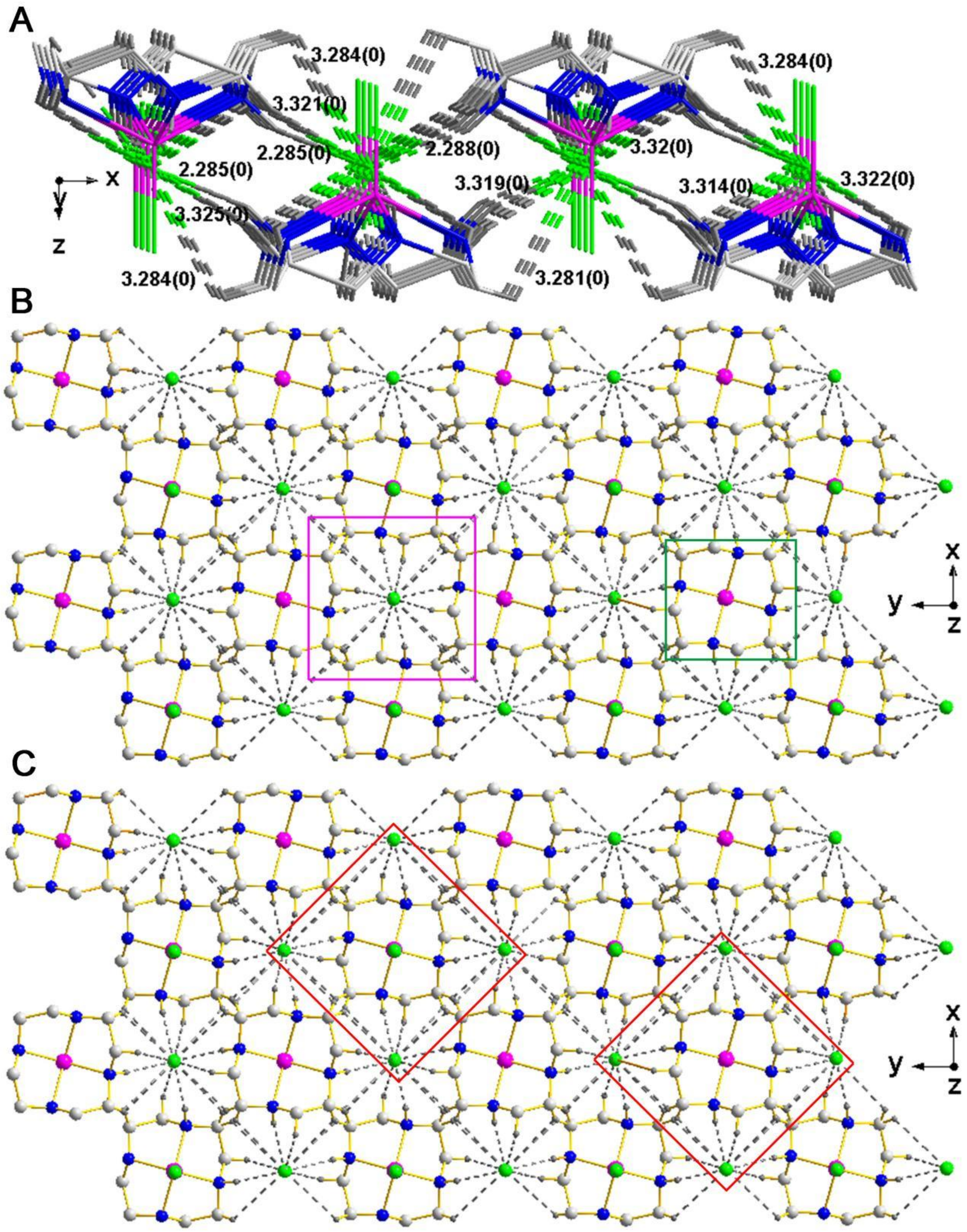

Figure S15. Crystal packing maps of **N3** (CCDC 2178629) along y- and z-axes, containing 12-element ring **N3** complexes (A, green box), 16-element ring centered on the free chlorine atoms (A, pink box), and regular arrangement (B, red box) of square cells formed by hydrogen bonds.

IV. NMR Spectra

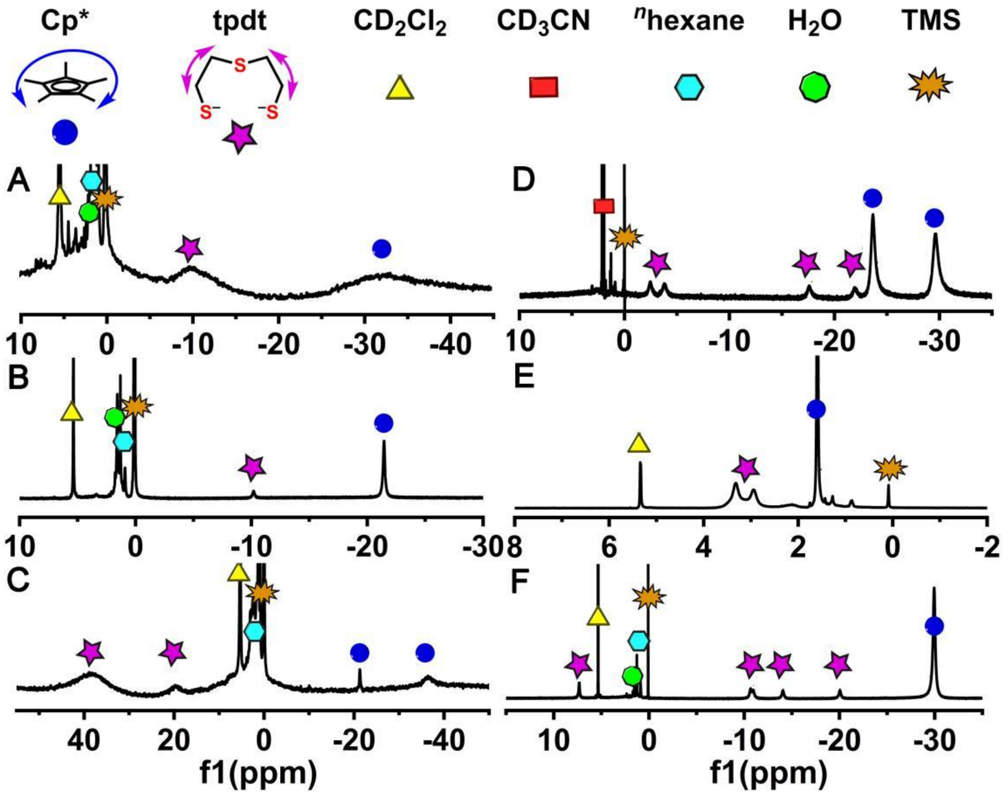

Figure S16. ^{1}H NMR spectra of **C1−C6** in CD_2Cl_2 (A, **C1**; B, **C2**; C, **C3**; E, **C5**; F, **C6**) or CD_3CN (D, **C4**).

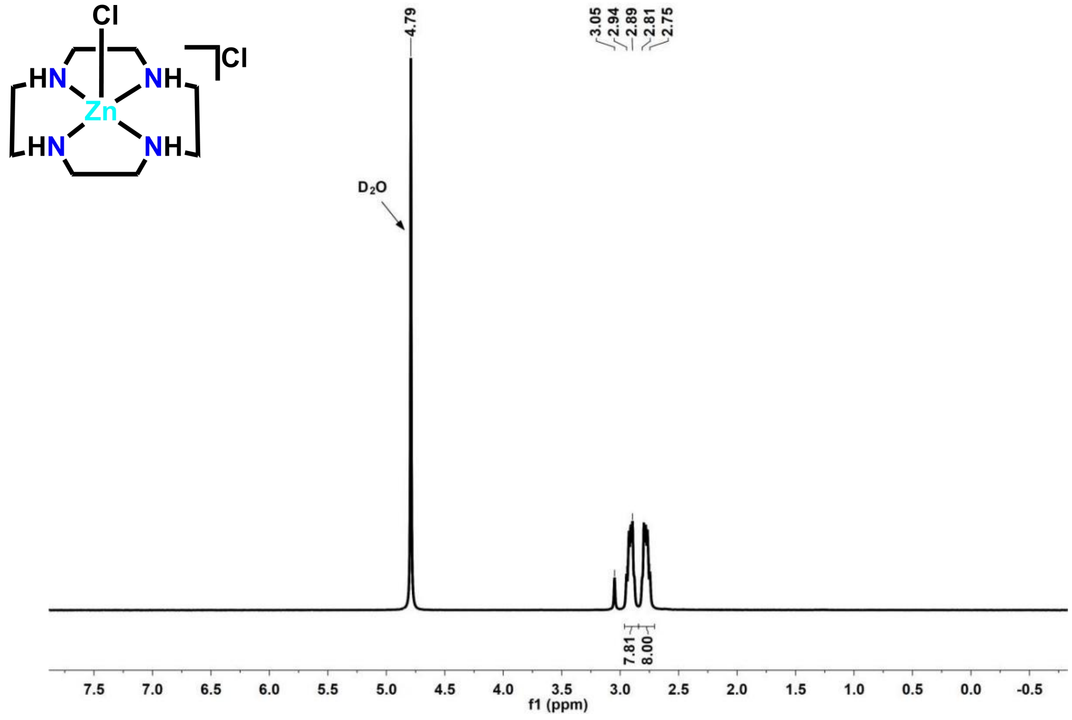

Figure S17. ^{1}H NMR spectrum of **N1** in D_2O.

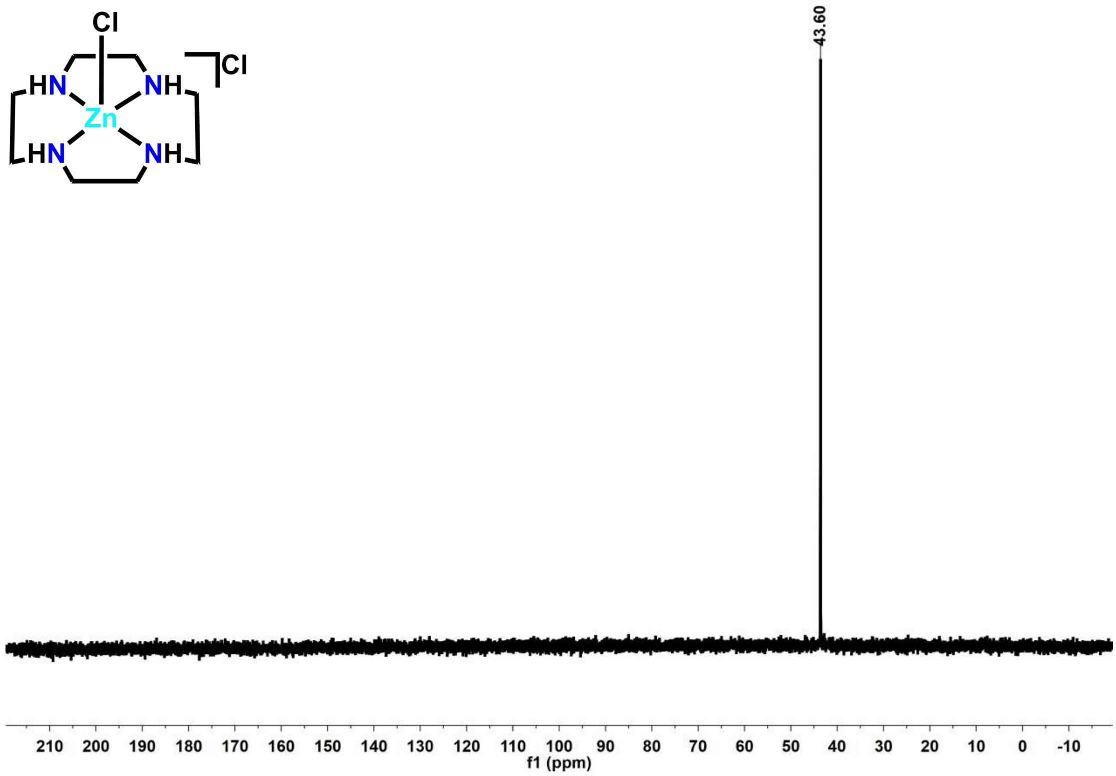

Figure S18. ^{13}C NMR spectrum of **N1** in D_2O.

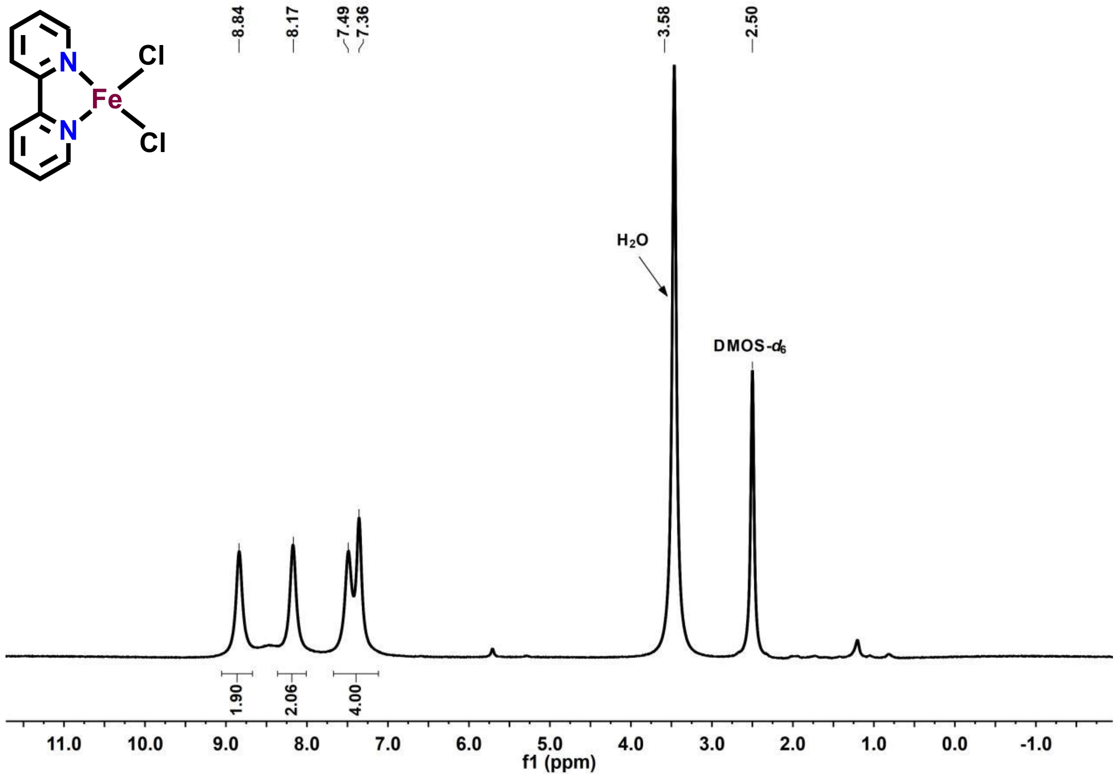

Figure S19. ^{1}H NMR spectrum of **N2** in DMSO-d_6.

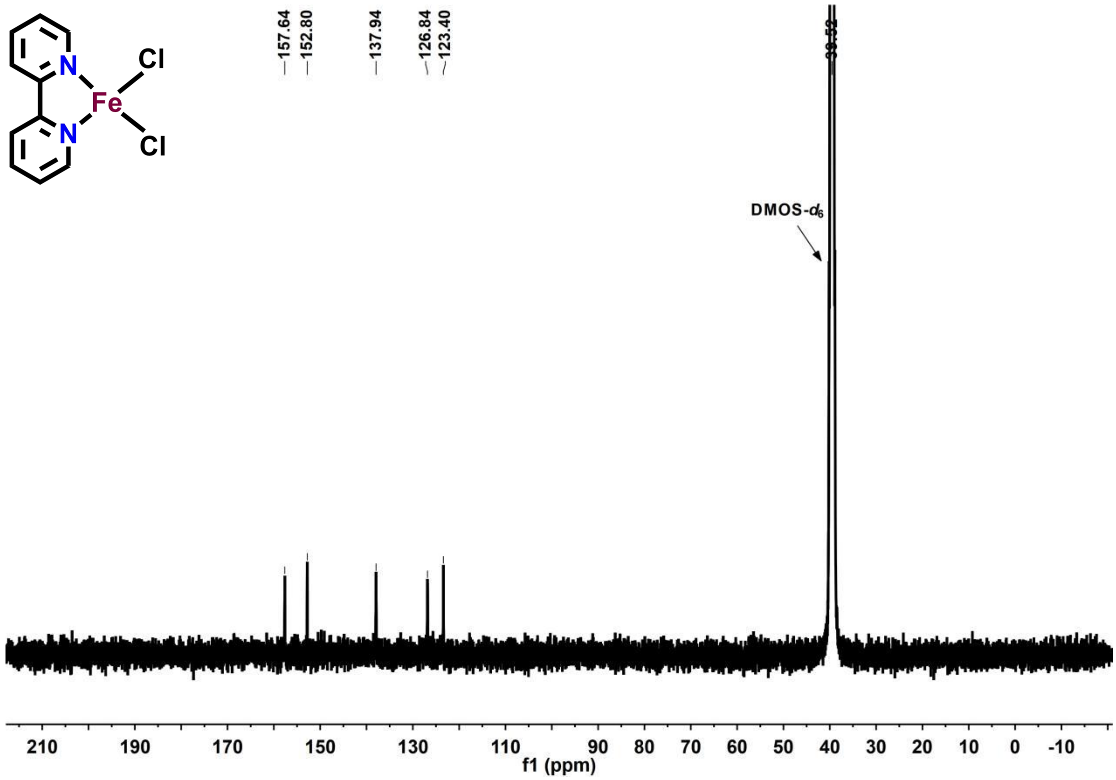

Figure S20. ^{13}C NMR spectrum of **N2** in DMSO-d_6.

V. EPR Spectra

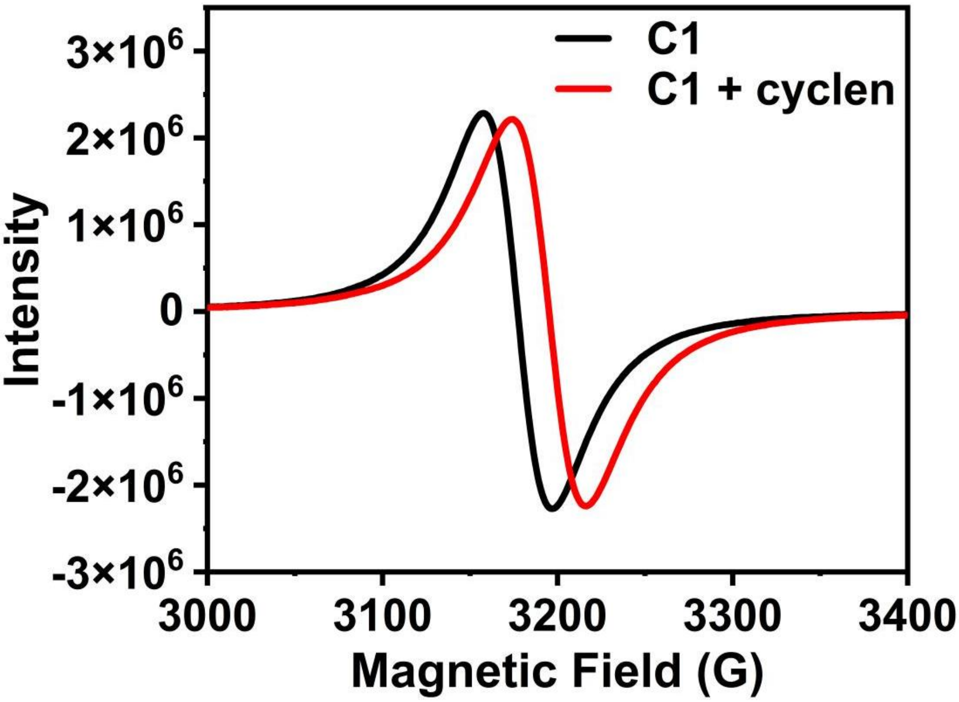

Figure S21. EPR spectrum of **C1** before and after the addition of cyclen in CH_2Cl_2.

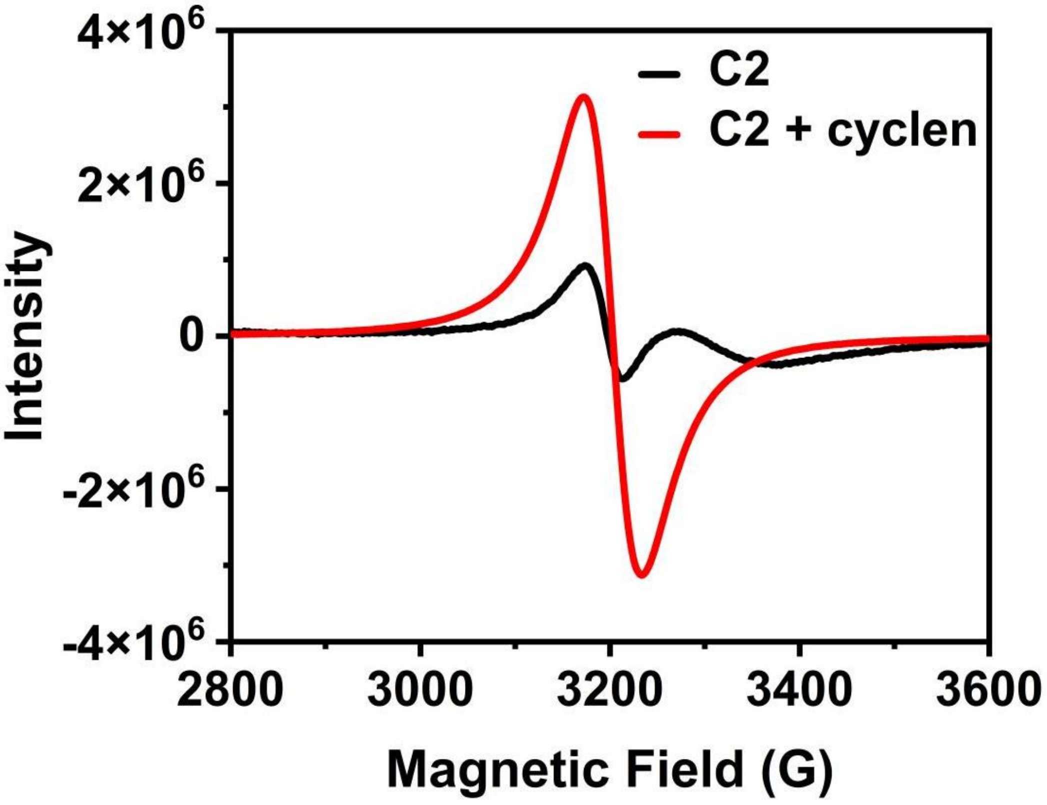

Figure S22. EPR spectrum of **C2** before and after the addition of cyclen in CH_2Cl_2.

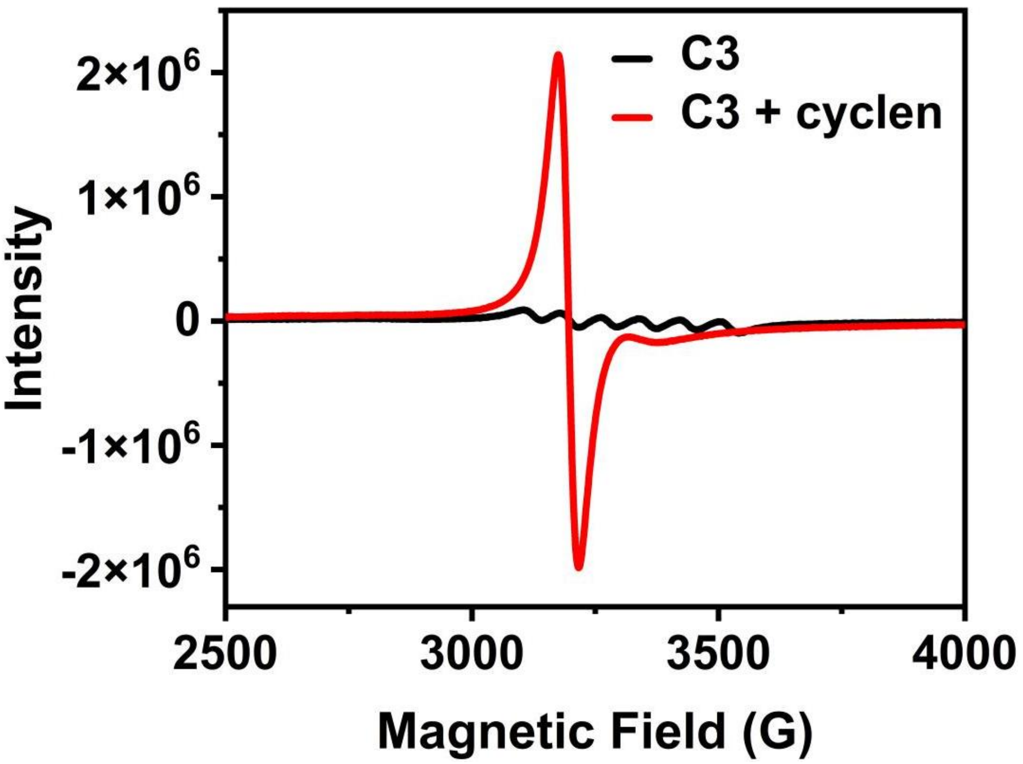

Figure S23. EPR spectrum of **C3** before and after the addition of cyclen in CH_2Cl_2.

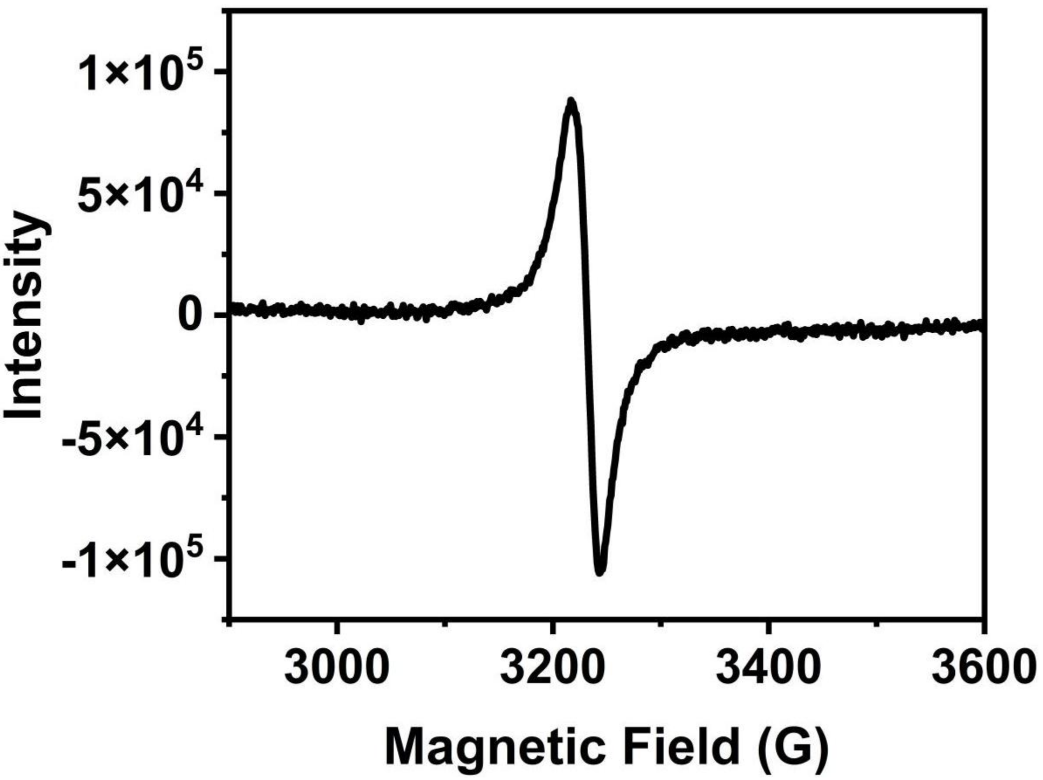

Figure S24. EPR spectrum of **C4** in CH_3CN.

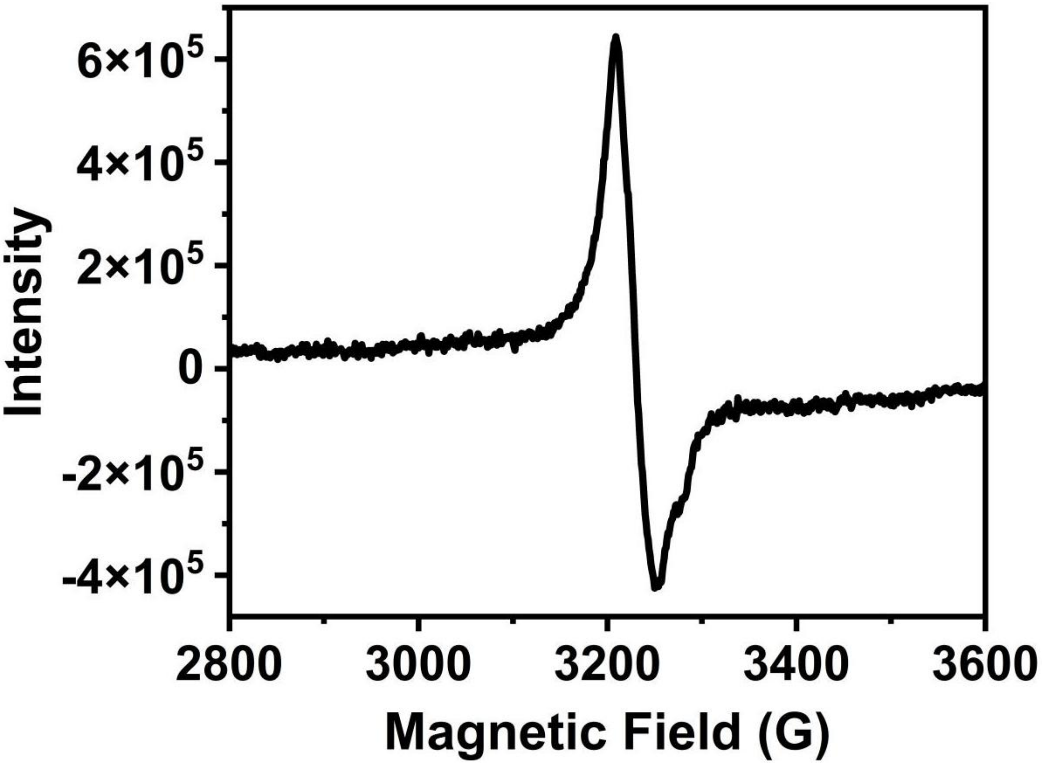

Figure S25. EPR spectrum of **C6** in CH_2Cl_2.

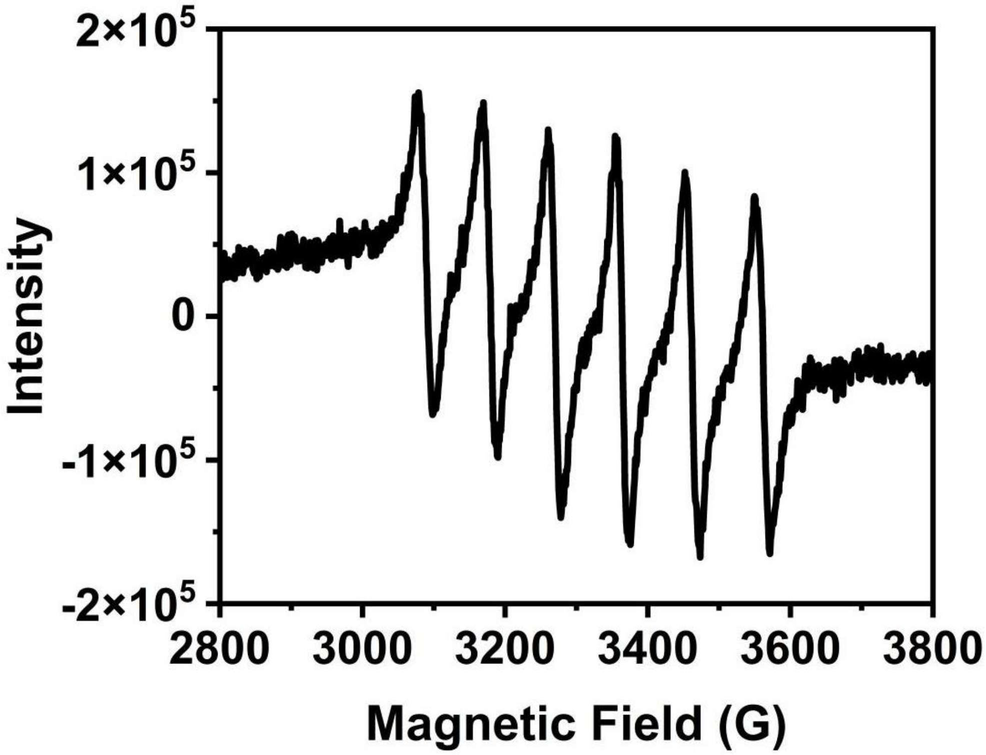

Figure S26. EPR spectrum of **N3** in CH_3OH.

VI. UV-vis Spectra

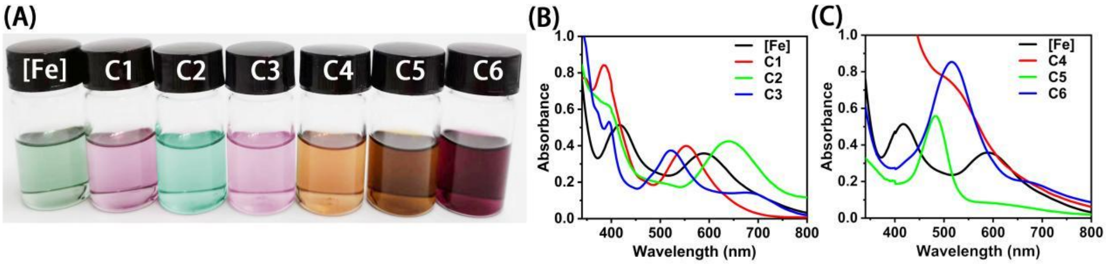

Figure S27. (A) Photos of [Cp*Fe(η^3-tpdt)] and **C1−C6** in CH_2Cl_2. [Cp*Fe(η^3-tpdt)] is abbreviated as [Fe]. (B, C) UV-vis spectra of **C1−C3** (B) and **C4−C6** (C) compared to mononuclear iron precursor at the concentration of 0.33 mM in CH_2Cl_2 at 20 °C.

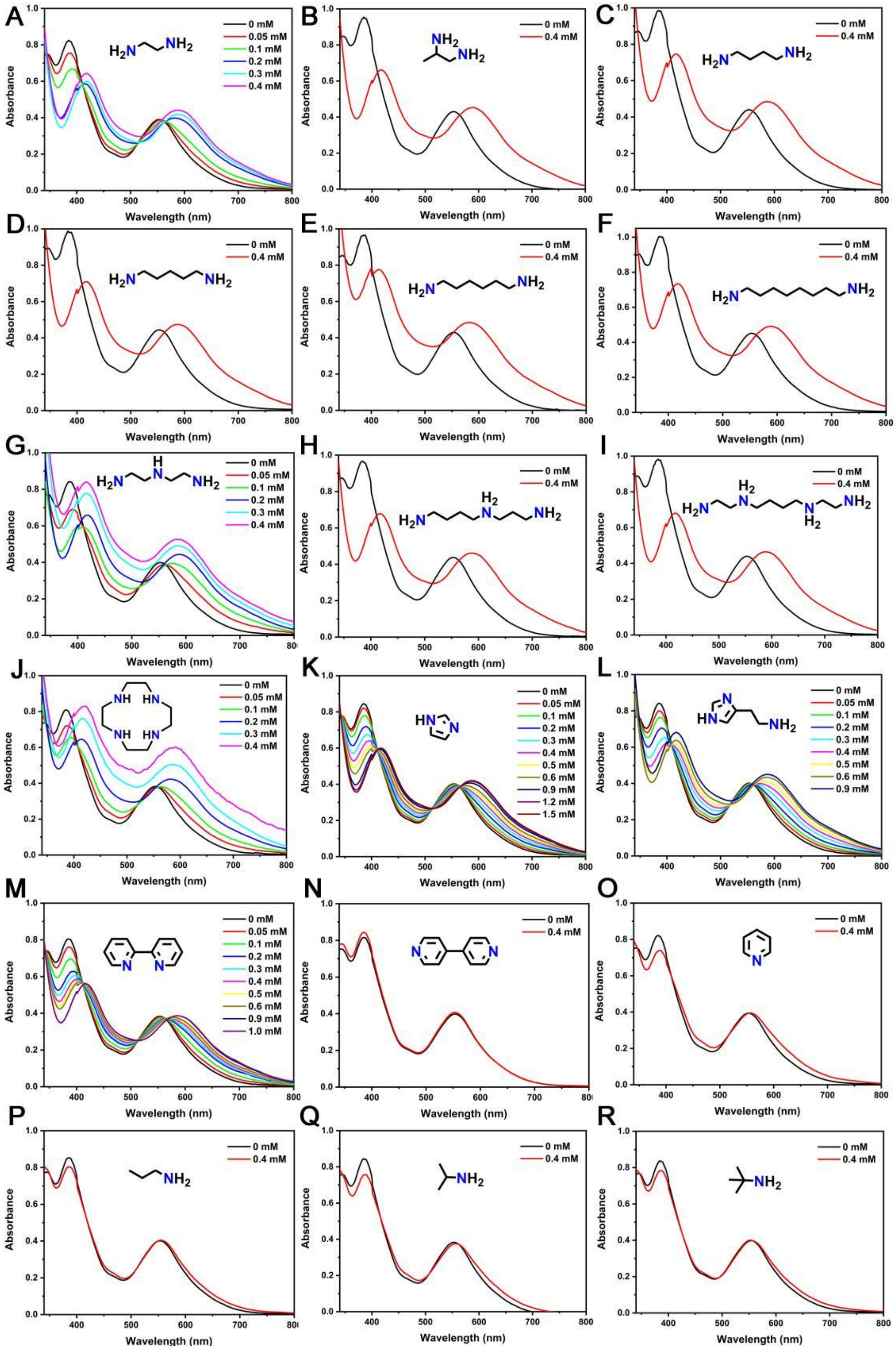

Figure S28. UV-vis spectra of **C1** (0.33 mM) before and after the addition of different concentrations of ethylenediamine (A), 1,2-diaminopropane (B), 1,4-diaminobutane (C), 1,5-diaminopentane (D), hexamethylenediamine (E), 1,8-diaminooctane (F), diethylenetriamine (G), spermidine (H), spermine (I), cyclen (J), imidazole (K), histamine (L), 2,2'-bipyrindine (M), 4,4'-bipyridine (N), pyridine (O), ^{n}propylpamine (P), isopropylamine (Q), and tert-butylamine (R).

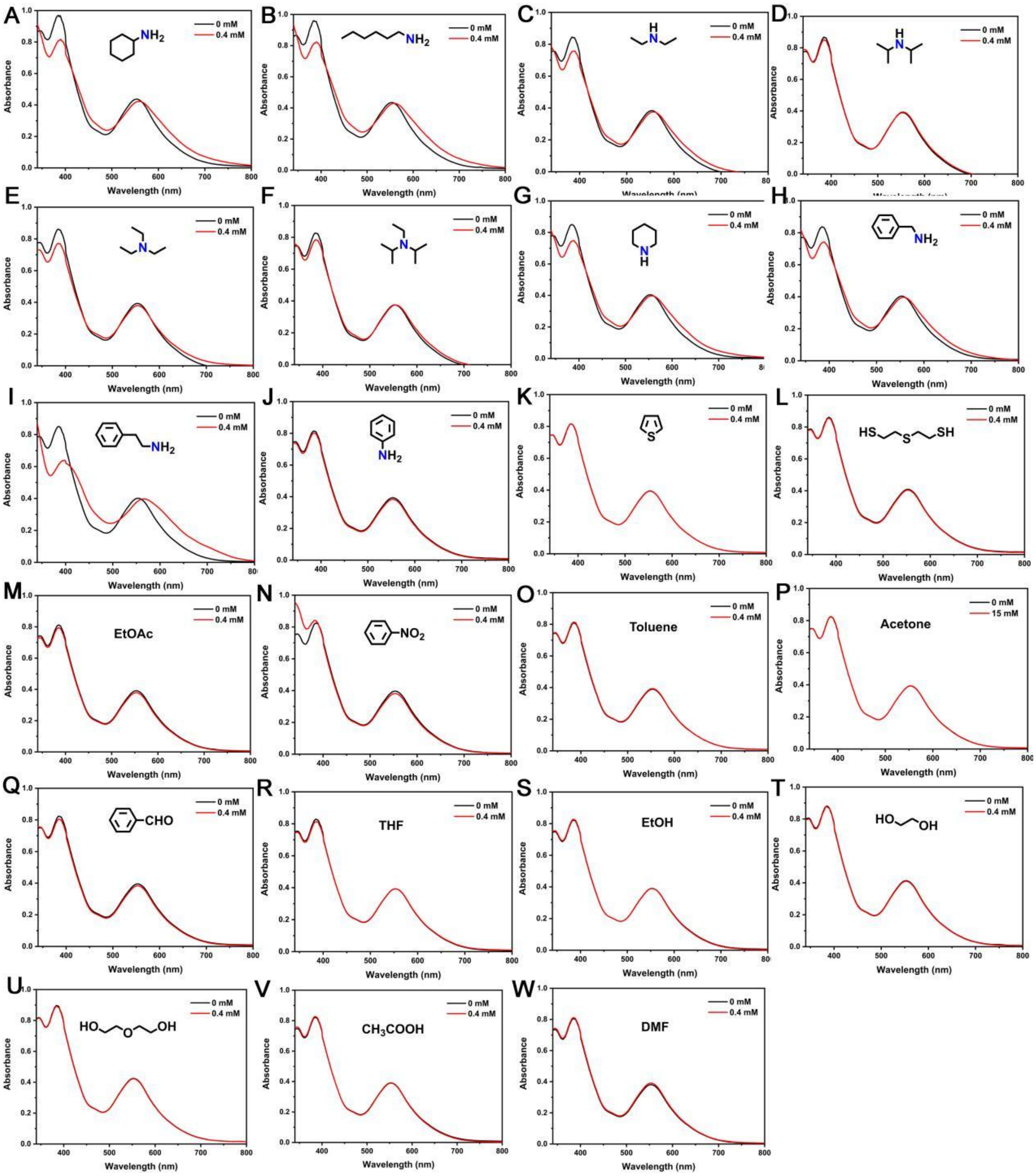

Figure S29. UV-vis spectra of **C1** (0.33 mM) before and after the addition of cyclohexylamine (A), hexylamine (B), diethylamine (C), diisopropylamine (D), triethylamine (E), *N,N*-diisopropylethylamine (F), piperidine (G), benzylamine (H), and phenethylamine (I), aniline (J), thiophene (K), 3-thiapentane-1,5-dithiolate (L), EtOAc (M), nitrobenzene (N), toluene (O), acetone (P), benzaldehyde (Q), THF (R), EtOH (S), ethylene glycol (T), diethylene glycol (U), CH_3COOH (V), and DMF (W) at the concentration of 0.40 mM.

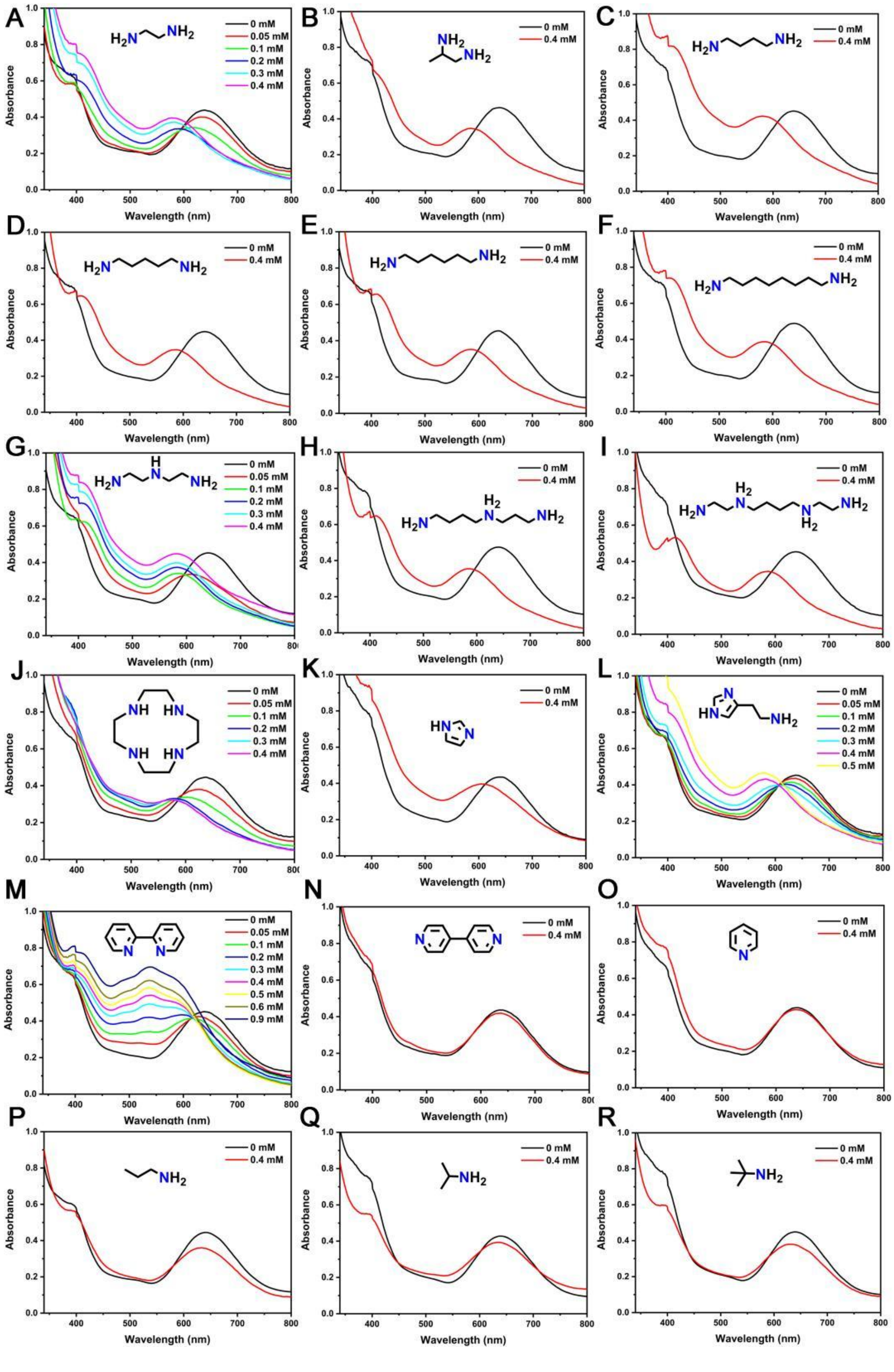

Figure S30. UV-vis spectra of **C2** (0.33 mM) before and after the addition of different concentrations of ethylenediamine (A), 1,2-diaminopropane (B), 1,4-diaminobutane (C), 1,5-diaminopentane (D), hexamethylenediamine (E), 1,8-diaminooctane (F), diethylenetriamine (G), spermidine (H), spermine (I), cyclen (J), imidazole (K), histamine (L), 2,2'-bipyrindine (M), 4,4'-bipyridine (N), pyridine (O), ^{n}propylpamine (P), isopropylamine (Q), and tert-butylamine (R).

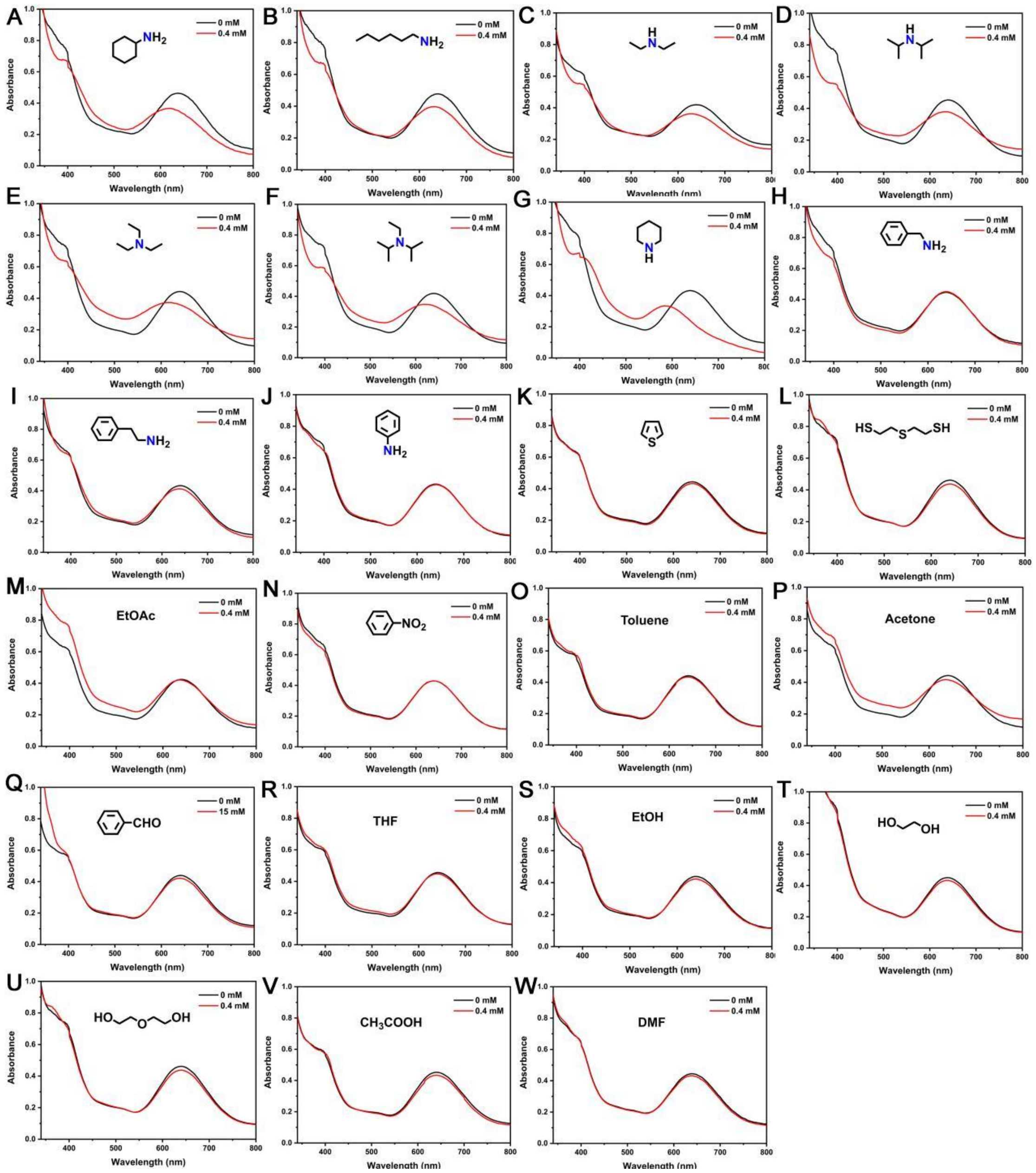

Figure S31. UV-vis spectra of **C2** (0.33 mM) before and after the addition of cyclohexylamine (A), hexylamine (B), diethylamine (C), diisopropylamine (D), triethylamine (E), *N,N*-diisopropylethylamine (F), piperidine (G), benzylamine (H), and phenethylamine (I), aniline (J), thiophene (K), 3-thiapentane-1,5-dithiolate (L), EtOAc (M), nitrobenzene (N), toluene (O), acetone (P), benzaldehyde (Q), THF (R), EtOH (S), ethylene glycol (T), diethylene glycol (U), CH_3COOH (V), and DMF (W) at the concentration of 0.40 mM.

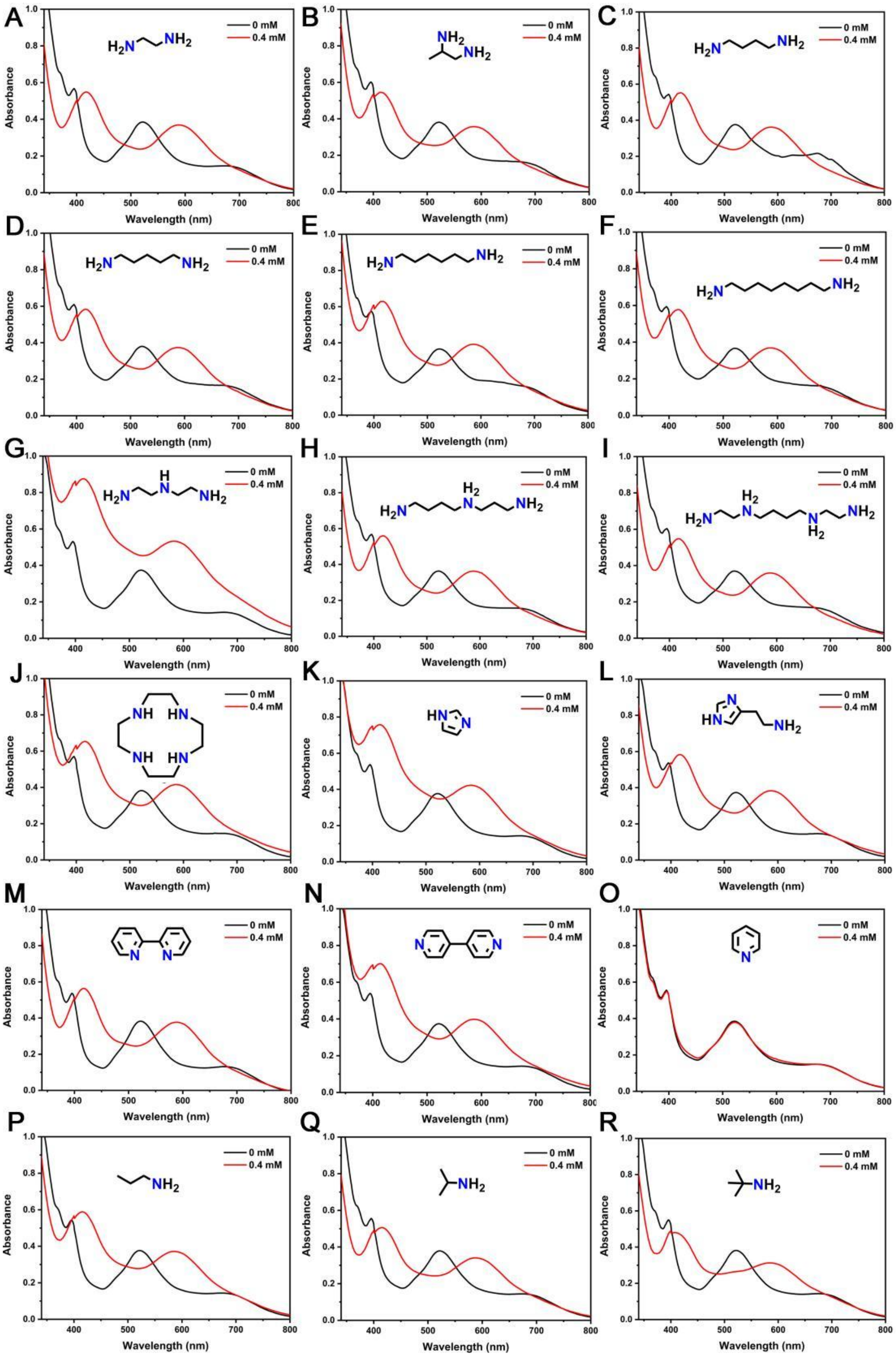

Figure S32. UV-vis spectra of **C3** (0.33 mM) before and after the addition of ethylenediamine (A), 1,2-diaminopropane (B), 1,4-diaminobutane (C), 1,5-diaminopentane (D), hexamethylenediamine (E), 1,8-diaminooctane (F), diethylenetriamine (G), spermidine (H), spermine (I), cyclen (J), imidazole (K), histamine (L), 2,2'-bipyrindine (M), 4,4'-bipyridine (N), pyridine (O), ^{n}propylpamine (P), isopropylamine (Q), and tert-butylamine (R).

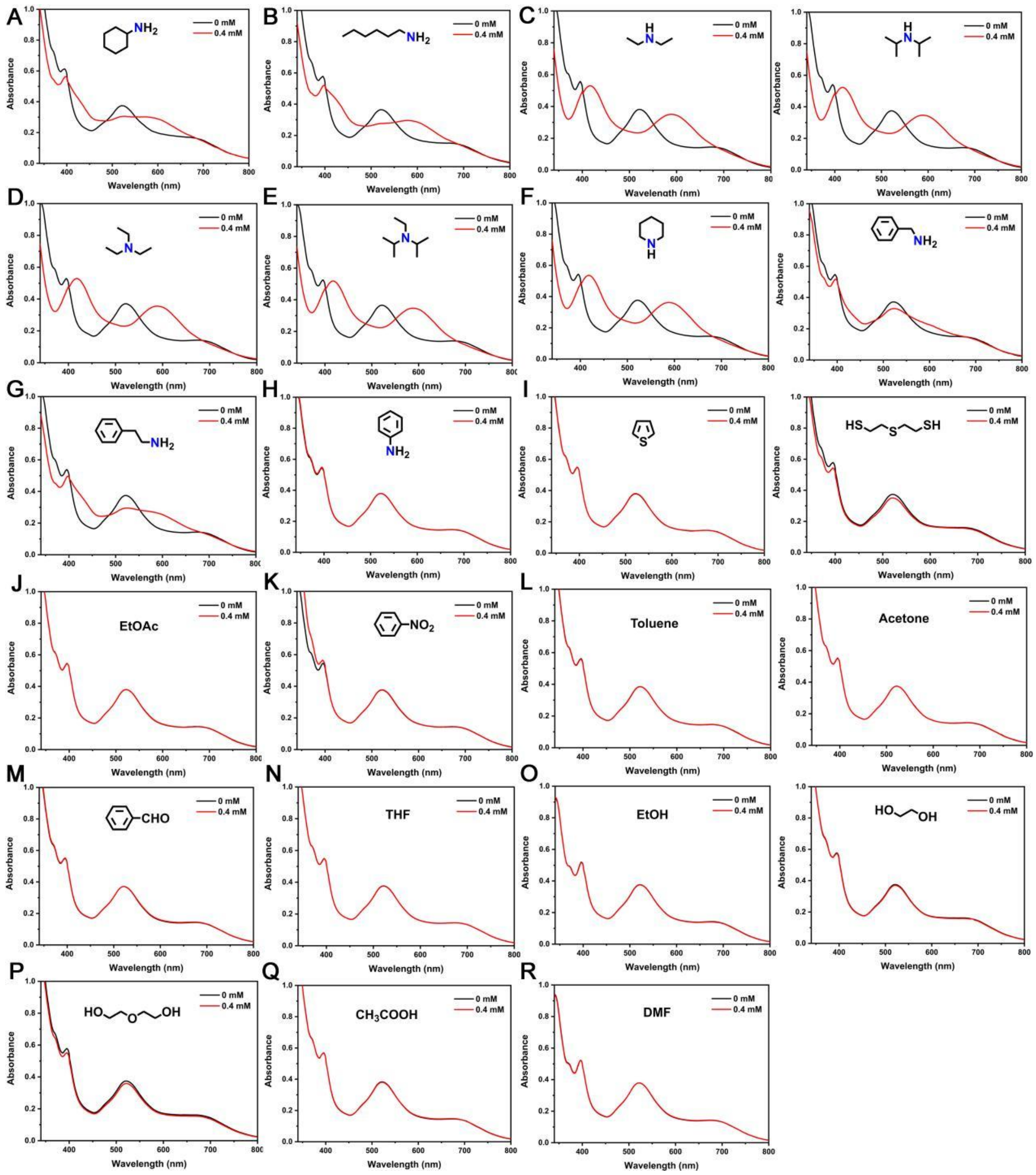

Figure S33. UV-vis spectra of **C3** (0.33 mM) before and after the addition of cyclohexylamine (A), hexylamine (B), diethylamine (C), diisopropylamine (D), triethylamine (E), *N,N*-diisopropylethylamine (F), piperidine (G), benzylamine (H), and phenethylamine (I), aniline (J), thiophene (K), 3-thiapentane-1,5-dithiolate (L), EtOAc (M), nitrobenzene (N), toluene (O), acetone (P), benzaldehyde (Q), THF (R), EtOH (S), ethylene glycol (T), diethylene glycol (U), CH_3COOH (V), and DMF (W) at the concentration of 0.40 mM.

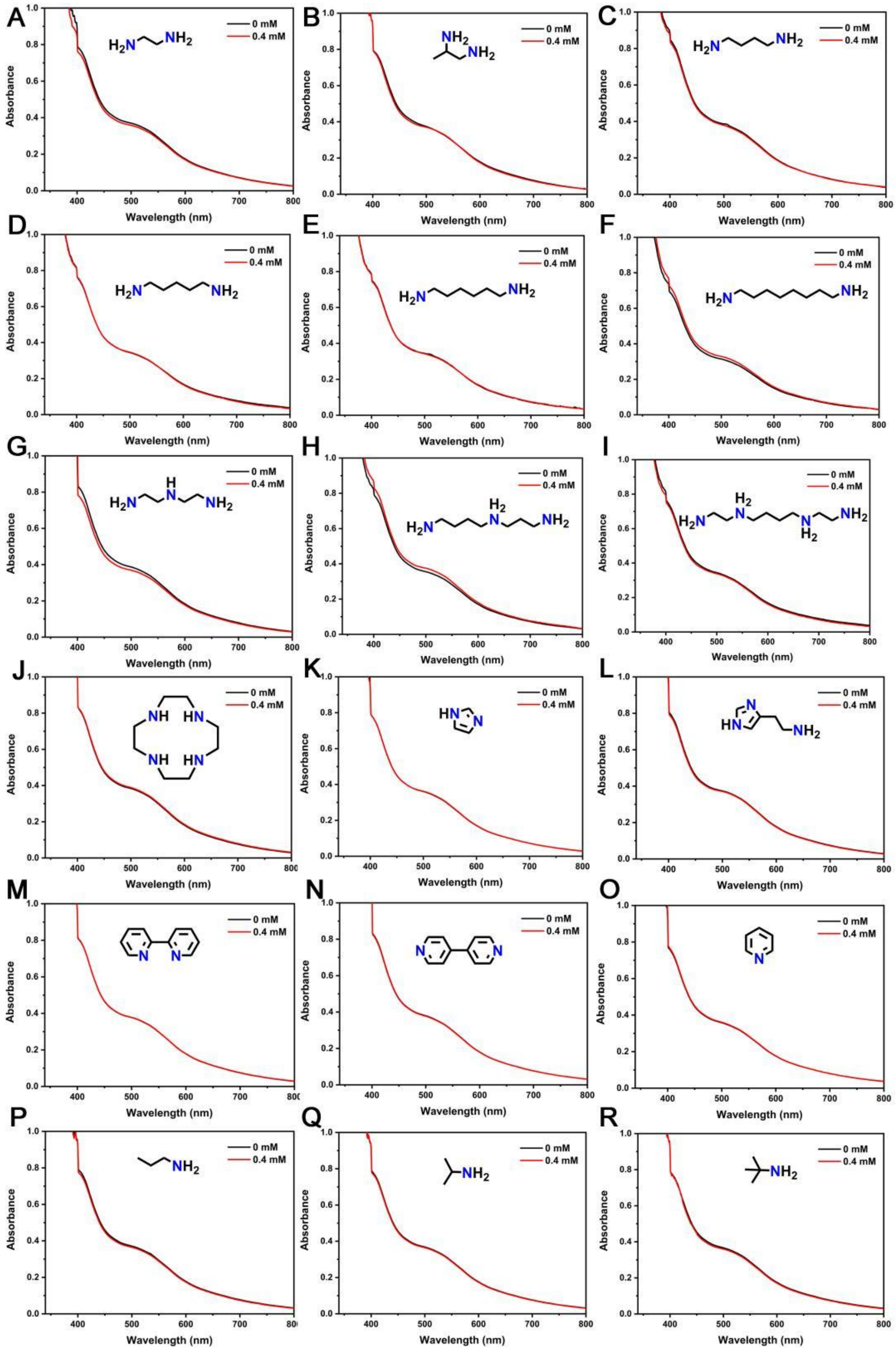

Figure S34. UV-vis spectra of **C4** (0.33 mM) before and after the addition of ethylenediamine (A), 1,2-diaminopropane (B), 1,4-diaminobutane (C), 1,5-diaminopentane (D), hexamethylenediamine (E), 1,8-diaminooctane (F), diethylenetriamine (G), spermidine (H), spermine (I), cyclen (J), imidazole (K), histamine (L), 2,2'-bipyrindine (M), 4,4'-bipyridine (N), pyridine (O), ^{n}propylpamine (P), isopropylamine (Q), and tert-butylamine (R).

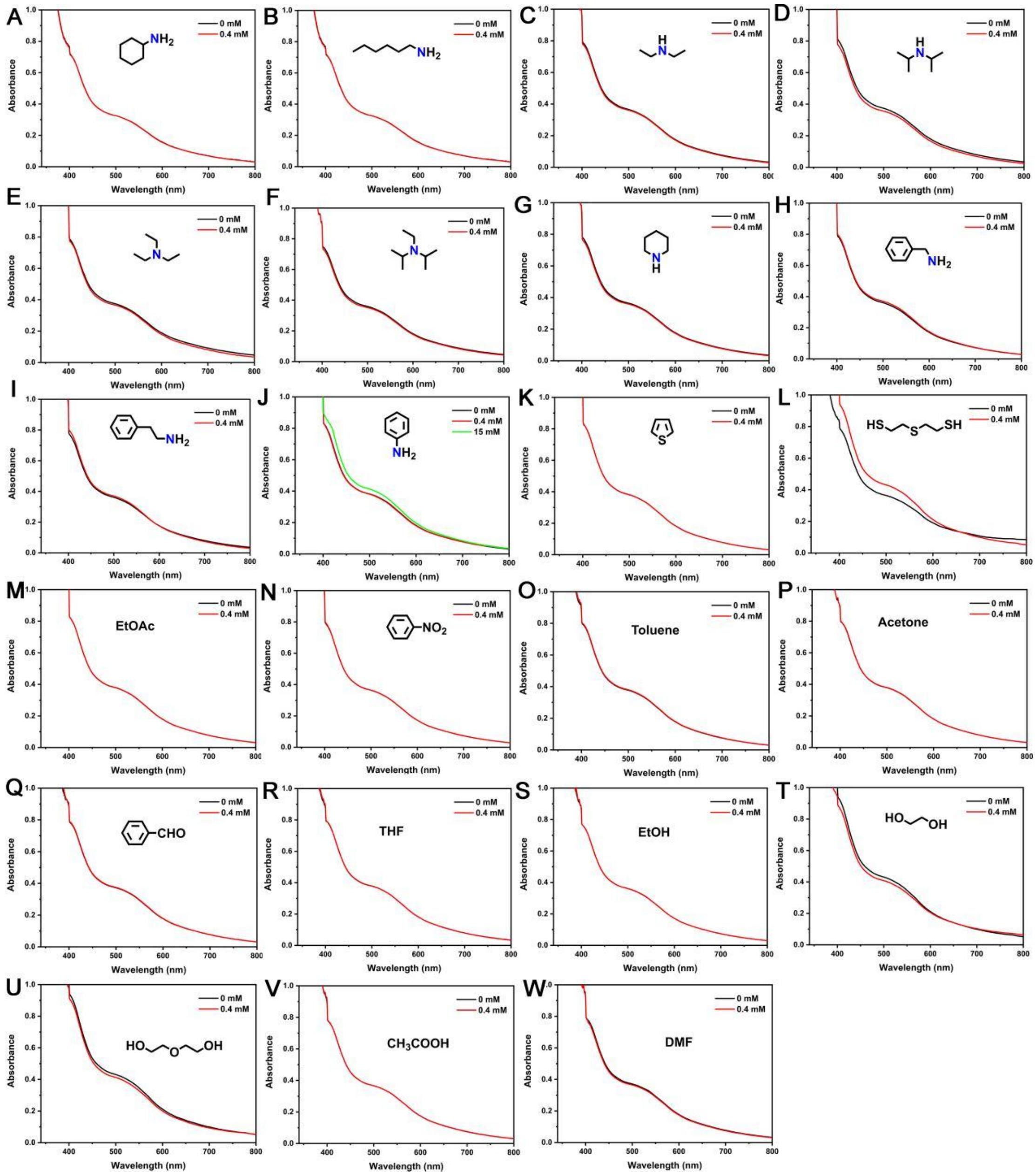

Figure S35. UV-vis spectra of **C4** (0.33 mM) before and after the addition of cyclohexylamine (A), hexylamine (B), diethylamine (C), diisopropylamine (D), triethylamine (E), *N,N*-diisopropylethylamine (F), piperidine (G), benzylamine (H), and phenethylamine (I), aniline (J), thiophene (K), 3-thiapentane-1,5-dithiolate (L), EtOAc (M), nitrobenzene (N), toluene (O), acetone (P), benzaldehyde (Q), THF (R), EtOH (S), ethylene glycol (T), diethylene glycol (U), CH_3COOH (V), and DMF (W) at the concentration of 0.40 mM.

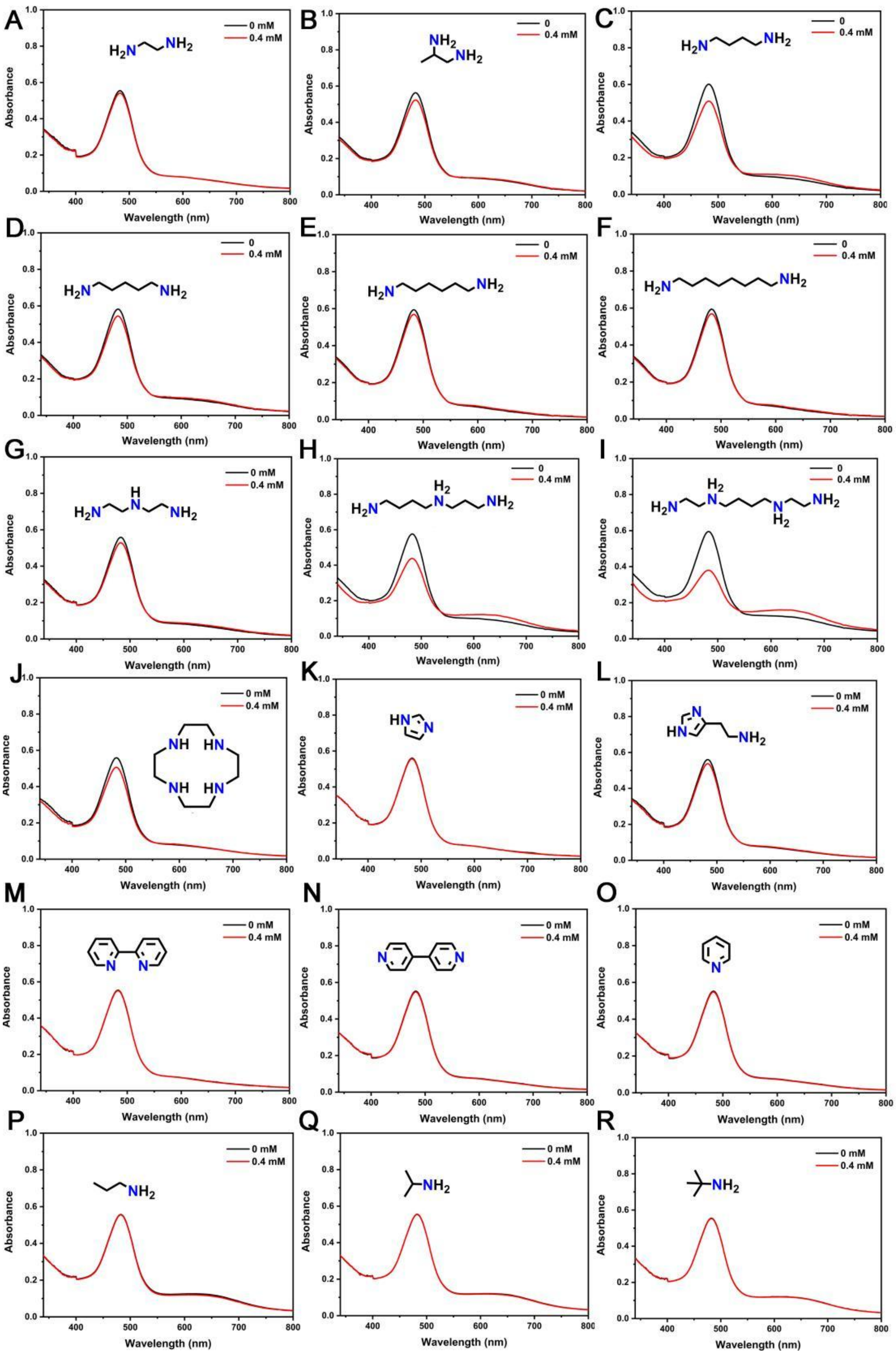

Figure S36. UV-vis spectra of **C5** (0.33 mM) before and after the addition of ethylenediamine (A), 1,2-diaminopropane (B), 1,4-diaminobutane (C), 1,5-diaminopentane (D), hexamethylenediamine (E), 1,8-diaminooctane (F), diethylenetriamine (G), spermidine (H), spermine (I), cyclen (J), imidazole (K), histamine (L), 2,2’-bipyrindine (M), 4,4’-bipyridine (N), pyridine (O), ^{n}propylpamine (P), isopropylamine (Q), and tert-butylamine (R).

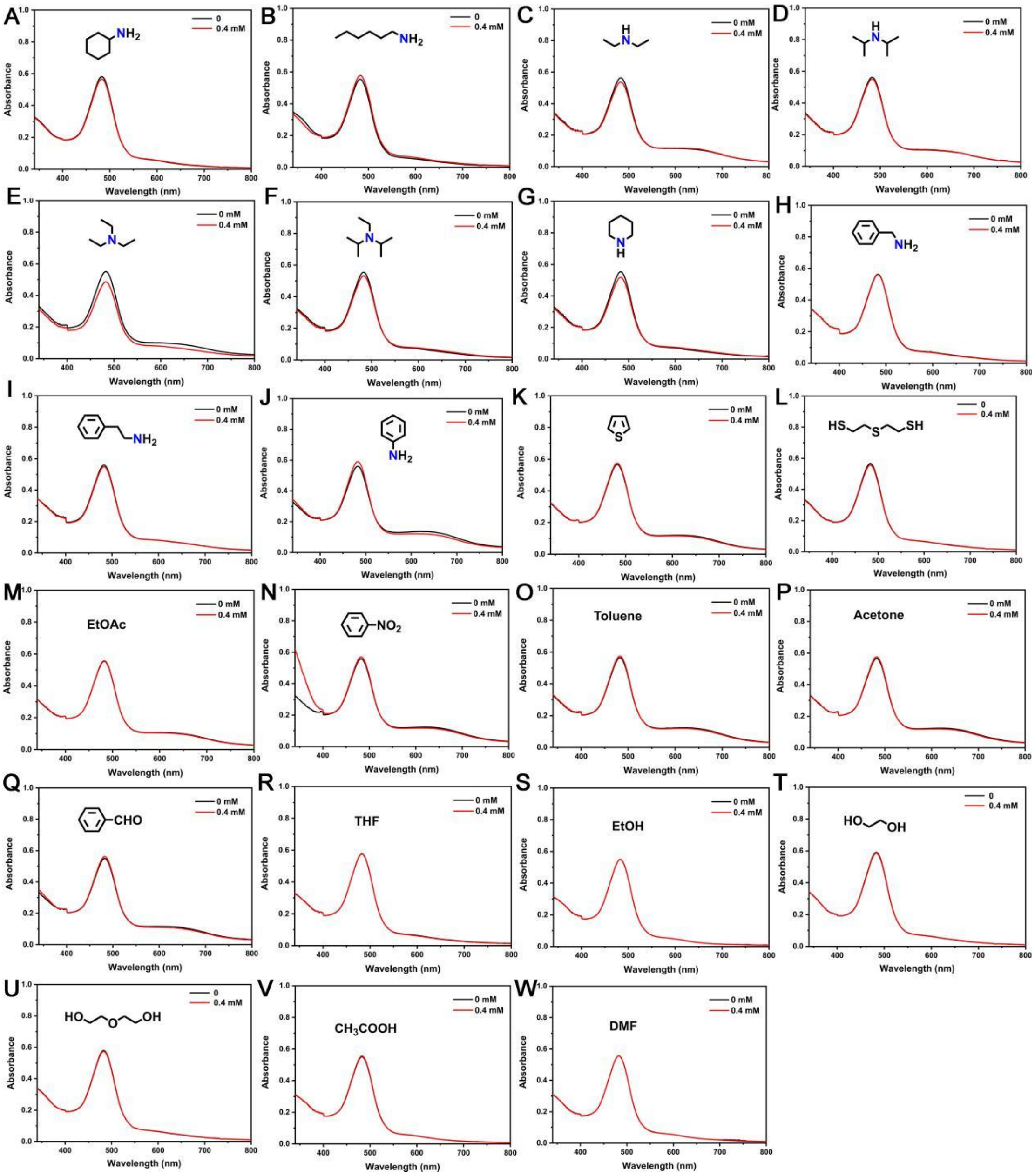

Figure S37. UV-vis spectra of **C5** (0.33 mM) before and after the addition of cyclohexylamine (A), hexylamine (B), diethylamine (C), diisopropylamine (D), triethylamine (E), *N,N*-diisopropylethylamine (F), piperidine (G), benzylamine (H), and phenethylamine (I), aniline (J), thiophene (K), 3-thiapentane-1,5-dithiolate (L), EtOAc (M), nitrobenzene (N), toluene (O), acetone (P), benzaldehyde (Q), THF (R), EtOH (S), ethylene glycol (T), diethylene glycol (U), CH_3COOH (V), and DMF (W) at the concentration of 0.40 mM.

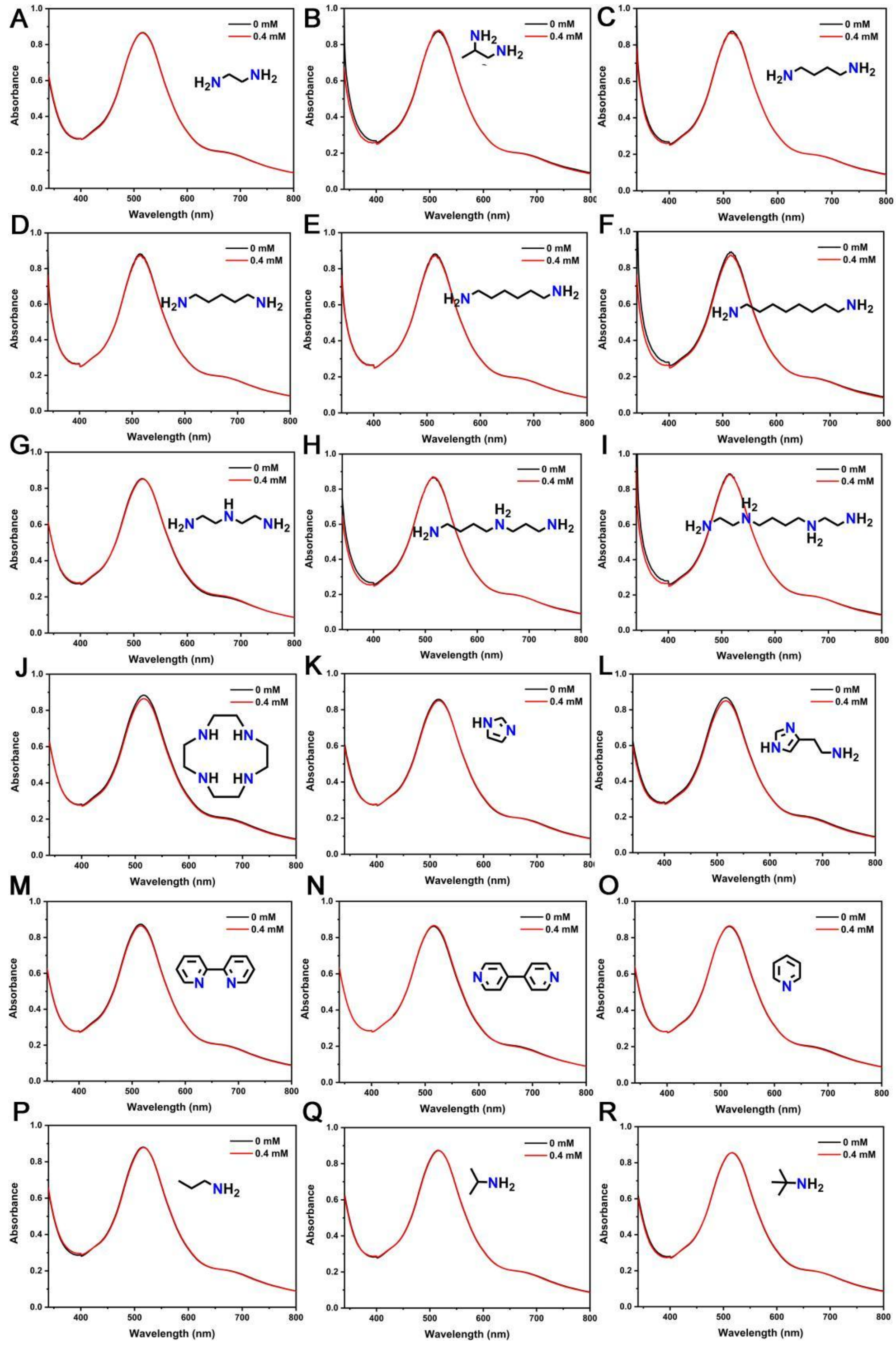

Figure S38. UV-vis spectra of **C6** (0.33 mM) before and after the addition of ethylenediamine (A), 1,2-diaminopropane (B), 1,4-diaminobutane (C), 1,5-diaminopentane (D), hexamethylenediamine (E), 1,8-diaminooctane (F), diethylenetriamine (G), spermidine (H), spermine (I), cyclen (J), imidazole (K), histamine (L), 2,2'-bipyrindine (M), 4,4'-bipyridine (N), pyridine (O), ^{n}propylpamine (P), isopropylamine (Q), and tert-butylamine (R).

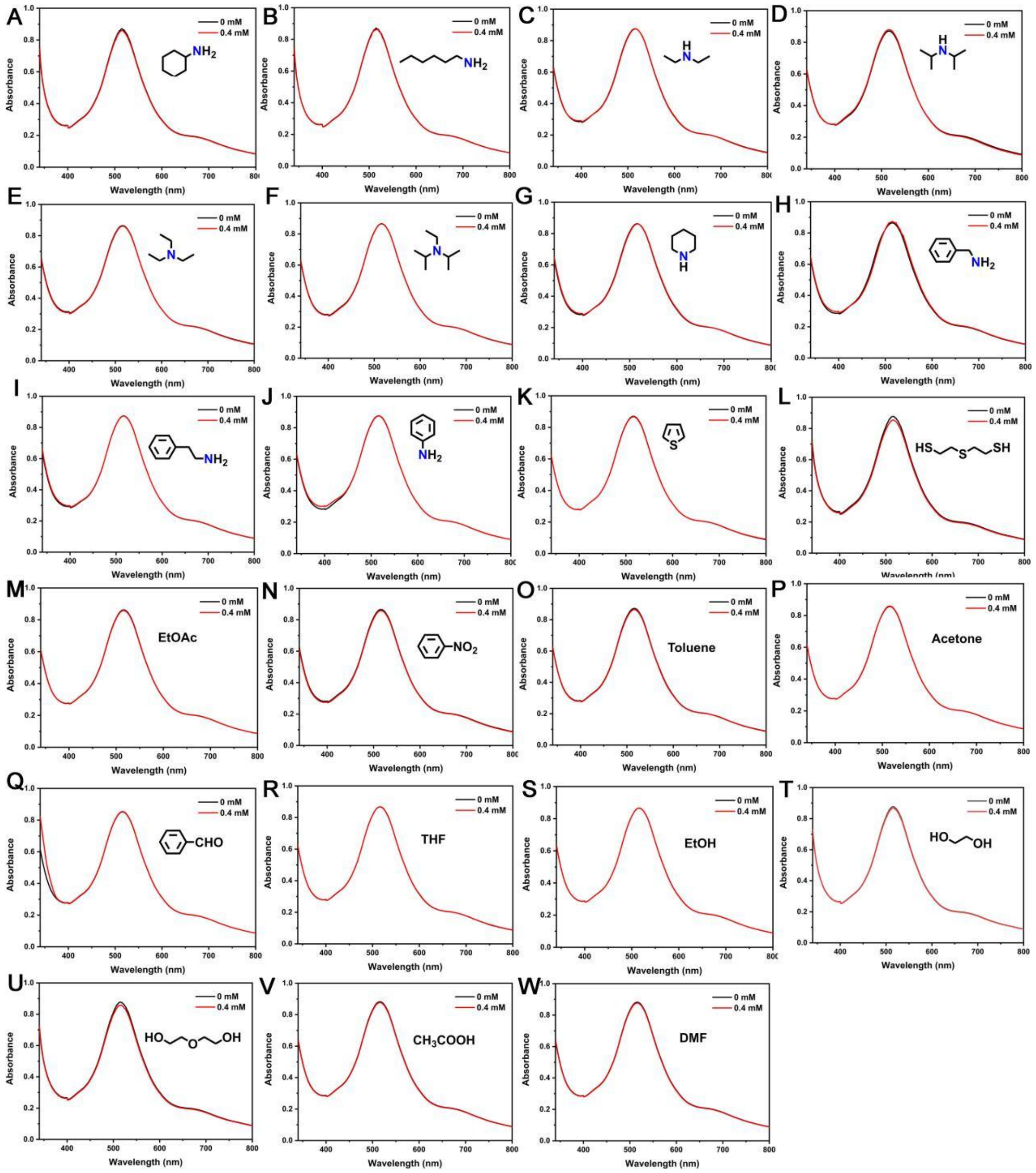

Figure S39. UV-vis spectra of **C6** (0.33 mM) before and after the addition of cyclohexylamine (A), hexylamine (B), diethylamine (C), diisopropylamine (D), triethylamine (E), *N,N*-diisopropylethylamine (F), piperidine (G), benzylamine (H), and phenethylamine (I), aniline (J), thiophene (K), 3-thiapentane-1,5-dithiolate (L), EtOAc (M), nitrobenzene (N), toluene (O), acetone (P), benzaldehyde (Q), THF (R), EtOH (S), ethylene glycol (T), diethylene glycol (U), CH_3COOH (V), and DMF (W) at the concentration of 0.40 mM.

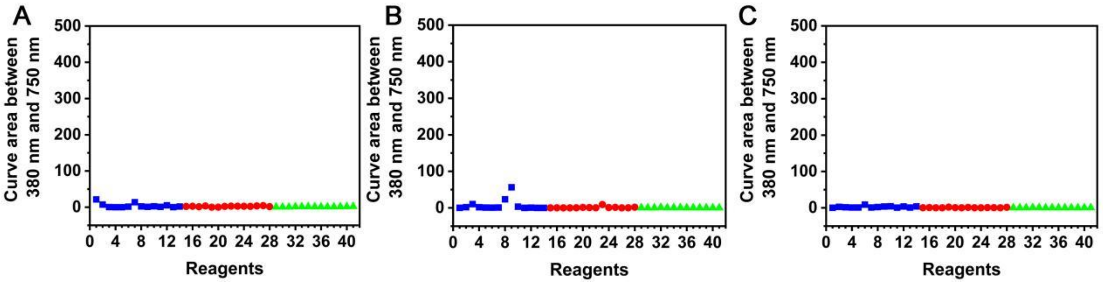

Figure S40. Summary of the negative responses of complexes **C4** (A), **C5** (B), and **C6** (C) to the above reagents.

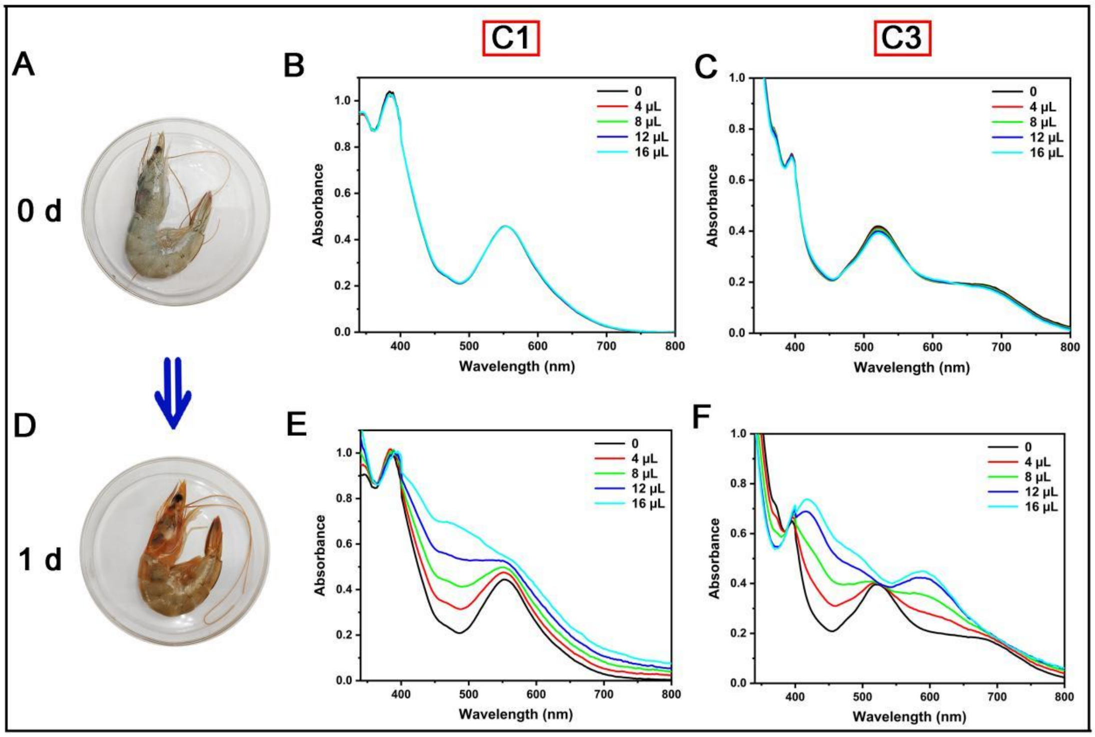

Figure S41. Monitoring fresh shrimp at 27 °C using our developed approach. (A, D) Photos of fresh shrimp (A) and shrimp left for one day (D). (B, C, E, F) UV-vis spectra of **C1** (B, E) and **C3** (C, F) after interaction with exudates of shrimp.

VII. HRMS Spectra

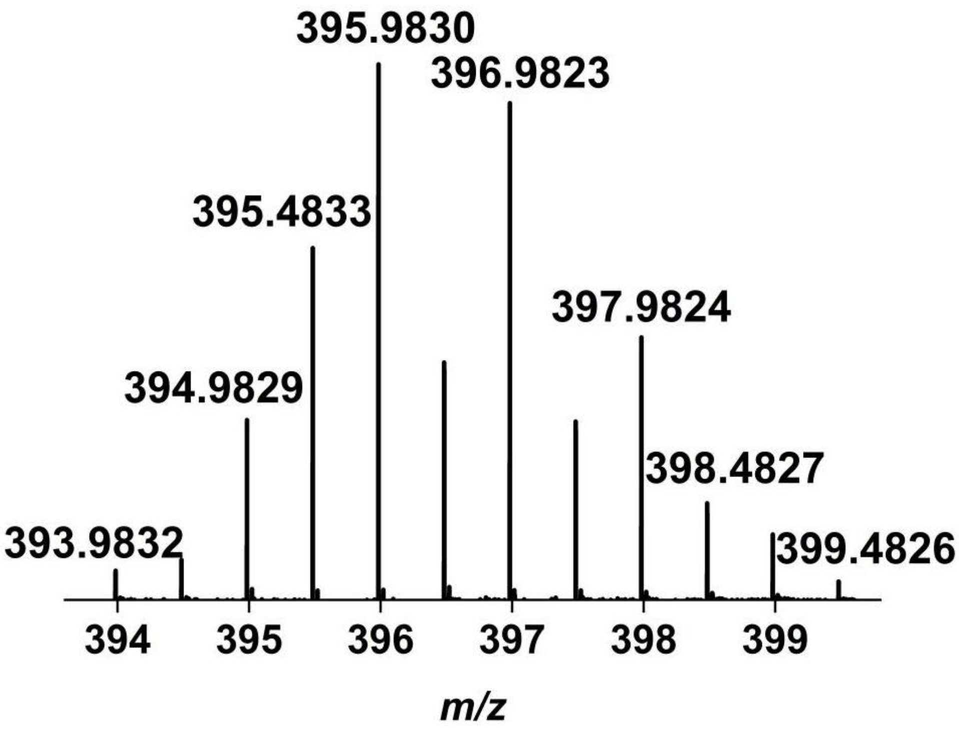

Figure S42. ESI-HRMS spectrum of **C4** in CH_3CN.

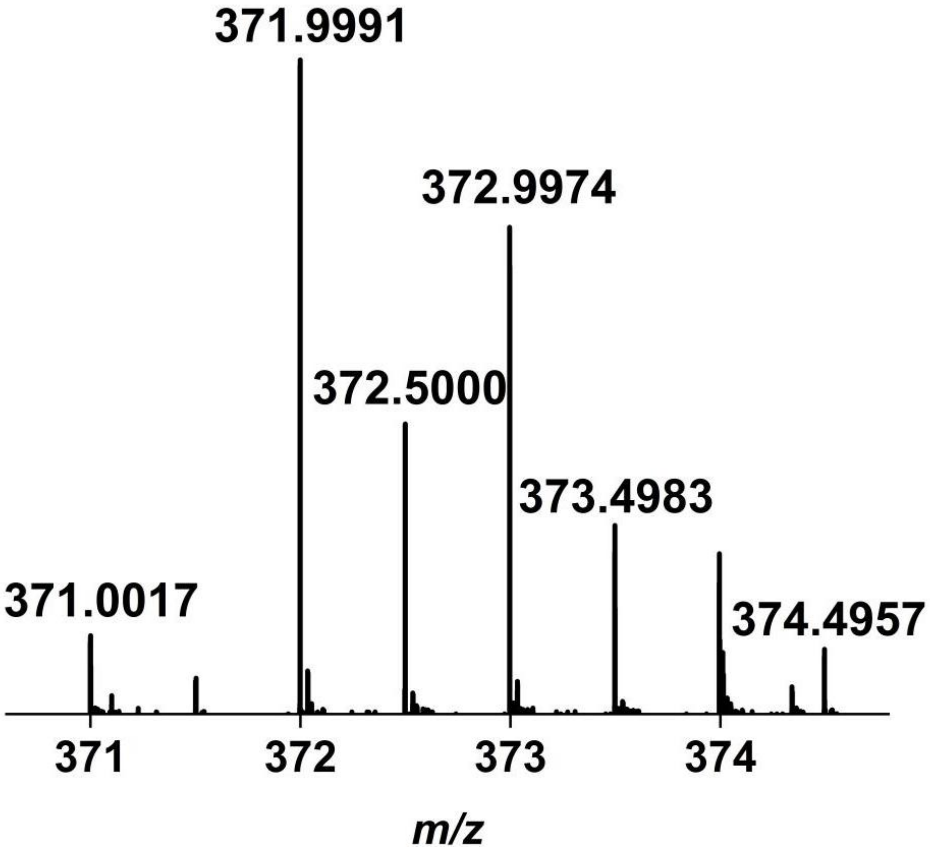

Figure S43. ESI-HRMS spectrum of **C5** in CH_2Cl_2.

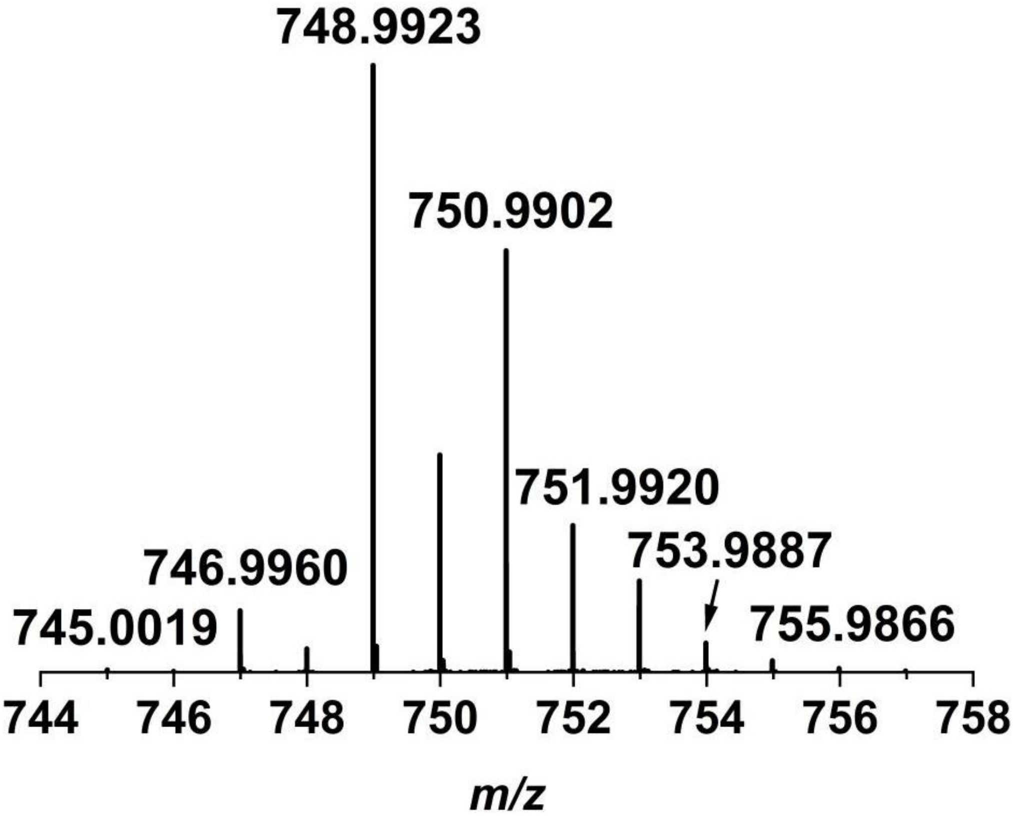

Figure S44. ESI-HRMS spectrum of **C6** in CH_2Cl_2.

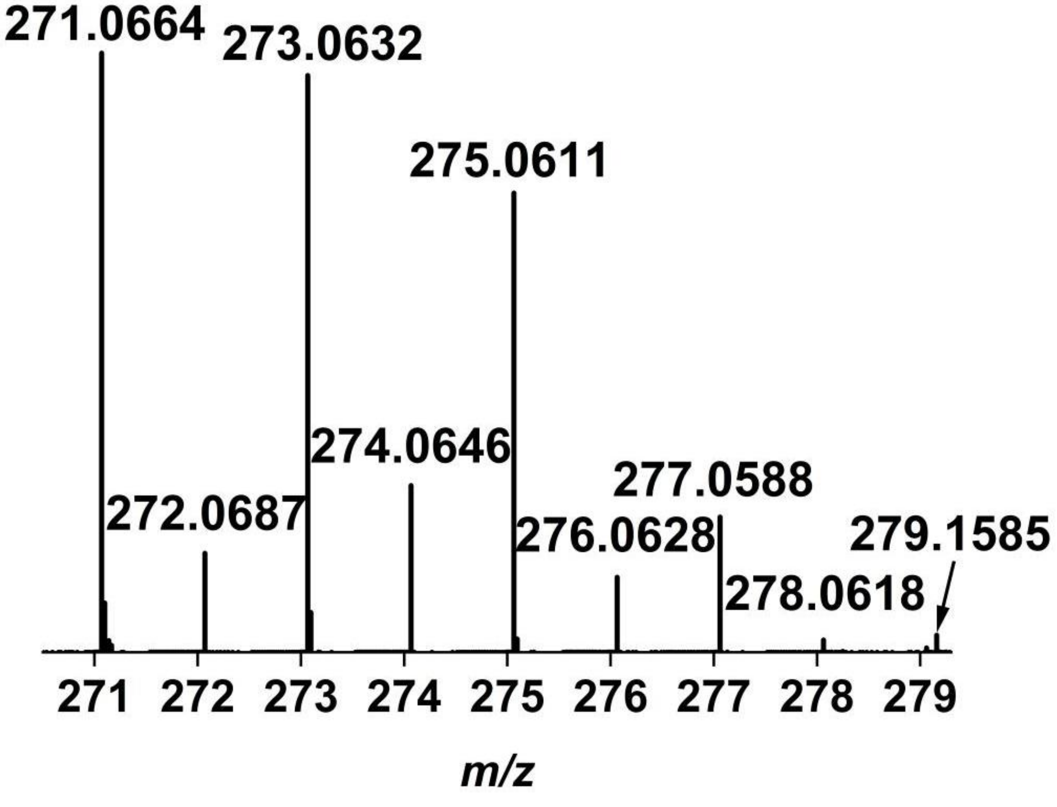

Figure S45. ESI-HRMS spectrum of **N1** in CH_3OH.

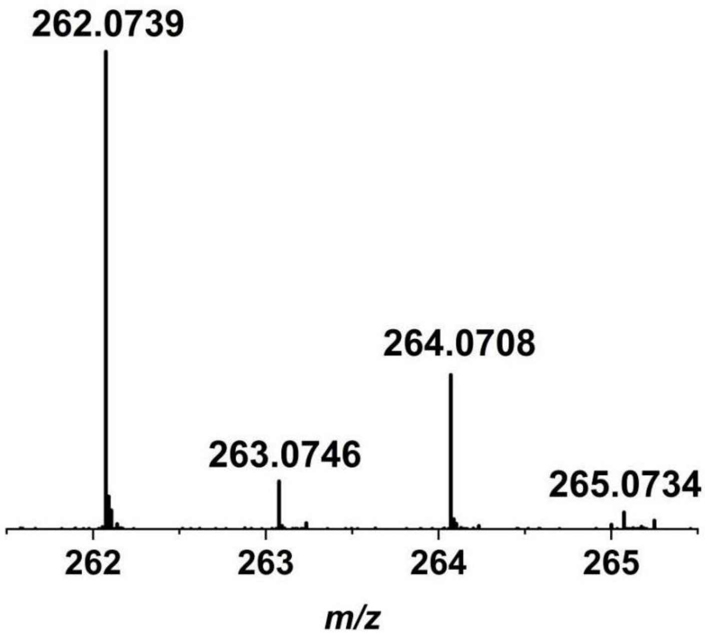

Figure S46. ESI-HRMS spectrum of **N3** in CH_3OH.

VIII. Electrochemical Spectra

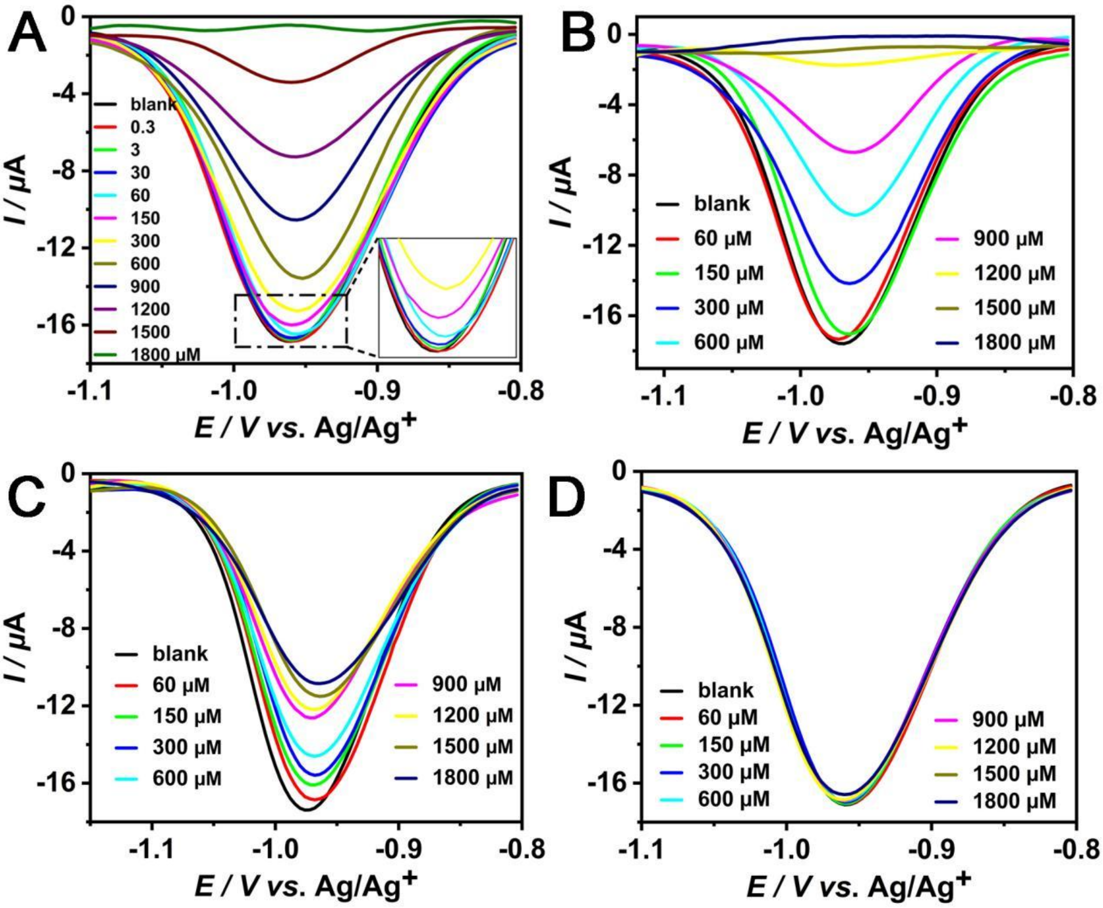

Figure S47. (A) DPV spectra of **C1** (1 mM) before and after the addition of cyclen at the concentration of 0.3, 3, 30, 60, 150, 300, 600, 900, 1200, 1500, and 1800 μM. (B–D) DPV spectra of **C1** (1 mM) before and after the addition of diethylenetriamine (B), 4,4'-bipyridine (C), and ^{n}propylamine (D) at the concentration of 60, 150, 300, 600, 900, 1200, 1500, and 1800 μM.

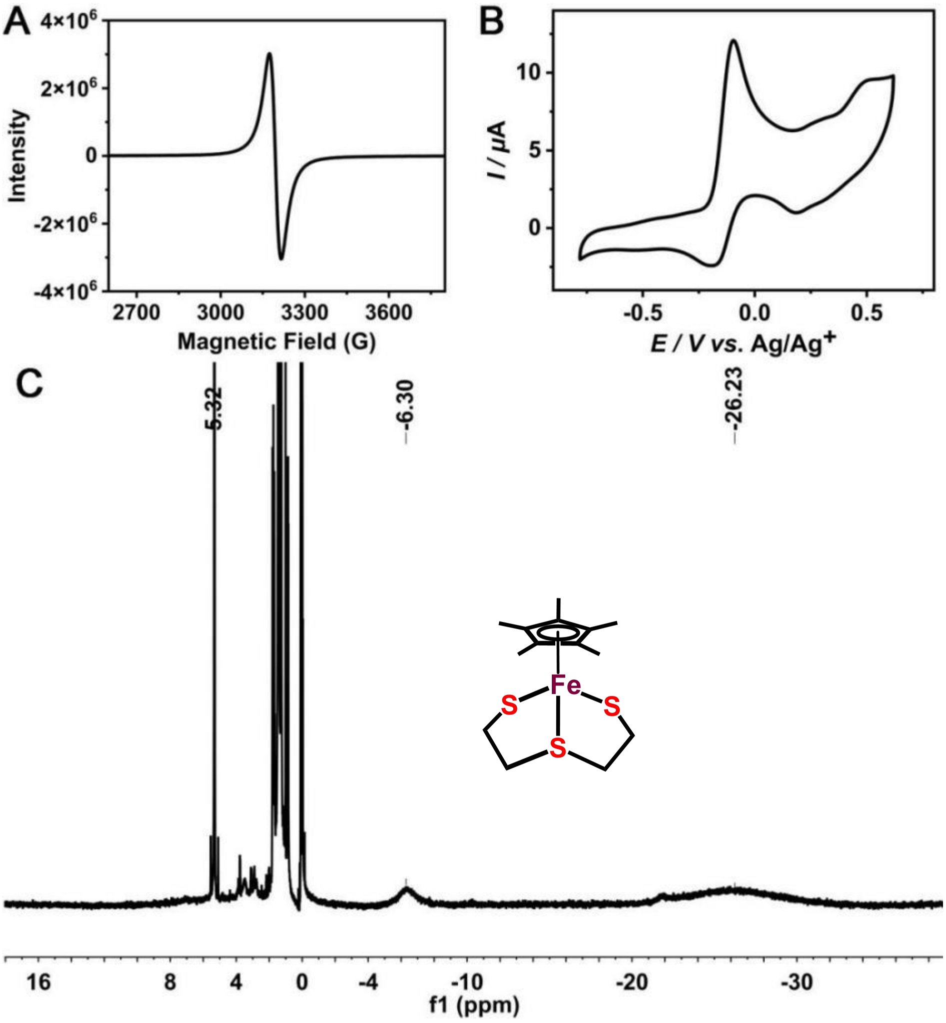

Figure S48. EPR (A), CV (B), and ^{1}H NMR (C) spectra of mononuclear [Cp*Fe(η^3-tpdt)] precursor.

Note: EPR and CV spectra were recorded in CH_2Cl_2 at the concentration of 1 mM. ^{1}H NMR spectrum was performed in CD_2Cl_2.

IX. IR Spectra

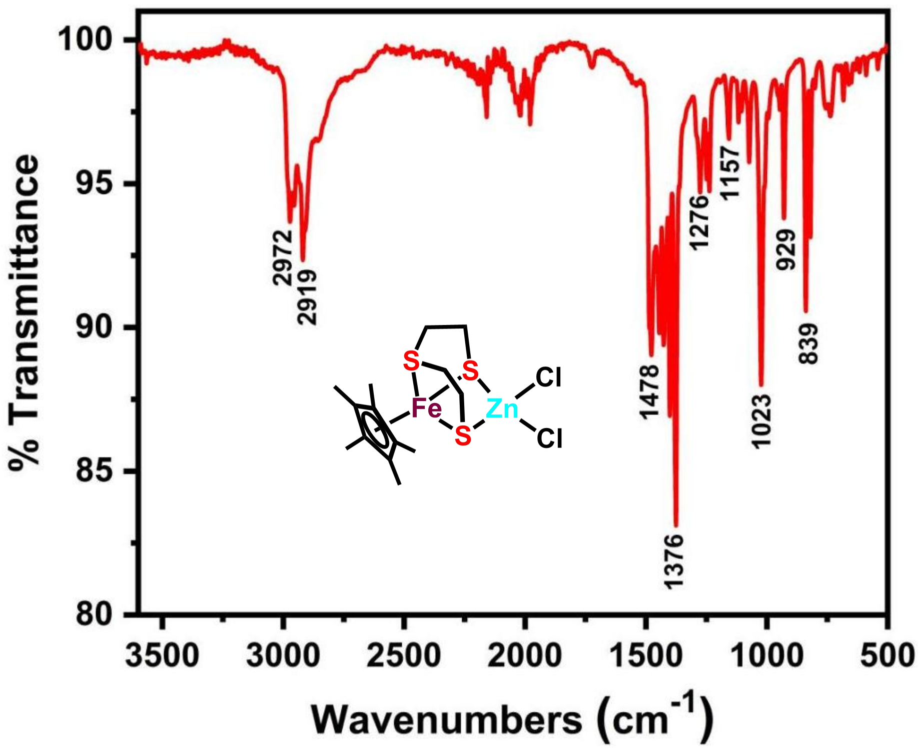

Figure S49. FT-IR spectrum of **C1**.

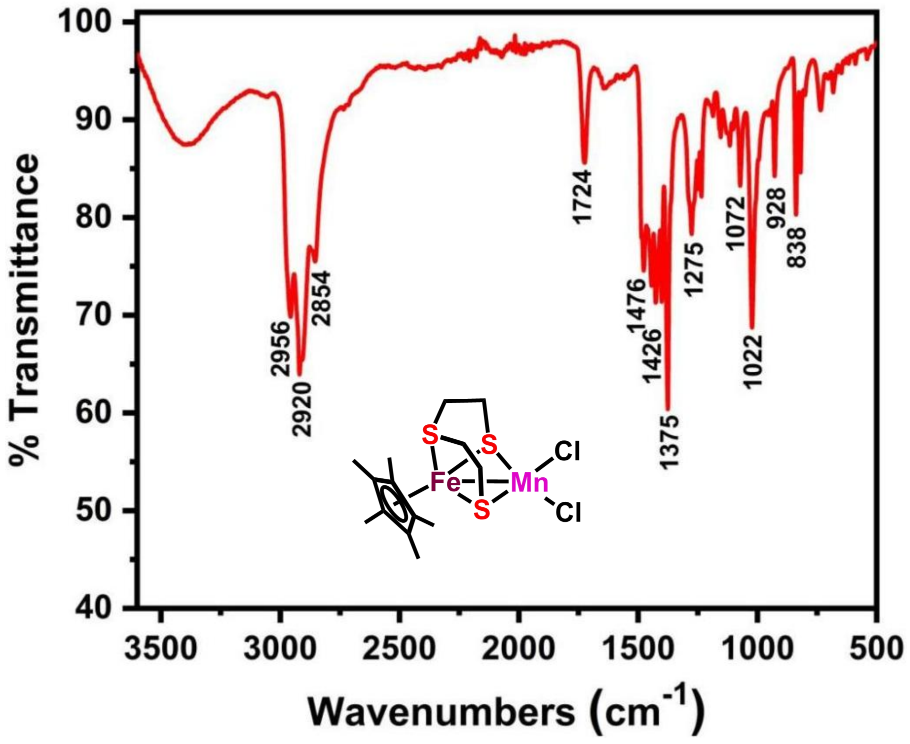

Figure S50. FT-IR spectrum of **C3**.

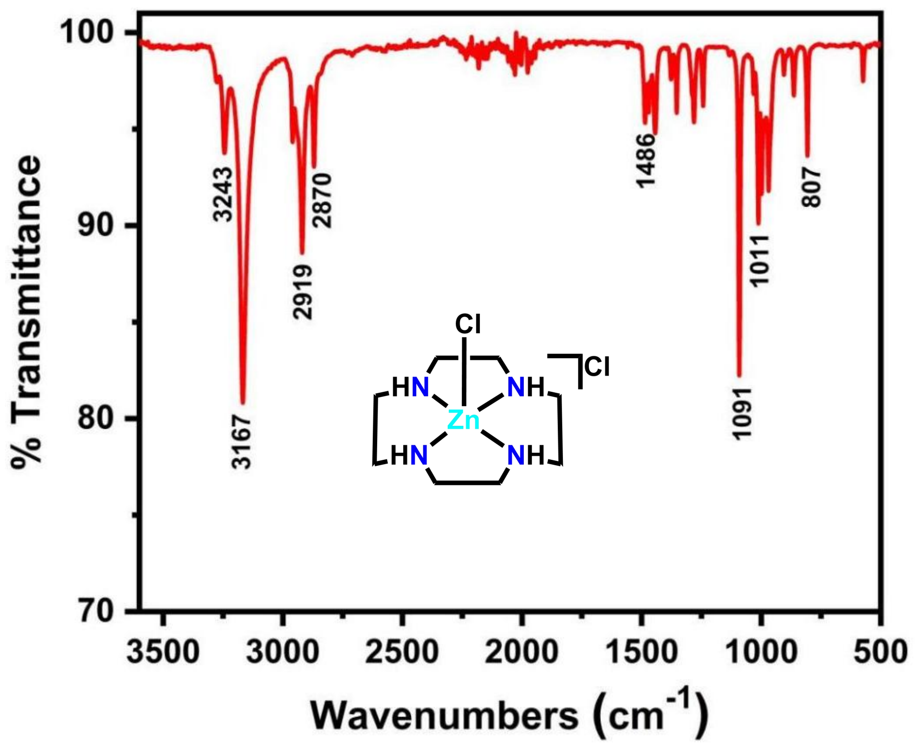

Figure S51. FT-IR spectrum of **N1**.

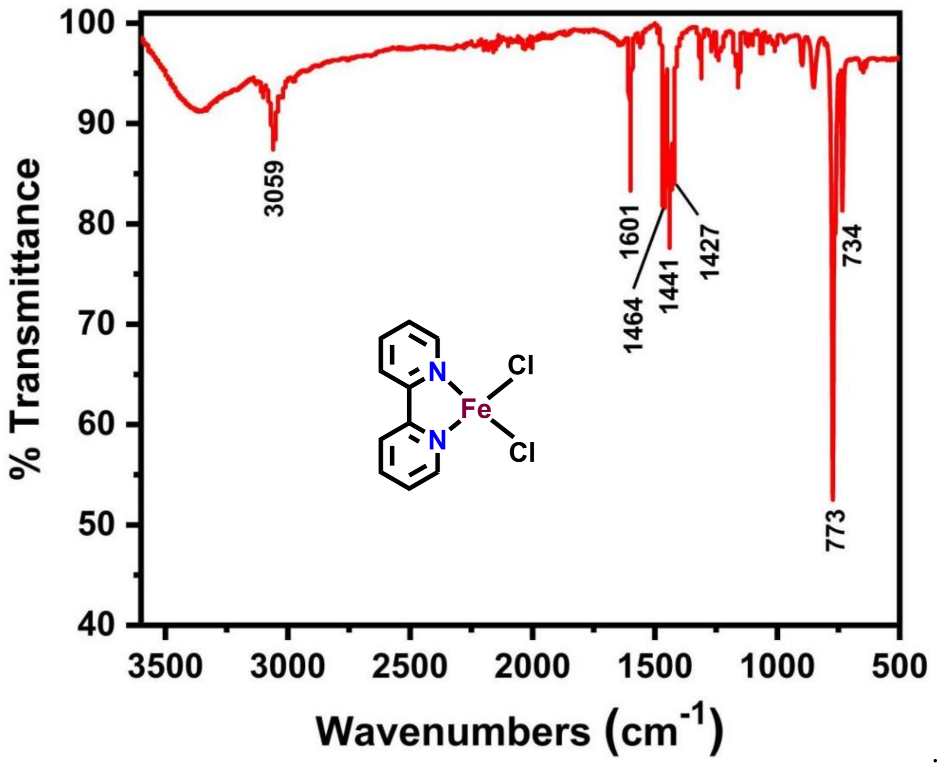

Figure S52. FT-IR spectrum of **N2**.

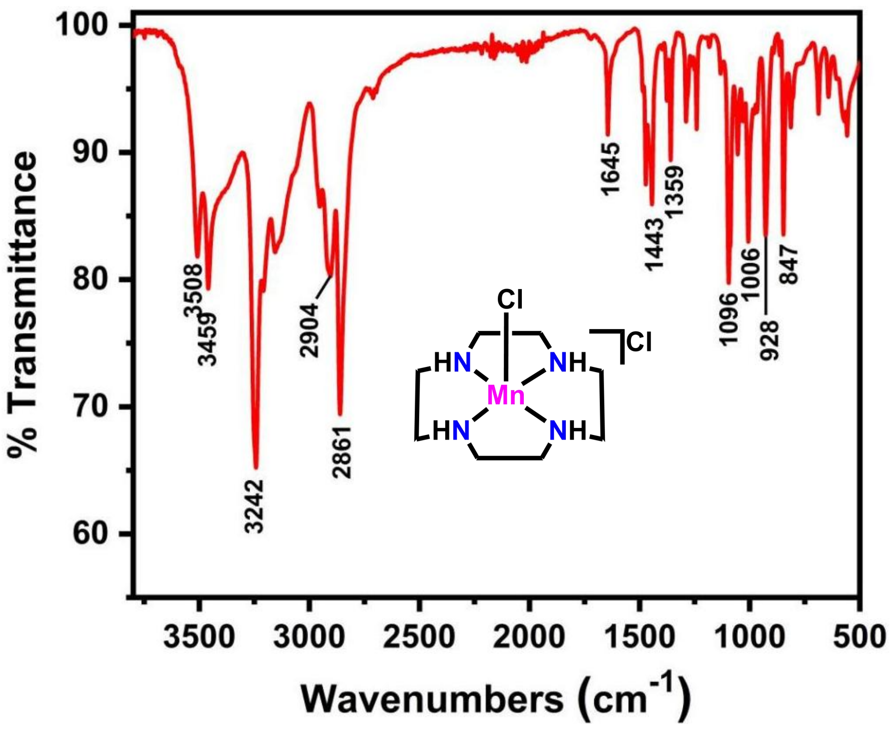

Figure S53. FT-IR spectrum of **N3**.

X. Morphological Data

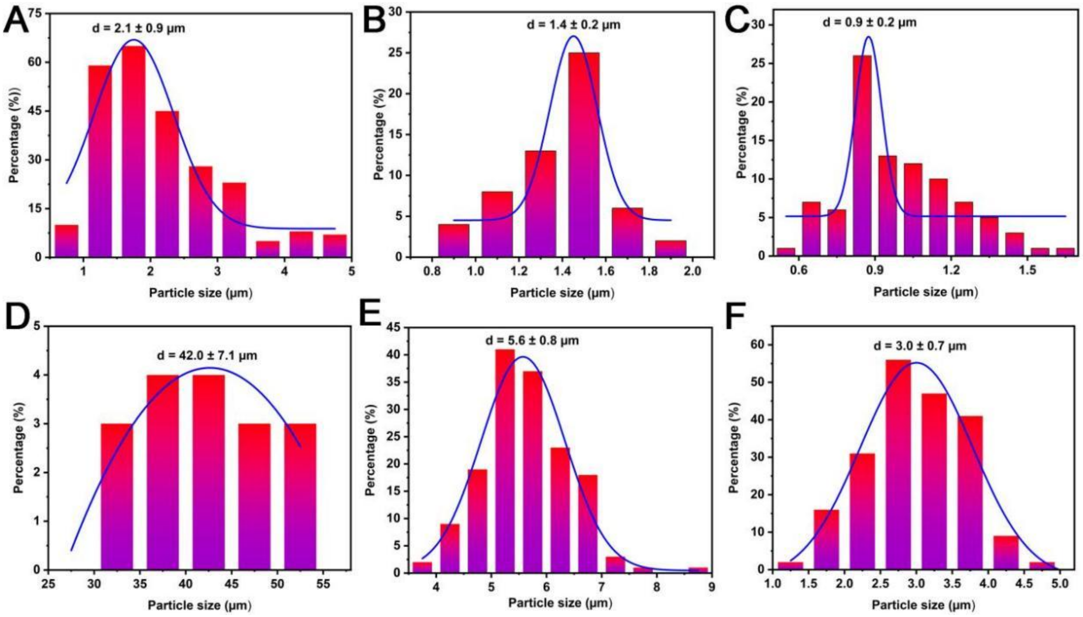

Figure S54. Diameter distribution of **C1** (A), Zn^{2+}–cyclen (**N1**, B, C), Zn^{2+}–ethylenediamine (D), Zn^{2+}–4,4'-bipyridine (E) and Fe^{2+}–4,4'-bipyridine (F).

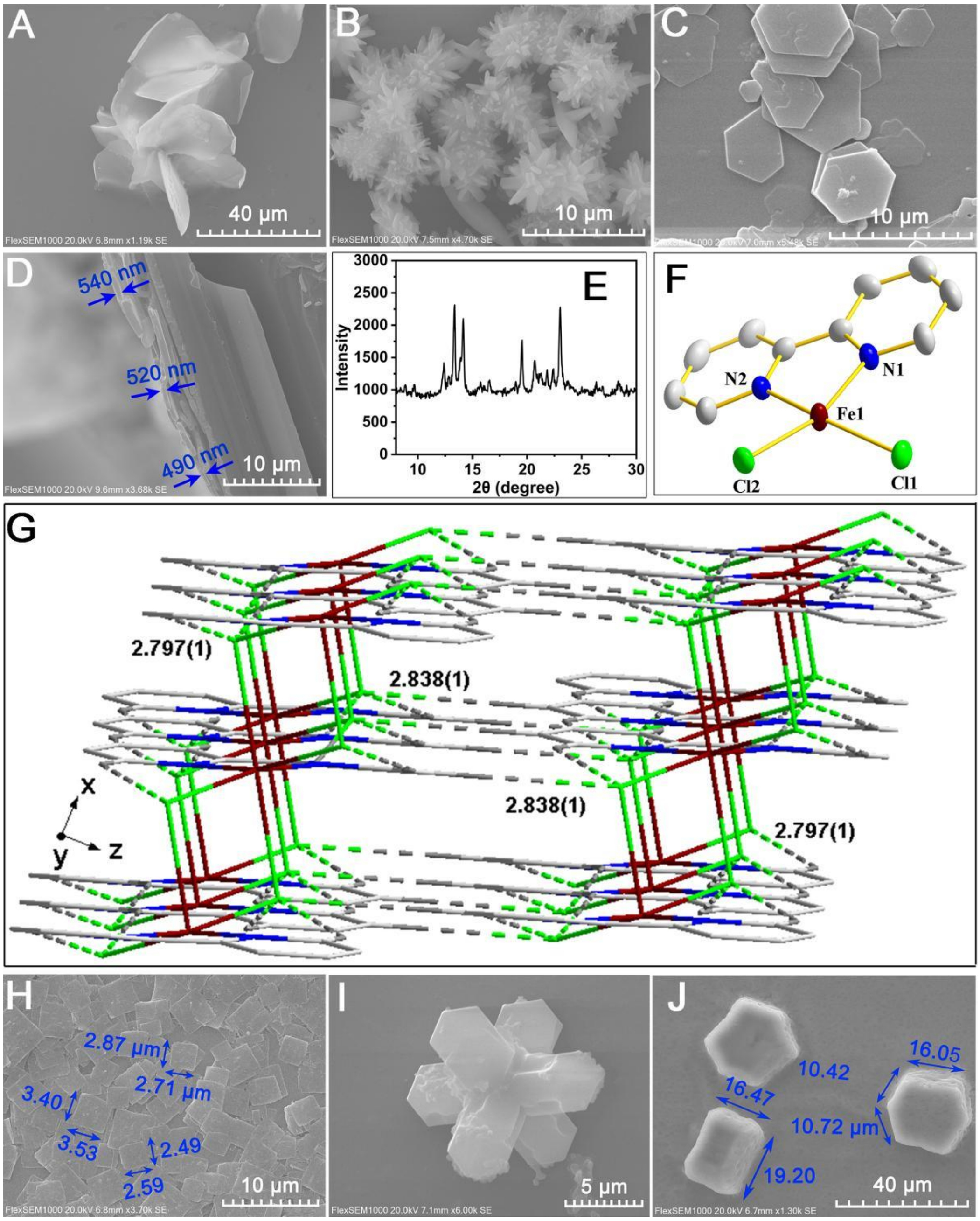

Figure S55. Characterization data of **C1** and **C2** after interaction with various organic amines. (A, B) SEM images of **C1** (5.0 mM) after the addition of ethylenediamine (A, 6.0 mM) or 4,4'-bipyridine (B, 150 mM). (C, D) SEM image (C) and cross-sectional SEM image (D) of **C2** (5.0 mM) in the presence of 2,2'-bipyridine (150 mM). (E–G) PXRD pattern (E), crystal structure (F), and packing mode (G) of complex [(η^2-2,2'-bipyridine)$FeCl_2$] (**N2**). (H–J) SEM images of **C2** (5.0 mM) after interaction with 4,4'-bipyridine (H, 150 mM), iminazole (I, 6.0 mM), or histamine (J, 150 mM), respectively.

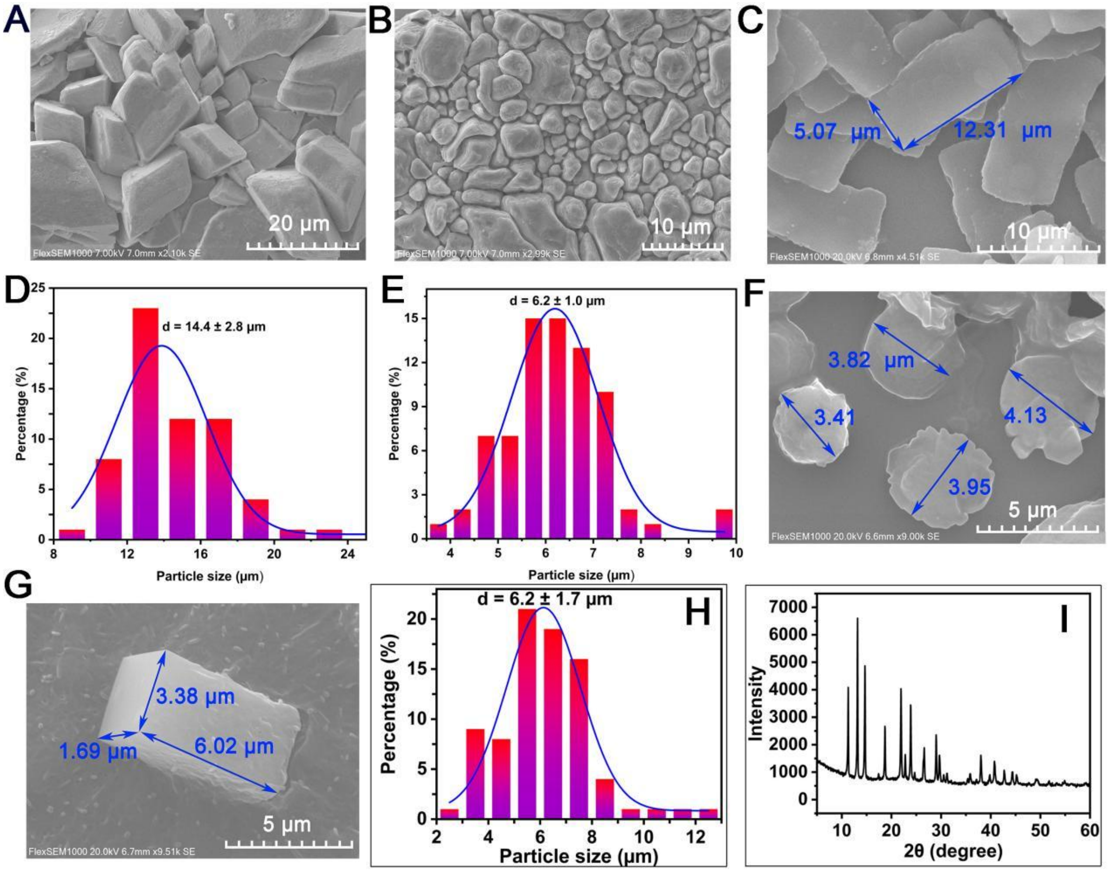

Figure S56. (A–C) SEM images of **C2** (A), **C3** (B), and Mn^{2+}–diethylenetriamine (**N3**, C). (D, E) Diameter distribution of **N3** in length (D) and width (E). (F, G) SEM images of Mn^{2+}–iminazole (F) and Mn^{2+}–cyclen (G). (H, I) Diameter distribution (H) and XRD pattern (I) of Mn^{2+}–cyclen.

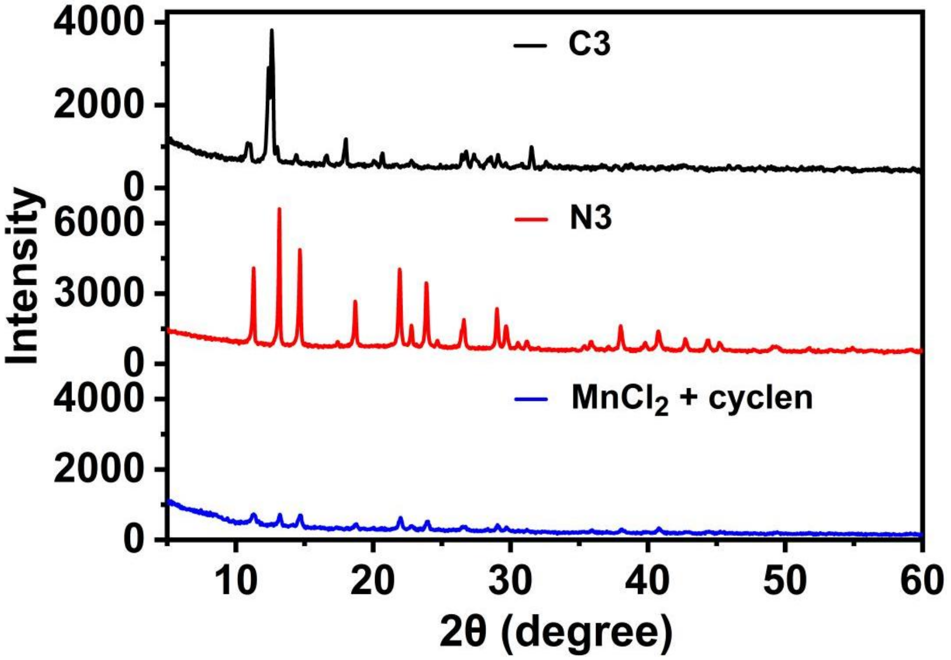

Figure S57. XRD pattern of **C3**, **N3**, and the reaction product of $MnCl_2$ and cyclen obtained in CH_3OH.

Note: The reaction product of $MnCl_2$ with cyclen was obtained in CH_3OH. They were reacted at a 1:1 molar ratio for 15 minutes at room temperature, and then the mixture was dried under a vacuum to obtain the product powder.

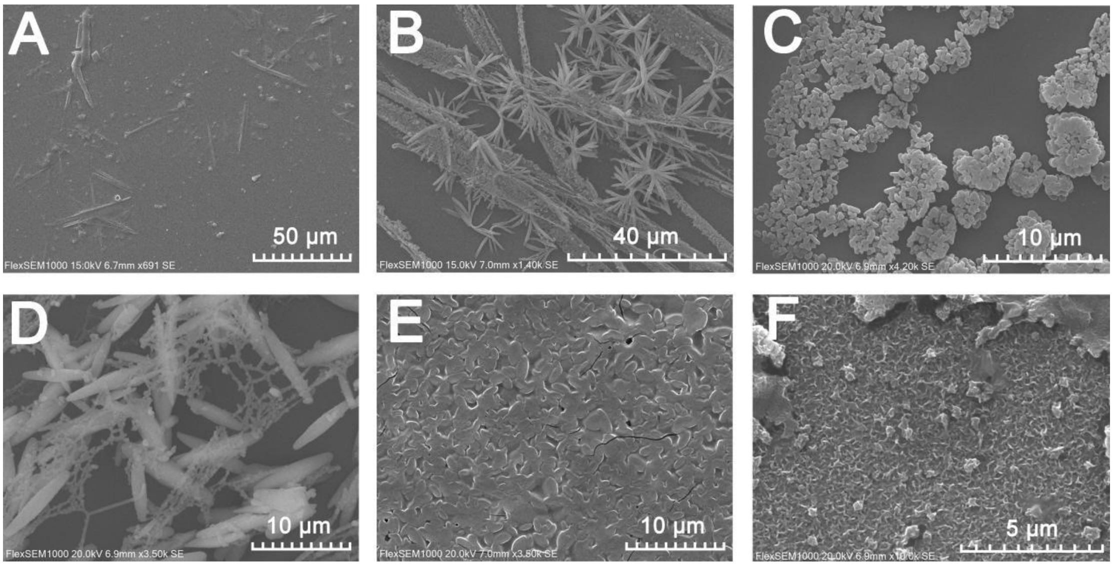

Figure S58. SEM images of the reaction products between $ZnCl_2$ and cyclen (A) or 4,4'-bipyridine (B), $FeCl_2$ and 2,2'-bipyridine (C) or 4,4'-bipyridine (D), $MnCl_2$ and cyclen (E) or diethylenetriamine (F).

Note: All reaction products were obtained in CH_3OH with a 1:1 molar ratio of metal

salts and organic amines. After stirring for 15 minutes at room temperature, several drops of these mixtures were taken on silicon wafers for SEM testing. As shown in Figures A–F, assembly morphologies of these reaction products were quite different from those driven by the self-sacrifice reaction of iron-sulfur complexes. For example, little regular assembly was shown after the reaction of $ZnCl_2$ and cyclen (A), as well as the products after the reaction of $FeCl_2$ and 2,2'-bipyridine (C), $MnCl_2$ and cyclen (E) or diethylenetriamine (F). Silk petals (B) were generated in the presence of $ZnCl_2$ and 4,4'-bipyridine (long strip compounds were attributed to unreacted 4,4'-bipyridine). Two types of assemblies (oval and beaded morphology) were shown after the reaction of $FeCl_2$ and 4,4'-bipyridine (D).

XI. Cyclic Detection

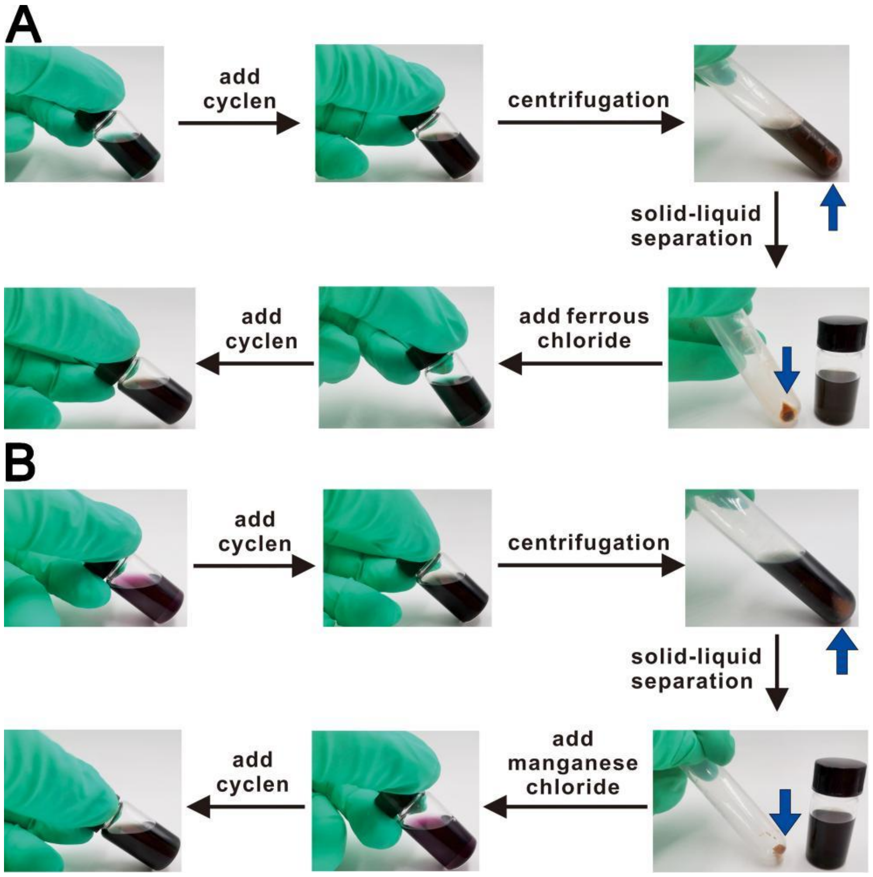

Figure S59. Reaction mechanism of **C2** (A) and **C3** (B) for cyclic detection of cyclen.

Supplementary References

(2) Li, Y.; Zhang, Y.; Yang, D.; Li, Y.; Sun, P.; Wang, B.; Qu, J. Synthesis and Reactivity of Thioether-Dithiolate-Bridged Multi-iron Complexes. *Organometallics* **2015**, *34*, 1661–1667.

(3) Sheldrick, G. M. SADABS. *Program for Area Detector Adsorption Correction; Institute for Inorganic Chemistry*, University of Göttingen: Göttingen, Germany, **1996**.

(a) Sheldrick, G. M. *SHELXL-2014, Program for Refinement of Crystal Structures*; University of Göttingen: Göttingen, Germany, **2014**. (b) Sheldrick, G. M. SHELXS-2014, *Program for Solution of Crystal Structures*; University of Gottingen: Göttingen, Germany, **2014**.
