# Supplementary material for: Order–order assembly transition-driven polyamines detection based on iron−sulfur complexes
Source: Commun Chem. 2023 Jul 7;6:146. doi: 10.1038/s42004-023-00942-1 (PMC10328931; doi:10.1038/s42004-023-00942-1)
Supplement: Supplementary file 3 — Description of Additional Supplementary Files [file 42004_2023_942_MOESM3_ESM.pdf]

Description of Additional Supplementary Files

File name: Supplementary Movie S1

Description: Recyclability of C1 in cyclen detection assays

File name: Supplementary Data 1

Description: the cif file of C1

File name: Supplementary Data 2

Description: the cif file of C3

File name: Supplementary Data 3

Description: the cif file of N1

File name: Supplementary Data 4

Description: the cif file of N2

File name: Supplementary Data 5

Description: the cif file of N3

File name: Supplementary Data 6

Description: the checkcif file of C1

File name: Supplementary Data 7

Description: the checkcif file of C3

File name: Supplementary Data 8

Description: the checkcif file of N1

File name: Supplementary Data 9

Description: the checkcif file of N2

File name: Supplementary Data 10

Description: the checkcif file of N3
