# Supplementary material for: Order–order assembly transition-driven polyamines detection based on iron−sulfur complexes
Source: Commun Chem. 2023 Jul 7;6:146. doi: 10.1038/s42004-023-00942-1 (PMC10328931; doi:10.1038/s42004-023-00942-1)

checkCIF/PLATON report

You have not supplied any structure factors. As a result the full set of tests cannot be run.

THIS REPORT IS FOR GUIDANCE ONLY. IF USED AS PART OF A REVIEW PROCEDURE FOR PUBLICATION, IT SHOULD NOT REPLACE THE EXPERTISE OF AN EXPERIENCED CRYSTALLOGRAPHIC REFEREE.

No syntax errors found. CIF dictionary Interpreting this report

Datablock: 2

```
Bond precision:   C-C = 0.0216 A          Wavelength=0.71073

Cell:               a=8.683(3)     b=14.267(6)         c=16.095(6)
                    alpha=90       beta=79.403(7)      gamma=90
Temperature:        296 K

                 Calculated                    Reported
Volume           1959.9(13)                    1959.8(13)
Space group      P 21                          P 21
Hall group       P 2yb                         P 2yb
Moiety formula   C14 H23 Cl2 Fe S3 Zn          ?
Sum formula      C14 H23 Cl2 Fe S3 Zn          C14 H23 Cl2 Fe S3 Zn
Mr               479.64                        479.62
Dx,g cm-3        1.625                         1.626
Z                4                             4
Mu (mm-1)        2.547                         2.547
F000             980.0                         980.0
F000'            985.32
h,k,lmax         10,16,19                      10,16,19
Nref             6914[ 3611]                   6793
Tmin,Tmax        0.619,0.756                   0.825,0.896
Tmin'            0.580

Correction method= # Reported T Limits: Tmin=0.825 Tmax=0.896
AbsCorr = EMPIRICAL

Data completeness= 1.88/0.98       Theta(max)= 24.993

                                                         wR2(reflections)=
R(reflections)= 0.0576( 6066)                            0.1681( 6793)

S = 1.092                   Npar= 379
```

The following ALERTS were generated. Each ALERT has the format
test-name_ALERT_alert-type_alert-level.
Click on the hyperlinks for more details of the test.

Alert level B

```
PLAT341_ALERT_3_B Low Bond Precision on  C-C Bonds ..............    0.02158 Ang.
```

Alert level C

```
PLAT157_ALERT_4_C Non-standard Monoclinic Beta Angle less 90 Deg       79.40 Degree
PLAT234_ALERT_4_C Large Hirshfeld Difference S3       --C23      .      0.17 Ang.
PLAT234_ALERT_4_C Large Hirshfeld Difference S6       --C25      .      0.19 Ang.
PLAT234_ALERT_4_C Large Hirshfeld Difference C25      --C26      .      0.18 Ang.
PLAT234_ALERT_4_C Large Hirshfeld Difference C27      --C28      .      0.18 Ang.
PLAT241_ALERT_2_C High   'MainMol' Ueq as Compared to Neighbors of       C21 Check
PLAT241_ALERT_2_C High   'MainMol' Ueq as Compared to Neighbors of       C23 Check
PLAT241_ALERT_2_C High   'MainMol' Ueq as Compared to Neighbors of       C25 Check
PLAT241_ALERT_2_C High   'MainMol' Ueq as Compared to Neighbors of       C28 Check
PLAT242_ALERT_2_C Low    'MainMol' Ueq as Compared to Neighbors of        S3 Check
PLAT242_ALERT_2_C Low    'MainMol' Ueq as Compared to Neighbors of       Fe2 Check
PLAT360_ALERT_2_C Short  C(sp3)-C(sp3) Bond  C23      - C24      .      1.39 Ang.
PLAT360_ALERT_2_C Short  C(sp3)-C(sp3) Bond  C27      - C28      .      1.38 Ang.
```

Alert level G

```
PLAT003_ALERT_2_G Number of Uiso or Uij Restrained non-H Atoms ...        42 Report
PLAT158_ALERT_4_G The Input Unitcell is NOT Standard/Reduced .....    Please Check
PLAT186_ALERT_4_G The CIF-Embedded .res File Contains ISOR Records         1 Report
PLAT380_ALERT_4_G Incorrectly? Oriented X(sp2)-Methyl Moiety .....        C6 Check
PLAT380_ALERT_4_G Incorrectly? Oriented X(sp2)-Methyl Moiety .....        C7 Check
PLAT380_ALERT_4_G Incorrectly? Oriented X(sp2)-Methyl Moiety .....        C8 Check
PLAT380_ALERT_4_G Incorrectly? Oriented X(sp2)-Methyl Moiety .....        C9 Check
PLAT380_ALERT_4_G Incorrectly? Oriented X(sp2)-Methyl Moiety .....       C10 Check
PLAT380_ALERT_4_G Incorrectly? Oriented X(sp2)-Methyl Moiety .....       C16 Check
PLAT380_ALERT_4_G Incorrectly? Oriented X(sp2)-Methyl Moiety .....       C17 Check
PLAT380_ALERT_4_G Incorrectly? Oriented X(sp2)-Methyl Moiety .....       C18 Check
PLAT380_ALERT_4_G Incorrectly? Oriented X(sp2)-Methyl Moiety .....       C19 Check
PLAT380_ALERT_4_G Incorrectly? Oriented X(sp2)-Methyl Moiety .....       C20 Check
PLAT794_ALERT_5_G Tentative Bond Valency for Zn1       (II)       .      1.95 Info
PLAT794_ALERT_5_G Tentative Bond Valency for Zn2       (II)       .      1.98 Info
PLAT794_ALERT_5_G Tentative Bond Valency for Fe1       (III)      .      3.73 Info
PLAT794_ALERT_5_G Tentative Bond Valency for Fe2       (III)      .      3.69 Info
PLAT860_ALERT_3_G Number of Least-Squares Restraints .............       253 Note
PLAT883_ALERT_1_G No Info/Value for _atom_sites_solution_primary .    Please Do !
PLAT941_ALERT_3_G Average HKL Measurement Multiplicity ...........       2.8 Low
PLAT965_ALERT_2_G The SHELXL WEIGHT Optimisation has not Converged    Please Check
PLAT967_ALERT_5_G Note: Two-Theta Cutoff Value in Embedded .res ..      50.0 Degree
```

0 **ALERT level A** = Most likely a serious problem - resolve or explain
1 **ALERT level B** = A potentially serious problem, consider carefully
13 **ALERT level C** = Check. Ensure it is not caused by an omission or oversight
22 **ALERT level G** = General information/check it is not something unexpected

```
 1 ALERT type 1 CIF construction/syntax error, inconsistent or missing data
10 ALERT type 2 Indicator that the structure model may be wrong or deficient
 3 ALERT type 3 Indicator that the structure quality may be low
17 ALERT type 4 Improvement, methodology, query or suggestion
 5 ALERT type 5 Informative message, check
```

It is advisable to attempt to resolve as many as possible of the alerts in all categories. Often the minor alerts point to easily fixed oversights, errors and omissions in your CIF or refinement strategy, so attention to these fine details can be worthwhile. In order to resolve some of the more serious problems it may be necessary to carry out additional measurements or structure refinements. However, the purpose of your study may justify the reported deviations and the more serious of these should normally be commented upon in the discussion or experimental section of a paper or in the "special_details" fields of the CIF. checkCIF was carefully designed to identify outliers and unusual parameters, but every test has its limitations and alerts that are not important in a particular case may appear. Conversely, the absence of alerts does not guarantee there are no aspects of the results needing attention. It is up to the individual to critically assess their own results and, if necessary, seek expert advice.

Publication of your CIF in IUCr journals

A basic structural check has been run on your CIF. These basic checks will be run on all CIFs submitted for publication in IUCr journals (*Acta Crystallographica*, *Journal of Applied Crystallography*, *Journal of Synchrotron Radiation*); however, if you intend to submit to *Acta Crystallographica Section C* or *E* or *IUCrData*, you should make sure that full publication checks are run on the final version of your CIF prior to submission.

Publication of your CIF in other journals

Please refer to the *Notes for Authors* of the relevant journal for any special instructions relating to CIF submission.

PLATON version of 19/02/2022; check.def file version of 19/02/2022

Datablock 2 - ellipsoid plot

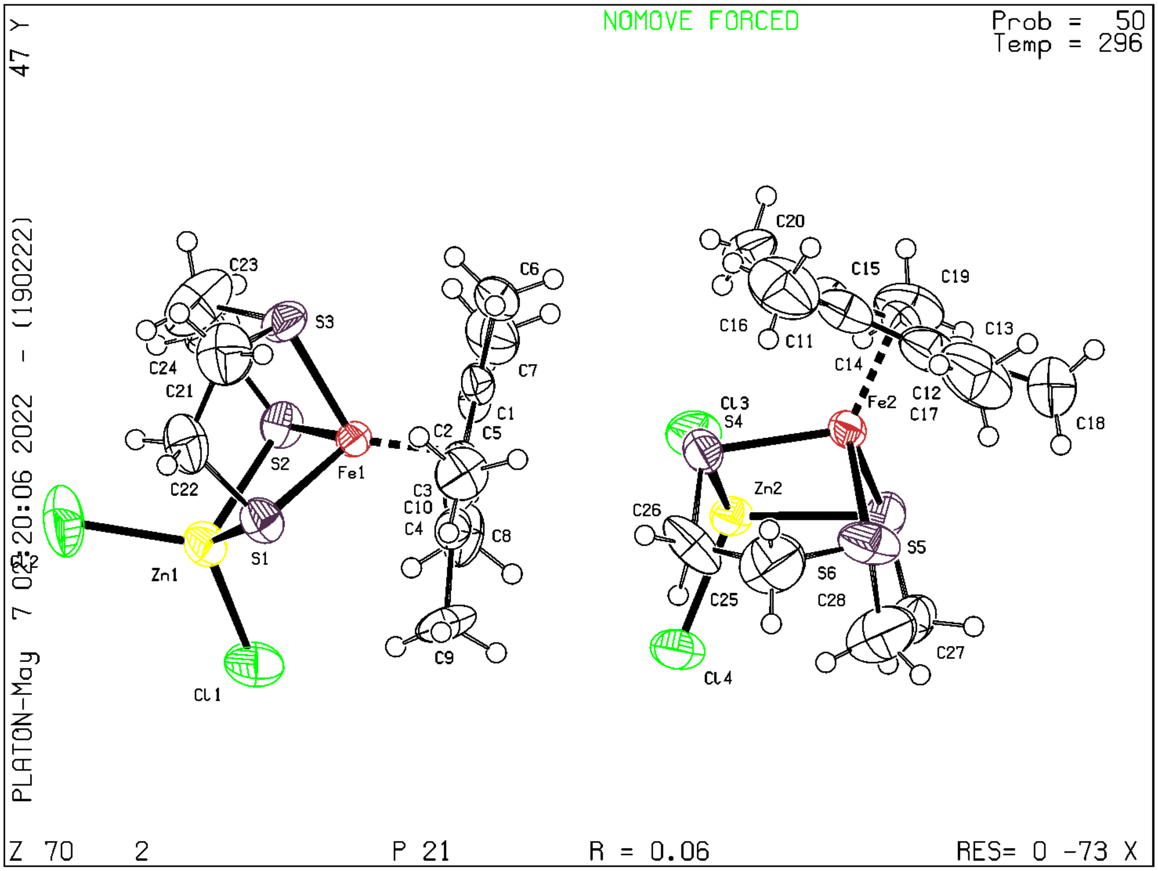

Supplement: Supplementary file 9 — Supplementary Data 6 [file 42004_2023_942_MOESM9_ESM.pdf]
