# Supplementary material for: Order–order assembly transition-driven polyamines detection based on iron−sulfur complexes
Source: Commun Chem. 2023 Jul 7;6:146. doi: 10.1038/s42004-023-00942-1 (PMC10328931; doi:10.1038/s42004-023-00942-1)

checkCIF/PLATON report

You have not supplied any structure factors. As a result the full set of tests cannot be run.

THIS REPORT IS FOR GUIDANCE ONLY. IF USED AS PART OF A REVIEW PROCEDURE FOR PUBLICATION, IT SHOULD NOT REPLACE THE EXPERTISE OF AN EXPERIENCED CRYSTALLOGRAPHIC REFEREE.

No syntax errors found. CIF dictionary Interpreting this report

Datablock: 2

```
Bond precision:   Zn-Cl = 0.0005 A         Wavelength=0.71073

Cell:               a=9.3811(8)      b=9.3810(8)      c=7.5537(7)
                    alpha=90         beta=90          gamma=90
Temperature:        120 K

                 Calculated                   Reported
Volume           664.76(10)                   664.76(10)
Space group      P -1                         P -1
Hall group       -P 1                         -P 1
Moiety formula   C8 H20 Cl N4 Zn, Cl          ?
Sum formula      C8 H20 Cl2 N4 Zn             C8 H20 Cl2 N4 Zn
Mr               308.57                       308.55
Dx,g cm-3        1.542                        1.541
Z                2                            2
Mu (mm-1)        2.226                        2.226
F000             320.0                        320.0
F000'            321.20
h,k,lmax         12,12,9                      12,12,9
Nref             3029                         2958
Tmin,Tmax        0.723,0.716
Tmin'            0.709

Correction method= Not given

Data completeness= 0.977           Theta(max)= 27.442

                                                       wR2(reflections)=
R(reflections)= 0.0257( 2558)                          0.0655( 2958)
S = 1.037                 Npar= 217
```

The following ALERTS were generated. Each ALERT has the format
test-name_ALERT_alert-type_alert-level.
Click on the hyperlinks for more details of the test.

Alert level C

```
PLAT052_ALERT_1_C Info on Absorption Correction Method   Not Given     Please Do !
PLAT155_ALERT_4_C The Triclinic Unitcell is NOT Reduced .........     Please Do !
```

Alert level G

```
PLAT007_ALERT_5_G Number of Unrefined Donor-H Atoms .............          8 Report
PLAT093_ALERT_1_G No s.u.'s on H-positions, Refinement Reported as      mixed Check
PLAT104_ALERT_1_G The Reported Crystal System is Inconsistent with        P-1 Check
PLAT144_ALERT_4_G s.u. on alpha    Small or Missing .............     0.0000 Degree
PLAT145_ALERT_4_G s.u. on beta     Small or Missing .............     0.0000 Degree
PLAT146_ALERT_4_G s.u. on gamma    Small or Missing .............     0.0000 Degree
PLAT301_ALERT_3_G Main Residue  Disorder .............(Resd  1  )        57% Note
PLAT720_ALERT_4_G Number of Unusual/Non-Standard Labels ..........        40 Note
PLAT811_ALERT_5_G No ADDSYM Analysis: Too Many Excluded Atoms ....         ! Info
PLAT883_ALERT_1_G No Info/Value for _atom_sites_solution_primary .     Please Do !
PLAT941_ALERT_3_G Average HKL Measurement Multiplicity ..........        3.5 Low
```

0 **ALERT level A** = Most likely a serious problem - resolve or explain
0 **ALERT level B** = A potentially serious problem, consider carefully
2 **ALERT level C** = Check. Ensure it is not caused by an omission or oversight
11 **ALERT level G** = General information/check it is not something unexpected

4 ALERT type 1 CIF construction/syntax error, inconsistent or missing data
0 ALERT type 2 Indicator that the structure model may be wrong or deficient
2 ALERT type 3 Indicator that the structure quality may be low
5 ALERT type 4 Improvement, methodology, query or suggestion
2 ALERT type 5 Informative message, check

It is advisable to attempt to resolve as many as possible of the alerts in all categories. Often the minor alerts point to easily fixed oversights, errors and omissions in your CIF or refinement strategy, so attention to these fine details can be worthwhile. In order to resolve some of the more serious problems it may be necessary to carry out additional measurements or structure refinements. However, the purpose of your study may justify the reported deviations and the more serious of these should normally be commented upon in the discussion or experimental section of a paper or in the "special_details" fields of the CIF. checkCIF was carefully designed to identify outliers and unusual parameters, but every test has its limitations and alerts that are not important in a particular case may appear. Conversely, the absence of alerts does not guarantee there are no aspects of the results needing attention. It is up to the individual to critically assess their own results and, if necessary, seek expert advice.

Publication of your CIF in IUCr journals

A basic structural check has been run on your CIF. These basic checks will be run on all CIFs submitted for publication in IUCr journals (*Acta Crystallographica*, *Journal of Applied Crystallography*, *Journal of Synchrotron Radiation*); however, if you intend to submit to *Acta Crystallographica Section C* or *E* or *IUCrData*, you should make sure that full publication checks are run on the final version of your CIF prior to submission.

Publication of your CIF in other journals

Please refer to the *Notes for Authors* of the relevant journal for any special instructions relating to CIF submission.

PLATON version of 18/05/2022; check.def file version of 17/05/2022

Datablock 2 - ellipsoid plot

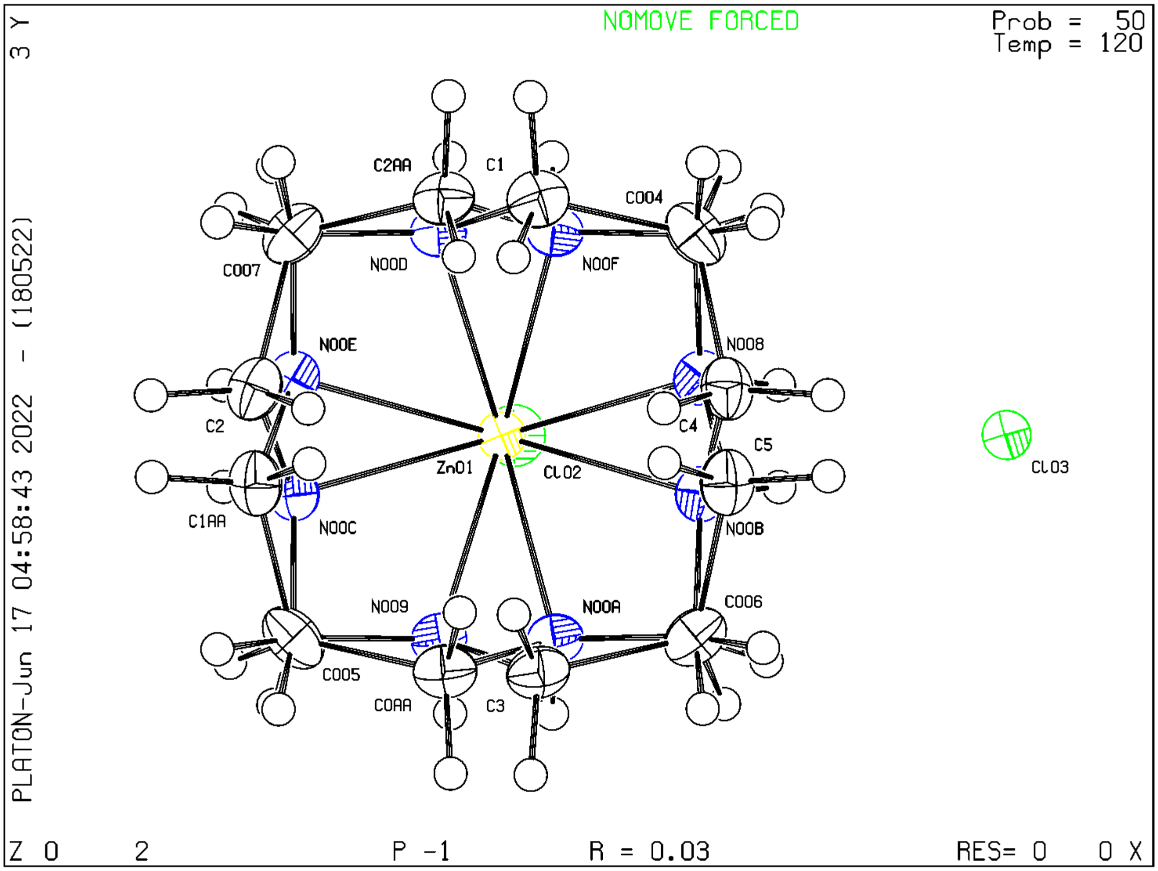

Supplement: Supplementary file 11 — Supplementary Data 8 [file 42004_2023_942_MOESM11_ESM.pdf]
