# Supplementary material for: Order–order assembly transition-driven polyamines detection based on iron−sulfur complexes
Source: Commun Chem. 2023 Jul 7;6:146. doi: 10.1038/s42004-023-00942-1 (PMC10328931; doi:10.1038/s42004-023-00942-1)

checkCIF/PLATON report

You have not supplied any structure factors. As a result the full set of tests cannot be run.

THIS REPORT IS FOR GUIDANCE ONLY. IF USED AS PART OF A REVIEW PROCEDURE FOR PUBLICATION, IT SHOULD NOT REPLACE THE EXPERTISE OF AN EXPERIENCED CRYSTALLOGRAPHIC REFEREE.

No syntax errors found. CIF dictionary Interpreting this report

Datablock: ZHL_tw

Bond precision: C-C = 0.0034 A Wavelength=0.71073

Cell:	a=6.9504(8)	b=9.1243(10)	c=16.525(3)
	alpha=90	beta=90.067(6)	gamma=90
Temperature:	295 K		

	Calculated	Reported
Volume	1048.0(3)	1048.0(2)
Space group	I 2/a	I 2/a
Hall group	-I 2ya	-I 2ya
Moiety formula	C10 H8 Cl2 Fe N2	?
Sum formula	C10 H8 Cl2 Fe N2	C10 H8 Cl2 Fe N2
Mr	282.93	282.93
Dx,g cm-3	1.793	1.793
Z	4	4
Mu (mm-1)	1.910	1.910
F000	568.0	568.0
F000'	570.66	
h,k,lmax	9,11,21	9,11,21
Nref	1204	1201
Tmin,Tmax	0.689,0.751	
Tmin'	0.676	

Correction method= Not given

Data completeness= 0.998 Theta(max)= 27.538

R(reflections)= 0.0519(1066) wR2(reflections)= 0.1448(1201)

S = 1.117 Npar= 58

The following ALERTS were generated. Each ALERT has the format
test-name_ALERT_alert-type_alert-level.
Click on the hyperlinks for more details of the test.

Alert level C

PLAT052_ALERT_1_C Info on Absorption Correction Method   Not Given   Please Do !

Alert level G

PLAT004_ALERT_5_G Polymeric Structure Found with Maximum Dimension   1 Info
PLAT232_ALERT_2_G Hirshfeld Test Diff (M-X) Fe00   --Cl0A   .   7.6 s.u.
PLAT720_ALERT_4_G Number of Unusual/Non-Standard Labels ....   12 Note
PLAT794_ALERT_5_G Tentative Bond Valency for Fe00   (II)   .   1.83 Info
PLAT870_ALERT_4_G ALERTS Related to Twinning Effects Suppressed ..   ! Info
PLAT883_ALERT_1_G No Info/Value for _atom_sites_solution_primary .   Please Do !
PLAT941_ALERT_3_G Average HKL Measurement Multiplicity ....   1.0 Low
PLAT965_ALERT_2_G The SHELXL WEIGHT Optimisation has not Converged   Please Check

0 **ALERT level A** = Most likely a serious problem - resolve or explain
0 **ALERT level B** = A potentially serious problem, consider carefully
1 **ALERT level C** = Check. Ensure it is not caused by an omission or oversight
8 **ALERT level G** = General information/check it is not something unexpected

2 ALERT type 1 CIF construction/syntax error, inconsistent or missing data
2 ALERT type 2 Indicator that the structure model may be wrong or deficient
1 ALERT type 3 Indicator that the structure quality may be low
2 ALERT type 4 Improvement, methodology, query or suggestion
2 ALERT type 5 Informative message, check

It is advisable to attempt to resolve as many as possible of the alerts in all categories. Often the minor alerts point to easily fixed oversights, errors and omissions in your CIF or refinement strategy, so attention to these fine details can be worthwhile. In order to resolve some of the more serious problems it may be necessary to carry out additional measurements or structure refinements. However, the purpose of your study may justify the reported deviations and the more serious of these should normally be commented upon in the discussion or experimental section of a paper or in the "special_details" fields of the CIF. checkCIF was carefully designed to identify outliers and unusual parameters, but every test has its limitations and alerts that are not important in a particular case may appear. Conversely, the absence of alerts does not guarantee there are no aspects of the results needing attention. It is up to the individual to critically assess their own results and, if necessary, seek expert advice.

Publication of your CIF in IUCr journals

A basic structural check has been run on your CIF. These basic checks will be run on all CIFs submitted for publication in IUCr journals (*Acta Crystallographica*, *Journal of Applied Crystallography*, *Journal of Synchrotron Radiation*); however, if you intend to submit to *Acta Crystallographica Section C* or *E* or *IUCrData*, you should make sure that full publication checks are run on the final version of your CIF prior to submission.

Publication of your CIF in other journals

Please refer to the *Notes for Authors* of the relevant journal for any special instructions relating to CIF submission.

PLATON version of 18/05/2022; check.def file version of 17/05/2022

Datablock ZHL_tw - ellipsoid plot

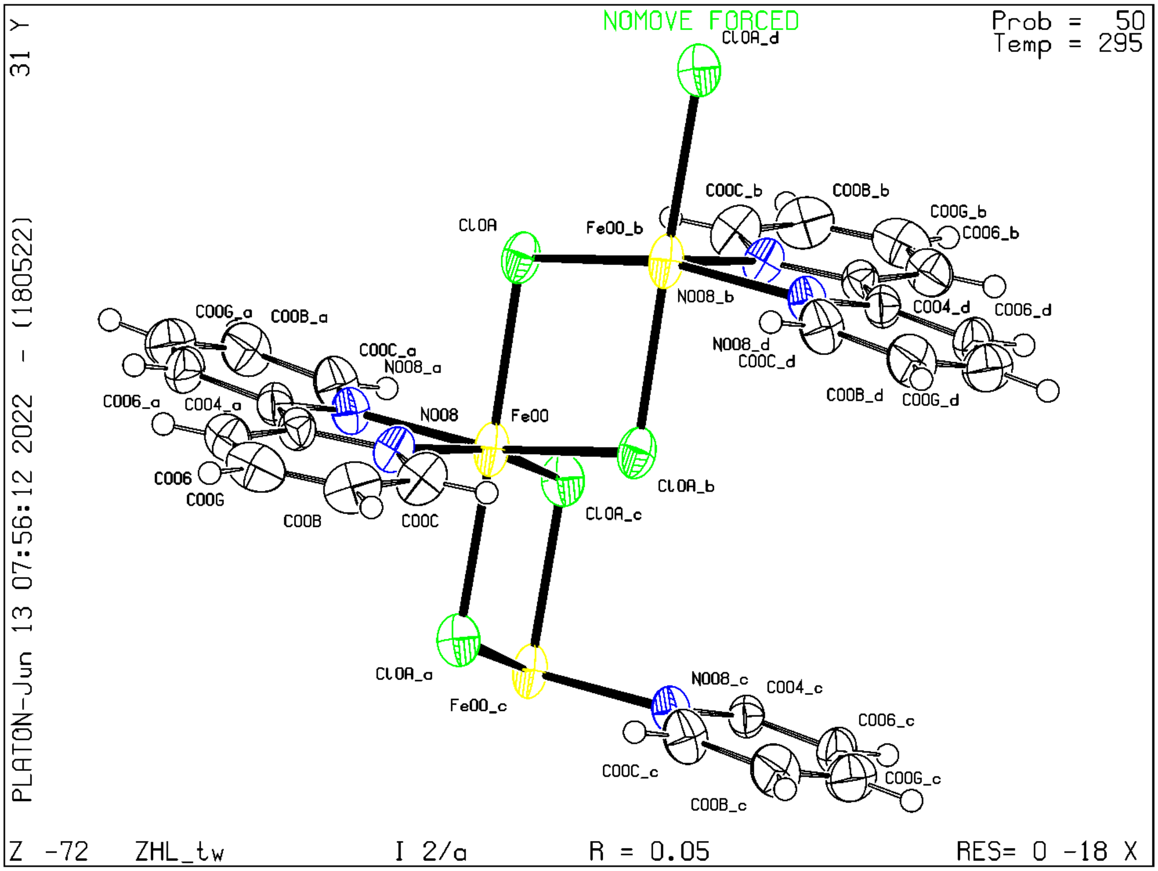

Supplement: Supplementary file 12 — Supplementary Data 9 [file 42004_2023_942_MOESM12_ESM.pdf]
