# Supplementary material for: Order–order assembly transition-driven polyamines detection based on iron−sulfur complexes
Source: Commun Chem. 2023 Jul 7;6:146. doi: 10.1038/s42004-023-00942-1 (PMC10328931; doi:10.1038/s42004-023-00942-1)

checkCIF/PLATON report

Structure factors have been supplied for datablock(s) zhl

THIS REPORT IS FOR GUIDANCE ONLY. IF USED AS PART OF A REVIEW PROCEDURE FOR PUBLICATION, IT SHOULD NOT REPLACE THE EXPERTISE OF AN EXPERIENCED CRYSTALLOGRAPHIC REFEREE.

No syntax errors found. CIF dictionary Interpreting this report

Datablock: zhl

Bond precision:	Mn-Cl = 0.0004 A		Wavelength=0.71073
Cell:	a=9.4344(3)	b=9.4345(3)	c=7.7669(3)
	alpha=89.9990	beta=90.0010	gamma=90.0010
Temperature:	295 K		

	Calculated	Reported
Volume	691.32(4)	691.32(4)
Space group	P -1	P -1
Hall group	-P 1	-P 1
Moiety formula	C8 H20 Cl Mn N4, Cl	C8 H20 Cl Mn N4, Cl
Sum formula	C8 H20 Cl2 Mn N4	C8 H20 Cl2 Mn N4
Mr	298.12	298.12
Dx,g cm-3	1.432	1.432
Z	2	2
Mu (mm-1)	1.319	1.319
F000	310.0	310.0
F000'	311.30	
h,k,lmax	11,11,9	11,11,9
Nref	2429	2422
Tmin,Tmax	0.776,0.820	0.622,0.745
Tmin'	0.761	

Correction method= # Reported T Limits: Tmin=0.622 Tmax=0.745
AbsCorr = MULTI-SCAN

Data completeness= 0.997 Theta(max)= 24.938

R(reflections)= 0.0191(2107) wR2(reflections)= 0.0447(2422)

S = 1.055 Npar= 209

The following ALERTS were generated. Each ALERT has the format
test-name_ALERT_alert-type_alert-level.
Click on the hyperlinks for more details of the test.

Alert level C

```
PLAT155_ALERT_4_C The Triclinic Unitcell is NOT Reduced ..........     Please Do !
PLAT911_ALERT_3_C Missing FCF Refl Between Thmin & STh/L=    0.593          4 Report
```

Alert level G

```
PLAT007_ALERT_5_G Number of Unrefined Donor-H Atoms .............          8 Report
PLAT144_ALERT_4_G s.u. on alpha    Small or Missing .............     0.0000 Degree
PLAT145_ALERT_4_G s.u. on beta     Small or Missing .............     0.0000 Degree
PLAT146_ALERT_4_G s.u. on gamma    Small or Missing .............     0.0000 Degree
PLAT232_ALERT_2_G Hirshfeld Test Diff (M-X)  Mn01     --Cl03     .       12.7 s.u.
PLAT301_ALERT_3_G Main Residue  Disorder .............(Resd  1  )        57% Note
PLAT720_ALERT_4_G Number of Unusual/Non-Standard Labels ..........        39 Note
PLAT767_ALERT_4_G INS Embedded LIST 6 Instruction Should be LIST 4     Please Check
PLAT811_ALERT_5_G No ADDSYM Analysis: Too Many Excluded Atoms ....         ! Info
PLAT909_ALERT_3_G Percentage of I>2sig(I) Data at Theta(Max) Still        64% Note
PLAT910_ALERT_3_G Missing # of FCF Reflection(s) Below Theta(Min).          2 Note
PLAT933_ALERT_2_G Number of HKL-OMIT Records in Embedded .res File          1 Note
PLAT941_ALERT_3_G Average HKL Measurement Multiplicity ...........        4.4 Low
```

0 **ALERT level A** = Most likely a serious problem - resolve or explain
0 **ALERT level B** = A potentially serious problem, consider carefully
2 **ALERT level C** = Check. Ensure it is not caused by an omission or oversight
13 **ALERT level G** = General information/check it is not something unexpected

0 ALERT type 1 CIF construction/syntax error, inconsistent or missing data
2 ALERT type 2 Indicator that the structure model may be wrong or deficient
5 ALERT type 3 Indicator that the structure quality may be low
6 ALERT type 4 Improvement, methodology, query or suggestion
2 ALERT type 5 Informative message, check

It is advisable to attempt to resolve as many as possible of the alerts in all categories. Often the minor alerts point to easily fixed oversights, errors and omissions in your CIF or refinement strategy, so attention to these fine details can be worthwhile. In order to resolve some of the more serious problems it may be necessary to carry out additional measurements or structure refinements. However, the purpose of your study may justify the reported deviations and the more serious of these should normally be commented upon in the discussion or experimental section of a paper or in the "special_details" fields of the CIF. checkCIF was carefully designed to identify outliers and unusual parameters, but every test has its limitations and alerts that are not important in a particular case may appear. Conversely, the absence of alerts does not guarantee there are no aspects of the results needing attention. It is up to the individual to critically assess their own results and, if necessary, seek expert advice.

Publication of your CIF in IUCr journals

A basic structural check has been run on your CIF. These basic checks will be run on all CIFs submitted for publication in IUCr journals (*Acta Crystallographica*, *Journal of Applied Crystallography*, *Journal of Synchrotron Radiation*); however, if you intend to submit to *Acta Crystallographica Section C* or *E* or *IUCrData*, you should make sure that full publication checks are run on the final version of your CIF prior to submission.

Publication of your CIF in other journals

Please refer to the *Notes for Authors* of the relevant journal for any special instructions relating to CIF submission.

PLATON version of 18/05/2022; check.def file version of 17/05/2022

Datablock zhl - ellipsoid plot

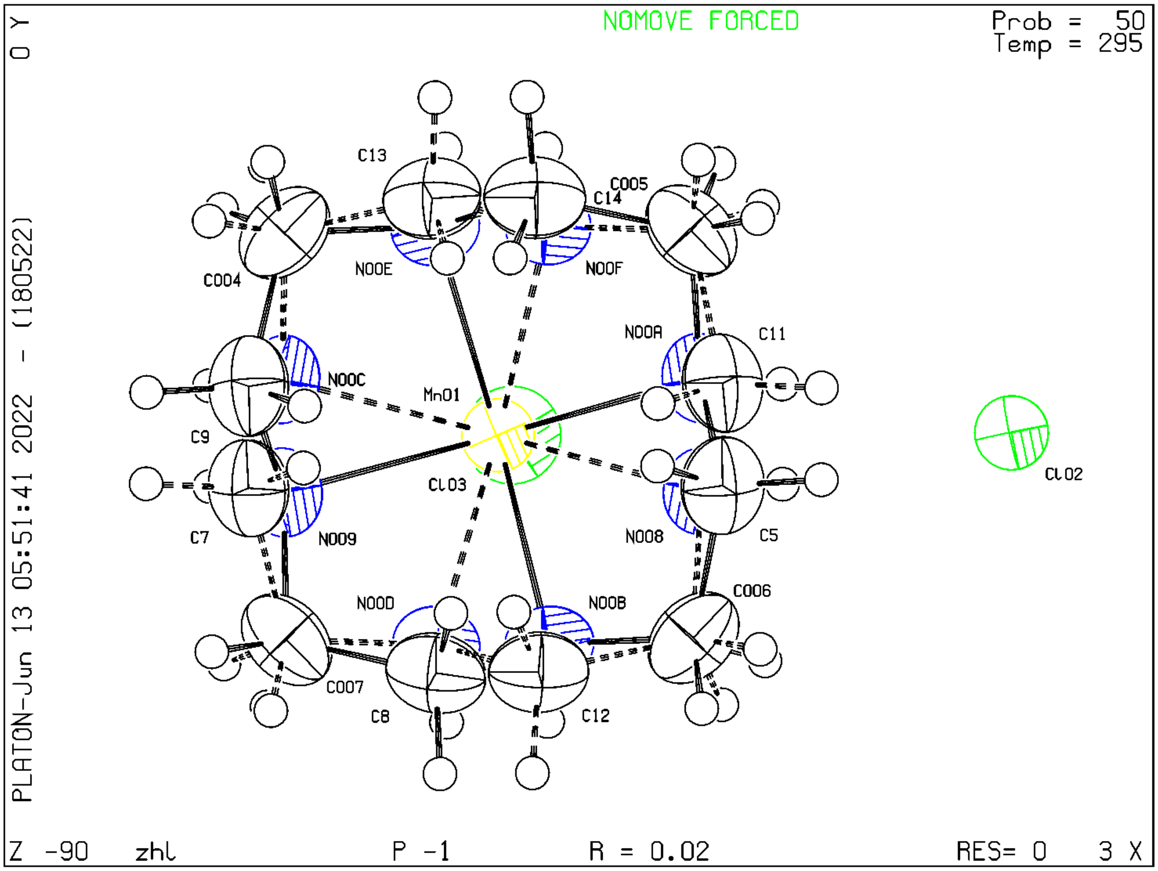

Supplement: Supplementary file 13 — Supplementary Data 10 [file 42004_2023_942_MOESM13_ESM.pdf]
